# Genetic Analysis of the Cardiac Methylome at Single Nucleotide Resolution in a Model of Human Cardiovascular Disease

**DOI:** 10.1371/journal.pgen.1004813

**Published:** 2014-12-04

**Authors:** Michelle D. Johnson, Michael Mueller, Martyna Adamowicz-Brice, Melissa J. Collins, Pascal Gellert, Klio Maratou, Prashant K. Srivastava, Maxime Rotival, Shahena Butt, Laurence Game, Santosh S. Atanur, Nicholas Silver, Penny J. Norsworthy, Sarah R. Langley, Enrico Petretto, Michal Pravenec, Timothy J. Aitman

**Affiliations:** 1 Physiological Genomics and Medicine Group, MRC Clinical Sciences Centre, London, United Kingdom; 2 National Heart and Lung Institute, Imperial College, London, United Kingdom; 3 Institute of Clinical Sciences, Imperial College, London, United Kingdom; 4 Integrative Genomics and Medicine Group, MRC Clinical Sciences Centre, London, United Kingdom; 5 Genomics Core Laboratory, MRC Clinical Sciences Centre, London, United Kingdom; 6 Institute of Physiology, Academy of Sciences of the Czech Republic, Prague, Czech Republic; 7 Institute of Biology and Medical Genetics, 1st Medical Faculty, Charles University, Prague, Czech Republic; University of Oxford, United Kingdom

## Abstract

Epigenetic marks such as cytosine methylation are important determinants of cellular and whole-body phenotypes. However, the extent of, and reasons for inter-individual differences in cytosine methylation, and their association with phenotypic variation are poorly characterised. Here we present the first genome-wide study of cytosine methylation at single-nucleotide resolution in an animal model of human disease. We used whole-genome bisulfite sequencing in the spontaneously hypertensive rat (SHR), a model of cardiovascular disease, and the Brown Norway (BN) control strain, to define the genetic architecture of cytosine methylation in the mammalian heart and to test for association between methylation and pathophysiological phenotypes. Analysis of 10.6 million CpG dinucleotides identified 77,088 CpGs that were differentially methylated between the strains. In F1 hybrids we found 38,152 CpGs showing allele-specific methylation and 145 regions with parent-of-origin effects on methylation. *Cis*-linkage explained almost 60% of inter-strain variation in methylation at a subset of loci tested for linkage in a panel of recombinant inbred (RI) strains. Methylation analysis in isolated cardiomyocytes showed that in the majority of cases methylation differences in cardiomyocytes and non-cardiomyocytes were strain-dependent, confirming a strong genetic component for cytosine methylation. We observed preferential nucleotide usage associated with increased and decreased methylation that is remarkably conserved across species, suggesting a common mechanism for germline control of inter-individual variation in CpG methylation. In the RI strain panel, we found significant correlation of CpG methylation and levels of serum chromogranin B (CgB), a proposed biomarker of heart failure, which is evidence for a link between germline DNA sequence variation, CpG methylation differences and pathophysiological phenotypes in the SHR strain. Together, these results will stimulate further investigation of the molecular basis of locally regulated variation in CpG methylation and provide a starting point for understanding the relationship between the genetic control of CpG methylation and disease phenotypes.

## Author Summary

Epigenetic marks provide information that is not encoded in the primary DNA sequence itself but in modifications of genomic DNA and of the associated proteins. Methylation of genomic DNA at cytosine residues is an important epigenetic modification that is associated with developmental processes, carcinogenesis and other diseases. Genome-wide extent of, and reasons for inter-individual differences in cytosine methylation, and their association with phenotypic variation are poorly characterised. To address these questions we have determined and compared the genome-wide methylation patterns in heart tissue of two inbred rat strains, the spontaneously hypertensive rat, an animal model of human disease and a control rat strain. Comparison of methylation differences between genetically identical animals from the same strain and differences between animals from different strains allowed us to quantify association of epigenetic and genetic differences. We show that differences in an individual's germline DNA sequence are important determinants of the variability in methylation between individuals. Comparison with previous reports implicates common mechanisms for regulation of cytosine methylation that are highly conserved across species. Finally, we find correlation between a proposed blood biomarker for heart failure and variation in DNA methylation, suggesting a link between germline DNA sequence variation, methylation and a disease-related phenotype.

## Introduction

Cytosine methylation at CpG dinucleotides is a key epigenetic mark with an essential role in regulating gene expression and other cellular and whole body phenotypes. While the molecular mechanisms for *de novo* and maintenance methylation of CpG cytosines are well established [1]–[3], allele-specific influences on CpG methylation have been documented [4]–[12], and association of genotype and epigenotype has been shown in plants recently [13], the extent of, and reasons for inter-individual differences in cytosine methylation, and their association with phenotypic variation in mammals are poorly characterised.

Isogenic inbred lines provide a powerful platform for establishing relationships between the germline genome and downstream phenotypes. The ability to breed experimental crosses, to study multiple genetically identical animals, to minimise environmental influences and to sample tissues as required are significant advantages over comparable studies in humans. We have generated extensive genetic, genomic and physiological resources in our studies of the SHR, including expression datasets [14], [15] and the SHR genome sequence [16], [17] that have led to identification of several genes underlying pathophysiological traits in the SHR strain [18]–[22].

Given these resources and the relevance of SHR cardiac phenotypes to related human disorders [18], [19], we sought to investigate cytosine methylation in the SHR heart. We applied whole-genome bisulfite sequencing (WGBS) to multiple isogenic animals from the SHR and BN strains, to test the hypothesis that inter-individual variation in CpG dinucleotide methylation is regulated by genomic DNA sequence and may be important in the development of other genetically determined SHR phenotypes.

## Results

We generated WGBS datasets from the left ventricles of four BN and four SHR rats (European Nucleotide Archive accession number ERP002215), producing a total of 1.3 and 1.5 billion mapped reads in each strain. This was equivalent to 40–50× mean strand-specific read coverage per strain, and >28× mean coverage after quality filtering and removal of clonal and non-uniquely mapped reads (Table S1). Of the ∼40 million CpG dinucleotides covered by mapped reads on either strand in either strain, we focus here on the 10,614,445 CpG dinucleotides that were sequenced at a coverage depth of at least 5× strand-specific reads across a minimum of three animals per strain (Figure S1).

**Figure 1:**
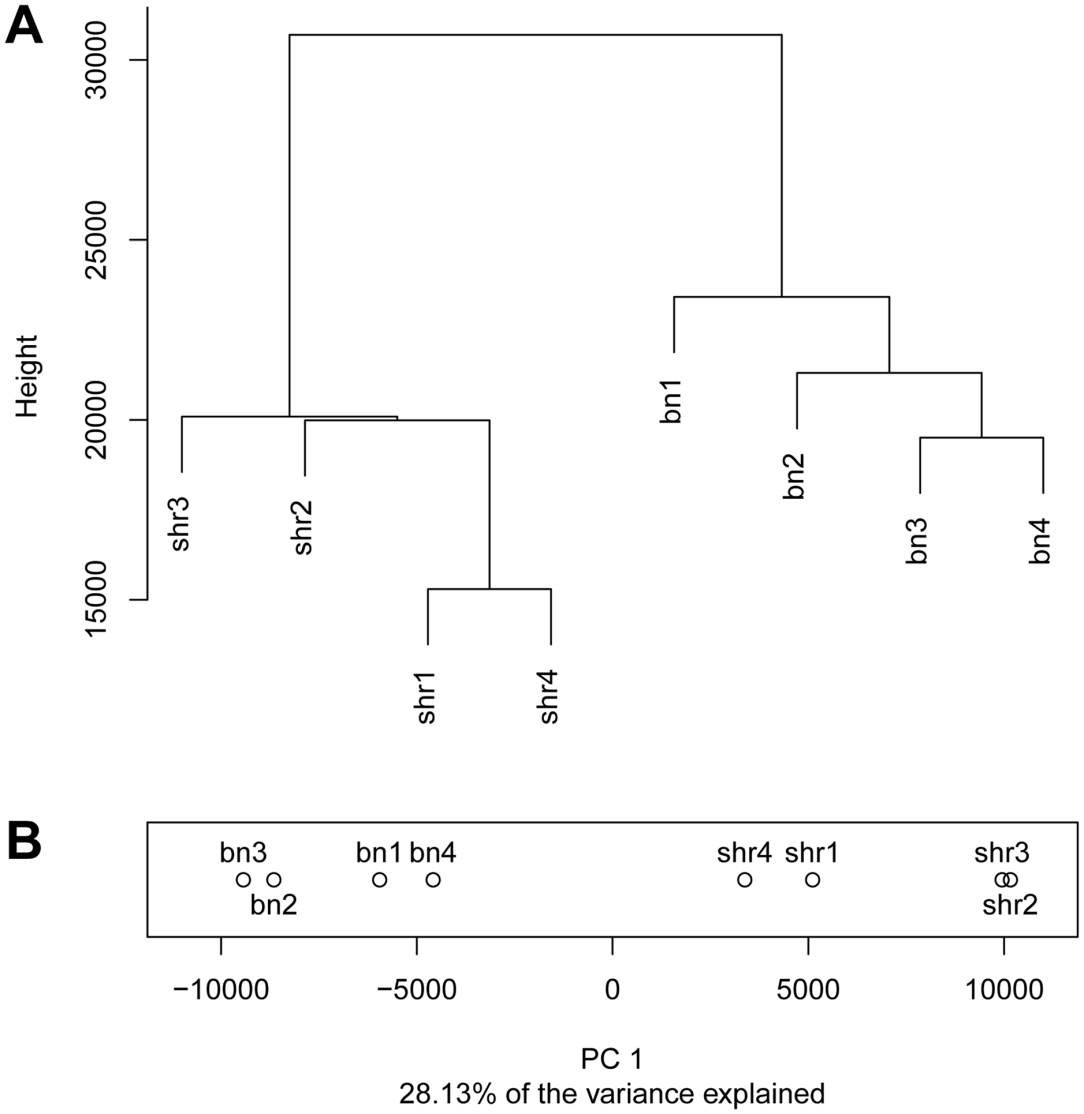
Differential CpG methylation between the BN and SHR strains. A) Dendrogram showing the clustering the CpG methylation profiles obtained in four BN and four SHR rats. B) Projection of methylation profiles onto the first principal component. Numbers after the strain label denote biological replicates.

Hierarchical clustering of the methylation profiles showed that CpG dinucleotide methylation was more variable between strains than within strains. The average distance between methylation profiles of animals within each strain (average distance BN = 5,255, average distance SHR = 5,430) was ∼5 times lower than the average distance between animals across strains (average distance BN-SHR = 24,281) (Figure 1A). To quantify the contribution of strain specific differences to the variation in methylation measurements we carried out a principal component analysis which showed that the first principal component which separates the methylation profiles by strain explains 28.13% of the total variance (Figure 1B). No differences in global methylation levels between the strains were detected by pyrosequencing (Figure S2). These data suggested that differences in CpG dinucleotide methylation may be at least in part dependent on genomic sequence variation. To test this hypothesis we investigated the relationship between genome-wide inter-strain methylation differences and inter-strain differences in genomic DNA sequence.

First, we defined a set of CpG dinucleotides that were differentially methylated between animals from the SHR and BN strains. Of the 10,614,445 analysed CpG dinucleotides, 77,088 (0.7%) were significantly differentially methylated between the two strains (false discovery rate (FDR) <5%). Of the 77,088 differentially methylated CpGs, 47,775 cluster into 12,128 differentially methylated regions containing between 2 and 213 CpG dinucleotides, while 29,313 are single CpG dinucleotides that are at least 500 bp away from the next differentially methylated CpG dinucleotide (Figure S3). In more than 96% of differentially methylated regions, methylation differences between strains at individual CpGs were in the same direction. In line with previous observations, the large majority of analysed CpG dinucleotides were highly methylated (≥80% methylation) (Figure S4) but a significant fraction of CpG dinucleotides (2.9%) had very low methylation (≤10% methylation). Within CpG islands (CGIs), differentially methylated CpGs were found equally across different genomic features whilst outside CGIs, the proportion of differentially methylated CpGs was increased around the transcription start site (TSS) (Figure S5).

To determine whether differential methylation could be associated with discrete molecular or cellular functions important to the biological differences between SHR and BN rats, we carried out a Gene Ontology analysis of genes overlapping with or in close proximity (within 5000 bp) of differentially methylated regions containing five or more differentially methylated CpGs. Based on these criteria 2,525 differentially methylated regions were associated with 1,283 genes. This set of genes showed strong enrichment for gene products localised to the plasma membrane (p = 1.2×10^−14^) or involved in neuron differentiation (p = 2.9×10^−4^) or cell communication (p = 2.9×10^−4^) (Table S2).

### Genetic analysis of CpG dinucleotide methylation

To validate WGBS methylation calls and to define a set of differentially methylated CpG dinucleotides for genetic analysis, we carried out Sanger sequencing and multiplexed Illumina sequencing of PCR products generated from bisulfite converted genomic DNA. We selected a set of CpG dinucleotides (Table S3) in 40 regions (out of the 12,128 DMRs detected in the parental strains) that showed between 1% and 93% differential methylation between BN and SHR, were located at varying distances from TSSs (the majority were within 3 kb of the TSS) and were within a variety of genomic elements. By Sanger sequencing in BN and SHR, there were 81 scoreable CpG dinucleotides for which 70 were covered in the WGBS data set. By multiplexed Fluidigm amplification and Illumina sequencing, there were 131 scoreable CpG dinucleotides, all of which were covered in the WGBS dataset (Table S3). CpG methylation measured from bisulfite-converted PCR-amplified products was highly concordant with WGBS methylation data, both for the Sanger and for the Fluidigm/Illumina-sequenced PCR products (r_Sanger_ = 0.86, p<10^−4^, Figure S6A; r_Fluidigm_ = 0.93, p<10^−4^, Figure S6B).

**Figure 2:**
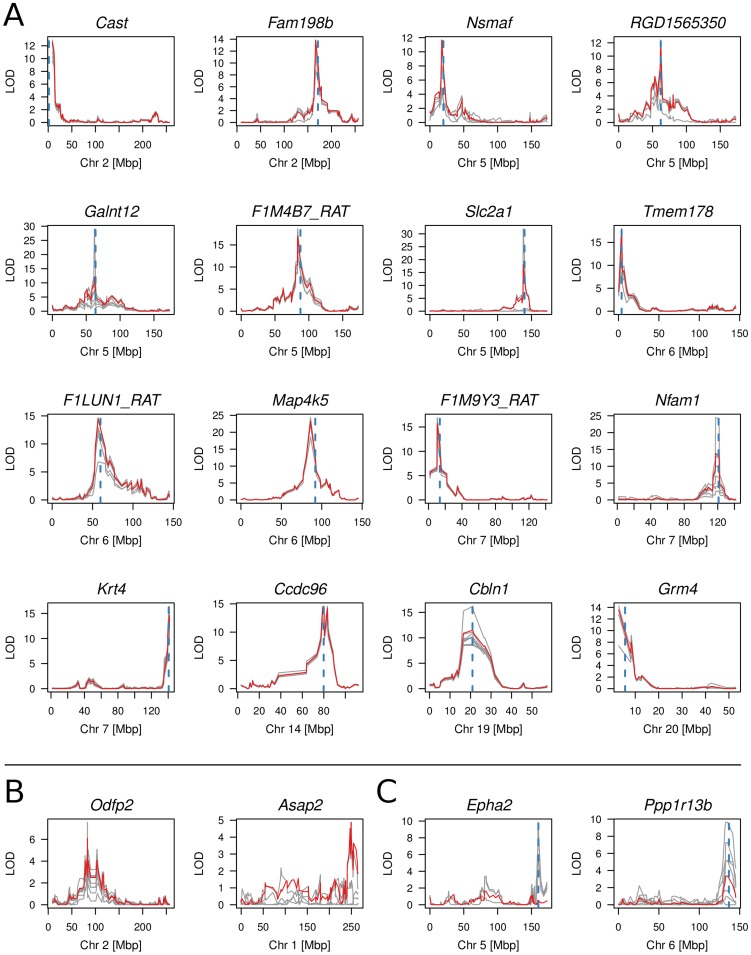
Multi-point linkage plots of meth-QTLs. (A) Linkage plots of 16 *cis* meth-QTLs with LOD scores >10 for linkage to mean CpG methylation. LOD scores for linkage of average amplicon methylation levels are shown in red, LOD scores for linkage of individual CpG di-nucleotides are shown in grey. The peak of linkage is indicated by a dashed, blue vertical line. (B) Linkage plot for the *Odfp2* and *Asap2* meth-QTLs which showed significant *trans*-linkages to chromosome 2 and 1 respectively whilst the amplicons reside on chromosomes 3 and 6. (C) Linkage plots of *Epha2* and *Ppp1r13b* for which the average methylation did not show significant linkage but individual CpG dinucleotides mapped in *cis*.

**Figure 3:**
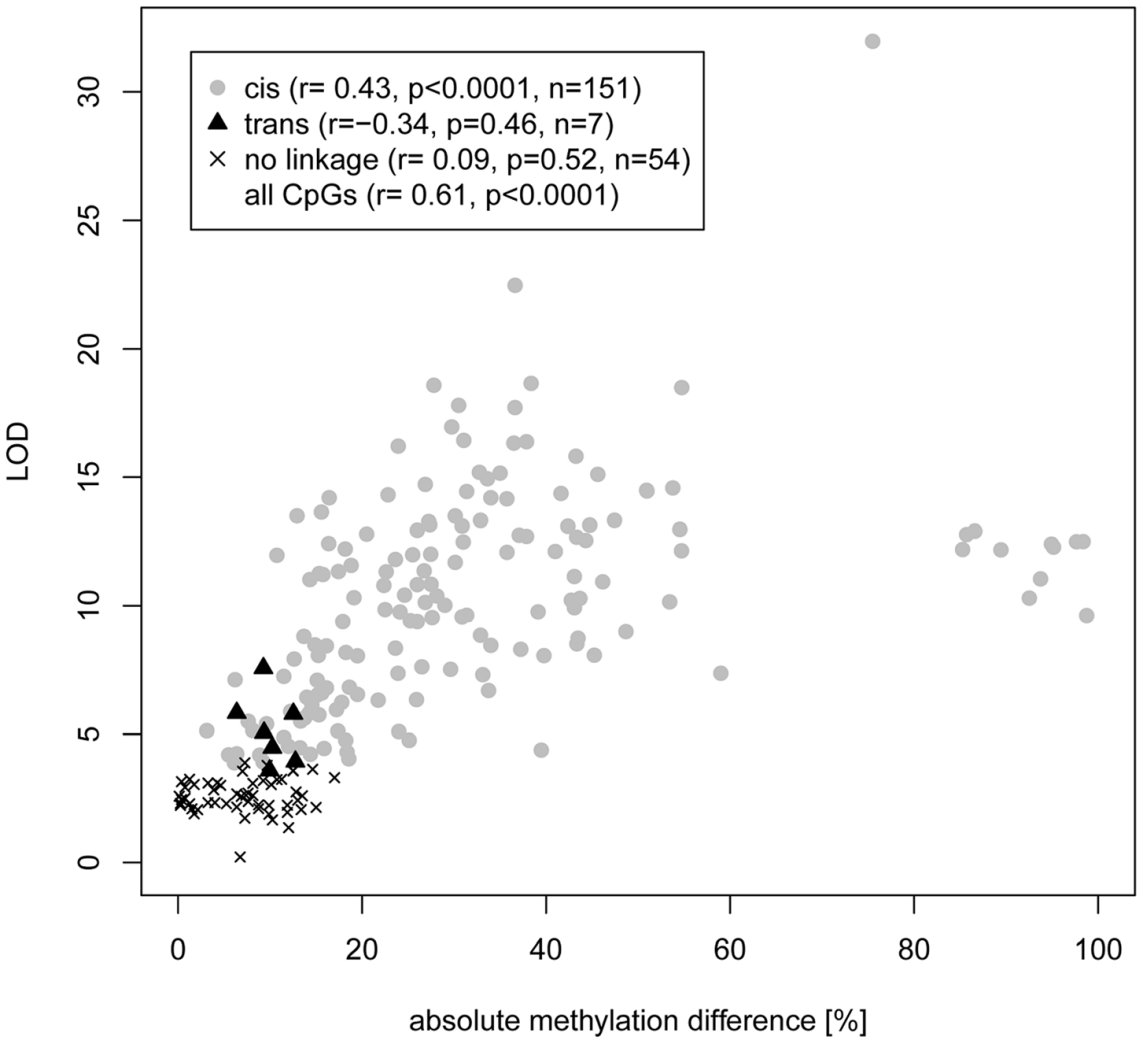
Correlation of CpG cytosine meth-QTL linkage and difference in CpG methylation between parental strains. Methylation data were obtained from PCR amplification of bisulfite-treated DNA followed by Sanger or Illumina sequencing in parental and RI strains. CpGs were classified according to linkage type (*cis*, *trans*, no significant linkage) and LOD scores were plotted against the absolute value of the difference in methylation between parental strains. No linkage refers to CpGs for which permutation-based p≥0.05. Correlation was assessed by Pearson *r* value. The cluster of CpGs with >80% difference in methylation are all from a single amplicon selected because of high differential methylation in the parental strains.

We next sought to map the genetic determinants of CpG dinucleotide methylation of the 212 CpG dinucleotides residing within the 40 amplicons in the BN- and SHR-derived BXH/HXB panel of recombinant inbred (RI) strains [23], [24], using methylation percentage as a quantitative trait. Mean cytosine methylation for 32 of the 40 amplicons mapped in *cis* (LOD scores 4.8–23.3) and two in *trans* (LOD 4.9 and 6.1) (Table 1, Figure 2, Figure S7). The remaining six amplicons showed no linkage for mean CpG methylation although individual CpG dinucleotides in three of these amplicons (*Epha2* [ENSRNOG00000009222], *Ppp1r13b* [ENSRNOG00000012653], *Tbc1d30* [ENSRNOG00000023951]) showed strong *cis* linkage (LOD 5.1–9.9; Fig. 2C, Figure S7, Table S3). Individual CpG dinucleotides within all three of these amplicons showed opposing allelic effects (Table S3), which was validated by amplicon sequencing in the parentals (Figure S8), explaining the lack of linkage to mean CpG methylation across these amplicons. Individual CpGs within other amplicons generally showed similar quantitative differences and directional change (Table S3). The strength of linkage to methylation at individual CpG cytosines was highly dependent on the difference in methylation between the parental strains (Figure 3). We assessed the heritability of cytosine methylation by segregation in the RI strains as previously described [15] for the 32 amplicons for which cytosine methylation was regulated in *cis* and found that the heritability of mean amplicon methylation ranged from 12% to 99%, with mean heritability being 62% (SD 22%). We also assessed heritability by calculating the proportion of the linkage signals at these 32 loci that was explained by *cis* linkage and found that, on average 58% (SD 23%) of the total phenotypic variability in mean methylation in the RI strains at these loci was explained by *cis* linkage. Taken together, these data imply that methylation percentage at most differentially methylated loci are regulated in *cis*. In addition, the observed large sizes of the *cis*-QTL effects suggest that these methylation phenotypes are essentially under the control of a single locus, defined here as the extent of the linkage region, and are strongly regulated in *cis*.

**Table 1:** Meth-QTL amplicon linkage and methylation.

Gene ID^a^	CGI^b^	WGBS BN^c^	WGBS SHR^c^	WGBS difference^c^	Meth-QTL status^d^	LOD score
*Bcl11b*	no	33	61	−28	*cis*	10.4
*Cast*	yes	95	17	78	*cis*	12.6
*Cbln1*	yes	13	45	−32	*cis*	11.5
*Ccdc96*	yes	70	7	63	*cis*	14.2
*Cdkn2b*	no	17	29	−13	*cis*	4.8
*Ddhd1*	yes	49	71	−22	*cis*	10.4
*E9PSL5_RAT*	no	65	75	−10	*cis*	7.8
*F1LUN1_RAT*	no	86	64	22	*cis*	14.5
*F1M4B7_RAT*	no	68	40	28	*cis*	17.0
*F1M804_RAT*	no	74	66	8	*cis*	5.2
*F1M9Y3_RAT*	yes	3	23	−20	*cis*	15.7
*Fam198b*	no	75	54	21	*cis*	13.9
*Fgd6*	no	83	70	13	*cis*	6.5
*Galnt12*	yes	40	60	−20	*cis*	12.1
*Grm4*	no	46	23	23	*cis*	13.6
*Krt4*	yes	82	39	43	*cis*	14.6
*LOC100360843*	yes	24	67	−43	*cis*	8.7
*LOC100365068*	no	85	57	28	*cis*	10.8
*Ly6e*	no	8	45	−37	*cis*	9.6
*Map4k5*	no	17	56	−39	*cis*	23.3
*Naprt1*	yes	9	32	−23	*cis*	9.8
*Nfam1*	no	78	59	19	*cis*	13.8
*Nsmaf*	no	68	55	13	*cis*	11.7
*Nsmce2*	no	82	72	10	*cis*	9.5
*RGD1564053*	no	64	76	−12	*cis*	7.3
*RGD1565350*	no	47	32	15	*cis*	12.2
*Rragc* (F3)	no	51	69	−18	*cis*	9.2
*Rragc* (F4)	yes	38	74	−36	*cis*	11.4
*Slc2a1*	no	82	63	19	*cis*	18.1
*Snapc1*	yes	12	25	−13	*cis*	9.6
*Tmem178*	yes	32	76	−44	*cis*	18.0
*Ttc22*	yes	25	50	−25	*cis*	7.2
*Asap2*	yes	84	91	−7	*trans*	4.9
*Odfp2*	yes	30	49	−19	*trans*	6.1
*Akr1b10*	yes	12	16	−4	NSL	2.9
*BI1_RAT*	yes	17	29	−12	NSL	4.1
*Epha2*	yes	42	34	8	NSL	2.4
*Ppp1r13b*	yes	66	66	0	NSL	3.3
*Rtdr1*	no	10	28	−18	NSL	1.9
*Tbc1d30*	no	27	22	5	NSL	2.9

a Gene ID indicates the closest gene to the amplicon.

b (CGI) within or overlaps with a CpG island.

c (WGBS) whole genome bisulfite sequencing, methylation percentage in BN and SHR strains, or difference between the strains (BN-SHR).

d (NSL) No significant linkage is defined as P>0.05 after a minimum of 1000 permutations of the linkage data.

Assay IDs are provided where there are two identical gene IDs.

The differentially methylated CpG cluster at *Odfp2* [ENSRNOG00000014584] on chromosome 3 was regulated in *trans* by a locus on chromosome 2. Four of the seven protein-coding genes in the 2-LOD linkage confidence interval – or *trans*-regulated methylation quantitative trait locus (meth-QTL) - contain non-synonymous coding sequence variants between BN and SHR and two of the six non-protein coding genes contain sequence variants (Table S4). In an RNA-seq data set that we generated from SHR and BN left ventricle (Table S5, ArrayExpress accession number E-MTAB-1702), we detected expression for 8 of the 13 genes in the 2-LOD confidence interval, of which three showed evidence of differential expression or alternative splicing between BN and SHR (Table S4). None of the 13 genes in the 2-LOD confidence interval nor their mouse or human orthologues had Gene Ontology annotations related to DNA methylation, chromatin status or DNA binding, suggesting a potentially novel mechanism for *trans*-regulated methylation caused by sequence variation or altered expression of one of the protein-coding or non-protein-coding genes in this region. The *trans*-regulated CpG cluster at *Asap2* [ENSRNOG00000006056] on chromosome 6 was regulated by a locus on chromosome 1. The region of linkage contained more than 180 genes, many of which showed either differential expression or coding sequence variation between SHR and BN.

**Figure 4:**
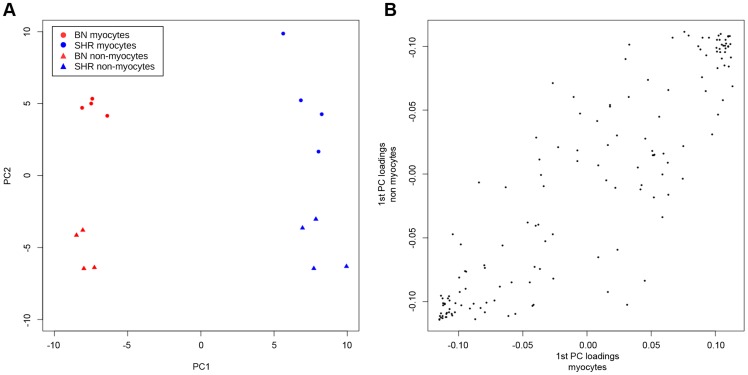
Principal component analysis (PCA) of methylation measurements in cardiomyocyte and non-cardiomyocyte cells isolated from intact SHR and BN cardiac tissue. (A) Projection of samples onto the first two principal components (PC) of the methylation measurements. (B) Comparison of loadings of the 1^st^ PC (strain separation) of PCA of methylation measurements done in each cell population separately.

Because our studies of differential methylation were carried out on intact heart tissue, it is possible that inter-strain differences in methylation were due to differences in cellular composition of the tissue samples rather than inter-strain differences in methylation for a particular cell type. We therefore studied CpG methylation at differentially methylated sites in cardiomyocytes and non-cardiomyocytes isolated from intact SHR and BN heart. Whilst a small proportion of differentially methylated loci showed methylation differences that were in part dependent on cell type, in 69% of cases methylation differences in cardiomyocytes and non-cardiomyocytes were at least in part dependent on strain, and for 77% of CpGs methylation differences were independent of cell type (Figure S9, Table S6). A principal component analysis (PCA) of methylation measurements (Figure 4) confirmed that the variation in methylation is in large part explained by the strain background. The first principal component (PC) which retains 43% of the variance separates samples into BN and SHR while the second PC which explains 20% of the variance distinguishes samples by tissue (Figure 4A). Separate PCA of methylation measurements done in each cell population, showed similar distribution of loadings for the 1^st^ PC (strain difference) in both cell populations (Figure 4B), suggesting similar patterns of strain dependant methylation in the two populations.

### Correlation of variation in CpG methylation and phenotypic variation

We carried out a quantitative trait methylation (QTM) analysis to test for associations between variation in methylation and phenotypic variation. We searched for correlation between average methylation levels at the 40 loci previously tested for genetic linkage (Table S3) and 241 physiological quantitative traits measured across the RI strain panel. We found significant negative correlation (Pearson's *r* = −0.75) of CpG methylation on chr5:143,573,578–143,573,701 and serum chromogranin B (CgB) levels (*P* = 1.27×10^−2^, FDR = 5%). Subsequent QTL mapping for the serum chromogranin B trait located the peak of linkage to chr5:143,426,944 (LOD score = 3.92, *P* = 0.007) which was identical to the peak of linkage of the methylation QTL that correlated with the phenotype.

### Sequence variation near differentially methylated CpG dinucleotides

**Figure 5:**
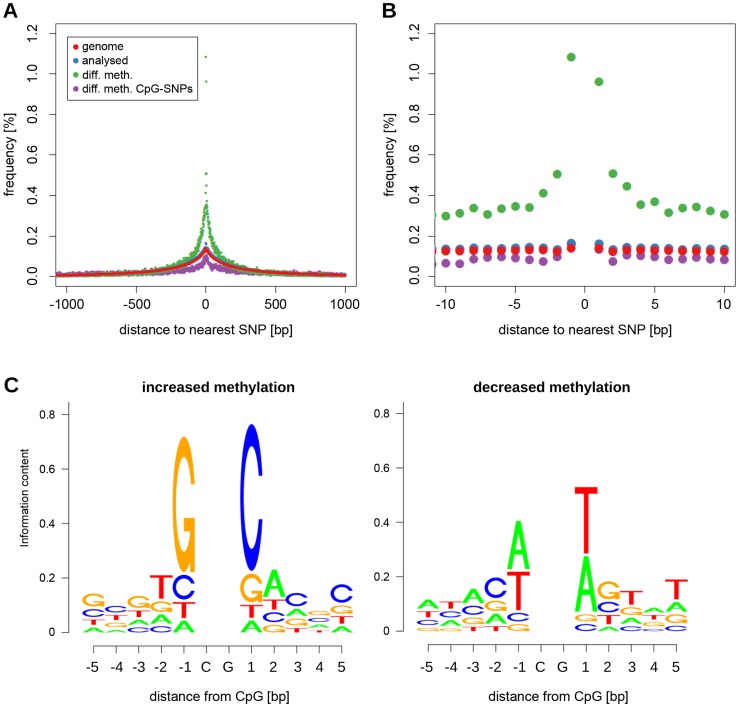
Sequence variation around differentially methylated CpGs. (A) Plot showing the frequency of CpGs with a SNP within 1 kb for all CpG dinucleotides in the genome (red), all CpGs tested for differential methylation (blue), CpGs that showed a significant difference in methylation between BN and SHR rats (green), and differentially methylated CpGs with a CpG-disrupting SNP in the vicinity (purple). In the latter case the distance relates to the nearest CpG-disrupting SNP. (B) same plot as (A) zoomed in to the 10 bp interval around the CpG. (C) Sequence logos showing SNP allele frequencies around differentially methylated CpGs for alleles associated with increased (top) and decreased (bottom) methylation.

To identify genomic variants that may underlie *cis*-regulatory control of CpG dinucleotide methylation, we analysed the distance to the nearest SNP for all differentially methylated CpG dinucleotides. We found an increased frequency (5-fold enrichment) of CpG dinucleotides with closely adjacent SNPs in the set of differentially methylated CpGs compared to the analysed set of CpG dinucleotides and to all CpG dinucleotides in the genome (Figure 5A). SNP enrichment in DNA adjacent to differentially methylated CpG dinucleotides was not due to SNPs that disrupt adjacent CpG dinucleotides because there was no enrichment of CpG-disrupting SNPs at these sites (Figure 5A). There was an increase in SNP frequency starting at around 250 bp from differentially methylated CpGs with a further sharp increase occurring within 5 bp of the differentially methylated CpG dinucleotide (Figure 5B): 58.8% of differentially methylated CpGs were no further then 250 bp away from a SNP, 5.3% had a SNP within 5 bp. SNP proximity was, however, not associated with the extent of differential methylation at the affected CpG dinucleotide (Figure S10).

To investigate further the relationship between local SNP density and differential methylation, we sought to determine whether there was an association between nucleotide preference within 5 bp of differentially methylated CpG dinucleotides and either increased or decreased methylation. We observed preferential nucleotide usage for increased and decreased CpG dinucleotide methylation within this 12 bp window with particularly strong association in the 1–3 bp immediately adjacent to differentially methylated CpGs (Figure 5C). The nucleotide signatures that we identified were very similar to those recently found to be associated with increased and decreased methylation in mouse brain [12]. While the consensus sequence of the nucleotide usage at hypermethylated CpG dinucleotides is identical the consensus sequence at hypomethylated CpGs differs only at a single position (Table S7). To quantify the observed similarity we compared our position frequency matrices with those obtained by Xie et al (personal communication) using the TOMTOM comparison tool [25]. Both the nucleotide usage patterns around hyper- as well as hypomethylated CpGs are highly similar between rat heart and mouse brain (P_hypo_ = 2.76×10^−10^, P_hyper_ = 1.06×10^−10^, Table S7).

After categorising SNP differences between BN and SHR 1 bp up- and 1 bp downstream of the differentially methylated CpG we found that SNPs which changed adenines and thymines into guanines and cytosines both upstream and downstream of the differentially methylated CpG more often resulted in increased methylation in SHR than in BN (Figure S11). The nucleotide bias associated in this study with increased and decreased CpG methylation showed remarkable similarity to that found previously in mouse brain [12] and in human blood [26].

### Allele-specific methylation in reciprocal F1s

**Figure 6:**
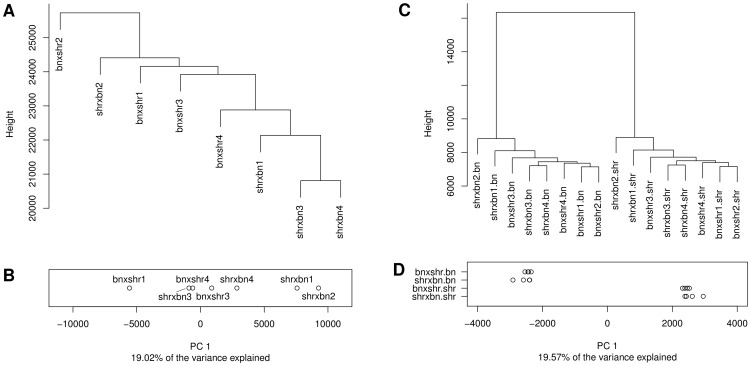
Hierarchical clustering and principal component analysis of CpG methylation profiles obtained in the F1 reciprocal crosses. Dendrograms showing the results of clustering the CpG methylation profiles obtained in each of the F1 animals before (A) and after (C) phasing the read data by parental genotype. The prefix of the phased F1 profiles in (C) denotes the reciprocal cross (bnxshr, shrxbn) and the suffix the genotype of the phased read set (bn = Brown Norway, shr = Spontaneously Hypertensive Rat). Numbers after the reciprocal cross prefix denote biological replicates. Profiles were clustered by the Ward's method using the pairwise euclidean distance between the profiles as the distance metric. Panels B and D show the projection of methylation profiles onto the 1^st^ principal component (PC). Replicates for each cross-genotype combination are separated along the y-axis. The prefix of the label denotes the reciprocal cross (bnxshr, shrxbn), the suffix denotes the parental genotype (bn, shr). While the 1^st^ PC does not separate crosses in the unphased data it provides complete separation by parental genotype in the phased data. Only CpGs with at least 5× coverage in each replicate/phased read set were included in the analysis and CpG positions affected by SNPs/indels were removed prior to clustering and principal component analysis.

To study further the relationship between allelic sequence variation and CpG dinucleotide methylation and to search for possible parent-of-origin (PO) effects, we carried out WGBS of left ventricle DNA from F1 animals from reciprocal crosses between SHR and BN parents (Table S8). After filtering, we retained and analysed approximately 25 million CpG dinucleotides (Figure S12, Figure S13). This number was greater than in the parental strains because of increased coverage in the F1 crosses compared to the parental data sets. The absence of cross specific clusters in the hierarchical clustering analysis (HCA) (Figure 6A) and the lack of a clear separation of profiles by the 1^st^ PC (Figure 6B), which captured strain specific variation in the parentals, showed that the variation in methylation profiles across animals within and between the two crosses was small. Consistent with the small variation in methylation we detected only 2,627 differentially methylated CpG dinucleotides between the reciprocal crosses (<0.01% of all analysed CpGs). This was less than one tenth of the variability seen between the two parental strains, despite the higher coverage in the F1 animals, reflecting the genetic differences between the parental strains, but the identical genomes (apart from the sex chromosomes) of all the F1 animals.

**Figure 7:**
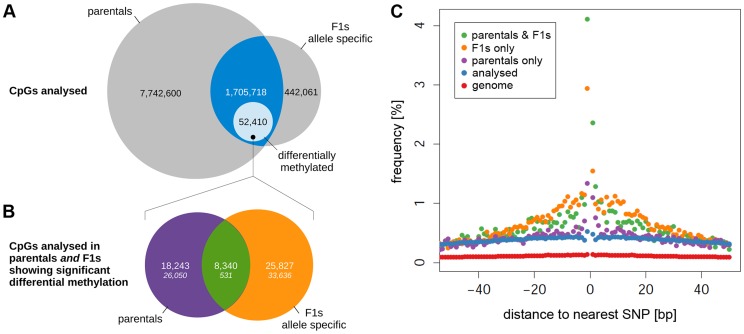
Comparison of differential methylation in parental strains and allele-specific methylation in F1 crosses. (A) Venn diagram showing the number of CpGs tested for differential methylation in the parental strains ONLY or for allele-specific methylation ONLY (grey areas) and the fraction of CpGs tested for BOTH (intersection, blue). The light blue area shows the number of CpGs significantly differentially methylated between the parentals AND/OR showing allele-specific methylation. Areas are not proportional. CpGs on the X chromosome and the mitochondrial chromosome have been excluded. (B) Overlap of differential methylation between the parental strains and allele specific methylation in the F1s. In total, 52,410 out of the 1,705,718 CpG dinucleotides analysed in both the parentals and the F1s (intersection, blue in (A)) showed significant differential methylation. The Venn diagram shows the fraction of CpGs that were differentially methylated between the parents ONLY (purple), between the alleles in the F1s ONLY (orange) or in BOTH (green). The theoretical distribution of frequencies assuming random overlap between the parental and the F1 data sets are shown in italics (*X^2^* = 135276.2, *P*<4.9×10^−324^). The overlap of the two sets is >15 fold higher than expected by chance. (C) Frequency of CpGs with a SNP within 50 base pairs for CpGs showing differential methylation between the parentals only (purple), the F1 alleles only (orange) and both (green) compared to all CpGs analysed in the parentals and the F1s (blue) and the whole genome (red).

In order to detect allele-specific methylation (ASM) in the F1 animals, SNP-containing reads were phased by parental genotype. Methylation profiles derived from the phased read data did cluster by parental genotype (Figure 6C) and were separated by genotype when projected on the 1^st^ PC (Figure 6D). The absence of cross specific sub-clusters is most likely due to the subtlety of methylation differences between the cross which is smaller than the technical variation of the method. Of the 2,170,074 CpG dinucleotides testable for ASM differences after phasing, 38,152 showed significant ASM differences (FDR<5%). Because *cis*-regulated differences in cytosine methylation should be detectable as both differential methylation in the parental strains and as ASM in the F1s, we looked for the intersection between the differentially methylated CpG dinucleotides in the parental and the F1 datasets. Of the 1,705,718 CpG dinucleotides analysed in both the parental and phased F1 data (Figure 7A, intersection), there were 8,340 autosomal CpG dinucleotides differentially methylated in an allele-specific manner in the F1s that were also differentially methylated between BN and SHR parental strains (Figure 7B, intersection). This number was 15 fold greater than expected by chance (*X* ^2^ = 137040.2, p<4.9×10^−324^). We observed the highest enrichment for proximal sequence variation amongst the subset of differentially methylated CpGs in the F1 dataset that were also found to be differentially methylated in the parental strains (Figure 7C). The increased frequency of local SNPs that we observed in this set is further evidence of enrichment for putative *cis*-regulatory sequence variants adjacent to differentially methylated CpG dinucleotides. The striking concordance of differential methylation in the parental animals and allelically-determined methylation in the F1 animals strongly suggests that the methylation level at a large number (many thousands at least) of CpG cytosines is regulated in *cis*, partly by local sequence variation. The enrichment for SNPs in the 250 bp surrounding differentially methylated CpGs, and particularly in the 10 bp window around these CpGs (Figure 7C) indicates that sequence variation in the immediate vicinity of differentially methylated CpGs underlies at least part of these differences in cytosine CpG methylation.

In addition to the finding of ASM differences in F1 animals, we also identified 723 CpG dinucleotides within 145 regions that were significantly differentially methylated (FDR<5%) in a PO-specific manner (Table S9). In 35 regions we detected a mean methylation difference between the paternal and maternal allele of more than 50%. Twenty-five of these regions have been reported previously as imprinted loci in human or mouse while 10 regions were previously undescribed (Table 2). To validate the parent-of-origin specific methylation differences we measured methylation by Fluidigm amplification and Illumina sequencing at 15 loci (four known imprinted and eleven novel) at which we detected PO-specific methylation by WGBS. The methylation differences detected by WGBS at the selected loci ranged from 20–97%. Within these 15 loci, we were able to confirm parent-of-origin dependent methylation differences for the four known imprinted loci and five of the novel loci (F1LUE4_RAT-Sgk1, 5SrRNA-Pmfbp1, Col9a2, Mthfd2l, Region ID #97; Table S10). Validation for one of the loci was only achieved on the BN parental chromosome, most likely due to insertion deletion polymorphisms on the SHR chromosome which led to the absence of sequencing reads from the SHR chromosome at this locus.

**Table 2:** Parent-of-origin specific methylation differences^a^ detected in the F1 crosses.

#	Chr	Region Size [bp]	CpGs in Region	Max. Mean Methylation Difference [%]	Allele Showing Increased Methylation^b^	Locus	PubMed ID
**Known imprinted** c							
1	1	306	30	98	M	Plagl1	10655556
27	1	318	12	61	P	H19/Mir675	11861904
3	1	278	16	94	P	H19/Mir675	11861904
9	1	94	7	85	P	H19/Mir675	11861904
6	1	847	34	91	P	H19/Mir675	11861904
45	1	290	4	50	P	Igf2	7607083
25	3	369	15	64	M	L3mbtl	15123827
16	3	138	11	69	M	Zfp64-Zfp217	21705755
2	3	475	34	97	P	Gnas	10097123
43	6	166	8	51	P	Meg3/Gtl2-Dlk1	12682775
41	6	12	2	52	P	Meg3/Gtl2-Dlk1	12682775
10	6	401	15	80	P	Meg3/Gtl2-Dlk1	12682775
5	7	1,510	74	92	M	Trappc9	20616232
8	7	8	2	86	M	Trappc9	20616232
17	8	1	1	68	P	Rasgrf1	16314537
33	8	8	3	57	M	Rasgrf1	16314537
13	8	153	7	72	P	Rasgrf1	16314537
21	8	282	8	66	P	Rasgrf1	16314537
19	9	383	8	67	P	Gpr1/Zdbf2	19200453
11	9	95	5	76	P	Gpr1/Zdbf2	19200453
22	9	471	9	65	P	Gpr1/Zdbf2	19200453
12	9	859	17	73	P	Gpr1/Zdbf2	19200453
28	9	580	15	59	P	Gpr1/Zdbf2	19200453
4	14	331	39	93	M	Grb10	10861285
15	14	1	1	69	M	Commd1/Murr1	14673161
**Novel**							
44	1	93	12	50	M	F1LUE4_RAT-Sgk1	
14	2	1	1	70	P	Dclk1	
20	3	1	1	66	M	Fbxo3-Hipk3	
35	3	1	1	55	P	LOC100361388-Sptlc3	
7	7	1	1	86	P	Pdzrn4-Glt8d3	
18	8	1	1	68	M	Snora17	
29	13	1	1	58	M	Lamc1	
31	17	1	1	57	P	Barx1	
42	19	3	2	52	M	5SrRNA-Pmfbp1	
23	20	1	1	64	P	RT1-Bb-RT1DOb	

a Statistically significant differences after multiple testing correction (FDR<5%).

b (M) maternally derived allele or (P) paternally derived allele showing increased methylation.

c Known imprinted: where a locus has previously been reported as imprinted, the PubMed ID of the respective publication is given.

**Figure 8:**
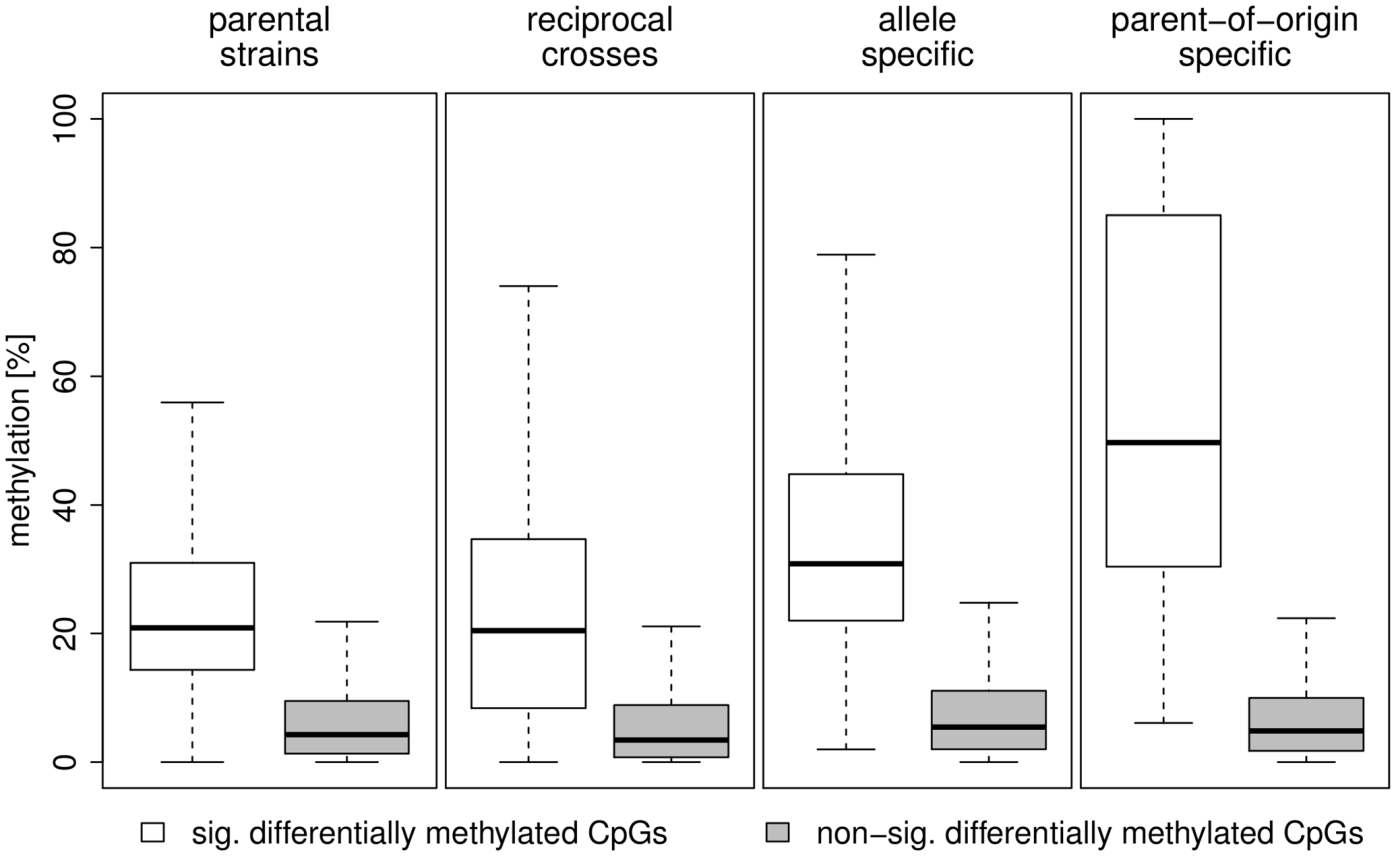
Distribution of methylation differences by source of variation. The boxplots show the the distribution of methylation differences for significantly differentially methylated CpGs (white) and non-significantly differentially methylated CpGs (grey) between the parental strains, the F1 reciprocal crosses, allele specific methylation and parent-of-origin dependent methylation.

Table 3 and Figure 8 summarise methylation differences we detected in this study by source of variation. Table 3 shows the proportion of CpGs that were differentially methylated in the analysis of i) inter-strain differences in methylation, ii) differential methylation between reciprocal crosses, iii) allele specific methylation and iv) parent-of-origin dependent methylation. Figure 8 shows the distribution of methylation differences in the respective analyses. CpGs methylated in a parent-of-origin dependent manner exhibited the highest median difference in methylation followed by CpGs that showed allele-specific methylation. The small number of differentially methylated CpGs detected between the F1 reciprocal crosses had a median methylation difference similar to that of CpGs differentially methylated between the parental strains albeit with a broader range of differences.

**Table 3:** Summary of differential methylation by source of variation.

	Number of CpGs analysed*	Number of CpGs differentially methylated*	Percentage of CpGs differentially methylated
**Parental strains**	10,614,445	77,088	0.73%
**F1 reciprocal crosses**	24,887,377	2,627	0.01%
**Allele specific**	2,170,074	38,152	1.76%
**Parent-of-origin specific**	2,170,074	723	0.03%

*Strand specific methylation.

## Discussion

Cytosine methylation at CpG dinucleotides is amongst the most studied epigenetic marks, yet at present there is little information about the extent to which inter-individual differences in CpG methylation in adult tissues are genetically determined by germline DNA sequence variation and how inter-individual variation in methylation relate to whole-body phenotypes. Previous studies in humans and experimental animals have shown evidence of individual or allele-specific differences in methylation at CpG cytosines [4]–[12]. However, these have either assayed methylation at a small fraction of genomic loci, used low resolution techniques, not taken account of SNPs that disrupt CpG sites, studied single or small numbers of individuals, or only indirectly inferred the difference between *cis* and *trans* regulation of CpG methylation.

We applied WGBS to assay CpG methylation at single-nucleotide resolution across the genome in multiple isogenic animals, and carried out segregation studies in F1s and linkage studies in RI lines to define the extent and mode of inheritance of inter-individual variation in CpG cytosines in the adult rat heart. While methylation profiling has been carried out previously in the rat [27] this is the first genome wide study at single nucleotide resolution methylation to look at the association of genotype, epigenotype and phenotype in an animal model of human disease. Methylation studies in human heart tissue have used Illumina BeadChip arrays [28] and MeDIP-seq [29] to examine cytosine methylation in cardiomyopathy (CM). However, these studies did not account for inter-individual germline differences in the genome sequence which as we show here is an important determinant of inter-individual variation in DNA methylation and may in part explain the methylation differences observed in CM patients.

We measured cytosine methylation at over 10 million CpG sites across the genome, showed that inter-strain variability in cytosine methylation is markedly greater than intra-strain variability and detected differential methylation between the strains at over 75,000 CpG cytosines, amounting to 0.7% of all analysed CpG sites. Our studies in animals from parental strains, F1s and RI lines show that inter-strain differences in CpG methylation are substantially *cis*-regulated, essentially under control of sequence variation at a single locus in *cis* defined as the extent of the linkage region and the resolution of the genetic map which has an average distance between genetic markers of ∼2.7 MB. Furthermore, we were able to demonstrate that the detected methylation differences are largely independent of differences in tissue composition between the two strains.

Our Gene Ontology analysis showed that genes that were closest to differentially methylated regions, defined as a minimum of five adjacent differentially methylated CpGs, were strongly enriched for gene products localising to the plasma membrane, or involved with neuron differentiation or cell communication. Interestingly, a study of genetic variation in the SHR rat found that genes showing major variation in their coding region between SHR and BN also showed enrichment for genes encoding plasma membrane components and neurological processes [16]. These data suggest that, in addition to direct *cis* effects on methylation, differential methylation near genes associated with the enriched GO categories could be the result of genetic variation in genes with similar biological function that act indirectly through feedback mechanisms at the molecular or organismal level. Although potentially of interest, such observations would require more detailed investigation to confirm and understand these associations. Furthermore, we find significant correlation between CpG methylation and serum CgB levels. Serum CgB levels are a correlate of sympathetic nervous system overactivity and have been proposed as a biomarker for heart failure [30]. These findings suggest a link between genetic, epigenetic and disease-associated phenotypes the causal relationship of which remains to be determined.

Analysis of genomic sequence showed that the 250 bp surrounding differentially methylated CpGs are enriched for sequence variants between the SHR and BN strains. We found biases in nucleotide usage in the 5 bp immediately adjacent to differentially methylated CpGs that are associated with increased or decreased methylation. Remarkably, the nucleotide signatures that we identified are almost identical to those recently identified as being associated with increased and decreased methylation in mouse brain [12] indicating near complete conservation across tissues and species. Nearly identical signatures were also associated with increased and decreased methylation in a separate analysis of CpG methylation associated with ageing in human blood [26]. This extent of conservation across tissues and species suggests a common mechanism contributing to regulation of CpG methylation that merits investigation in future studies.

Further to our findings of differential methylation in the parental strains, we identified over 38,000 CpG dinucleotides that showed allele-specific methylation in F1 animals. This finding of allele-specific methylation in F1 animals is most likely due to *cis*-regulatory mechanisms and is consistent with previous studies in humans and mice reporting *cis*-regulation of CpG methylation [4]–[10], [12], [31]. Our data define a genome-wide set of CpGs that are susceptible to allele-specific regulation of CpG methylation in the mammalian heart. Since in our study allele-specific methylation could only be detected in F1 animals when sequence reads could be phased by parental genotype, we could only seek the presence of allele-specific methylation in 2.1 million of the total of more than 25 million CpG dinucleotides in the rat genome. Accordingly it is likely that the number of CpG dinucleotides influenced by *cis*-regulated methylation is up to 10-fold greater than the 38,000 detected in this study in the genomes of these two strains.

Our studies in F1 animals also detected parent-of-origin (PO) effects on DNA methylation with 723 CpG dinucleotides clustered in 145 regions showing evidence of PO-type regulation of methylation. To our knowledge, this is the first empirically-based prediction of imprinted genes in the rat. Thirty-five of these regions showed CpG methylation differences of more than 50% between paternally and maternally-inherited alleles. Of these, 25 have been previously reported as imprinted loci in human and mouse, including 19 identified recently in brain [12] while 10 regions were previously undescribed.

The detected methylation differences ranged from a ∼20% median difference in the parental strains to ∼50% median difference for parent-of-origin specific methylation. The continuous distribution of methylation differences and the practical absence of mono-allelic methylation (i.e. 100% methylation on one allele 0% methylation on the other) is probably explained by i) the methylation measurements being composite signals coming from a mix of different cell types present in the left ventricle and ii) the stochastic nature of methylation reaction.

Our mapping studies in the BXH/HXB RI strains showed that, treated as a quantitative trait, methylation at 151 of 212 CpGs, residing within 36 amplicons, showed *cis* linkage with LOD scores of 3.9–32.0. Most of these showed complete segregation of CpG methylation with genotype indicating essentially single-locus control in *cis*. Methylation of six CpGs in the *Odfp2* amplicon mapped in *trans* (LOD scores 3.6–7.6), with the *trans*-regulating meth-QTL locus residing on a different chromosome to the regulated CpGs. The fact that over 95% of the meth-QTLs for individual CpGs that showed linkage in the RI strains were *cis*-regulated is in keeping with our nucleotide preference data in the parental strains, our *cis/trans* tests in the F1s, and with previous data [11], [12] that correlate local sequence variation with CpG methylation status. The explanation for the two loci that showed *trans*-regulated methylation is less clear. For *Odfp2*, there were no candidate genes in the 2-LOD confidence interval of the *trans* meth-QTL that had GO annotations related to DNA methylation, chromatin status or DNA binding, suggesting either a novel mechanism for regulation of DNA methylation, or possibly a role for one of the several non-coding RNAs in this interval. For *Asap2*, the large number of genes in the QTL interval precludes assessment of candidates without further investigation. The finding that methylation is predominantly regulated in *cis* complements the analysis of SNP frequency adjacent to differentially methylated CpGs. Both results point to the importance of *cis* regulation although the underlying mechanisms and relative contribution of proximal and distal sequence effects require further investigation. Whilst a previous analysis has suggested that most *cis*-acting allelic influences on CpG methylation may be up to 149 kb [9], our study and that of Gibbs et al [6] detect allelic associations over much shorter distances of 1–45 bp from differentially methylated CpGs. The observed co-segregation of genotype and differential methylation is consistent with the results of a recent study of inbred lines in plants [13], suggesting that *cis*-regulated control of inter-individual variation in cytosine methylation is strongly conserved across species and between animals and plants.

Many previous studies of CpG methylation have been carried out in cell lines or homogeneous cell types isolated in primary cultures, while we used whole cardiac tissue for these studies. The spontaneously hypertensive rat is a model of cardiovascular disease that has been extensively characterised genetically and phenotypically for hypertension, cardiac hypertrophy and failure and insulin resistance [18]–[20], [22], [32]–[34] but the role of the cardiac methylome in these phenotypes remains unexplored. It might be expected that the study of whole tissue harvested *ex vivo* might reduce our ability to detect genetic effects because of cell type heterogeneity, possible paracrine effects or secondary effects of whole-body phenotypes such as hypertension. Despite this, we find consistent genetic effects on CpG methylation in cardiac tissue from parental strains, F1 animals and RI lines derived from the two parental strains. In particular, the finding that the CpG sites showing allele-specific methylation in F1 animals were enriched 15-fold (p<10^−324^) for CpG sites also showing differences in CpG methylation in the parental strains is evidence of the stability and robustness of these genetic data.

Previous studies of gene expression showed that the heritability of gene expression in heterogeneous compared to homogeneous cell types is dominated by *cis*-regulated gene expression [35], and also that expression differences that are found in single cell populations may not be found in heterogeneous tissues [36]. By analogy, it is possible that genetic analysis of DNA methylation in more homogeneous cell populations than were studied here would detect stronger *cis* effects and more extensive *trans* effects on CpG methylation than were detected in our studies of intact heart tissue. Notwithstanding, our study was able to detect strong allelic effects on CpG cytosine methylation and, in addition, our analysis of methylation differences in isolated cardiomyocytes and non-cardiomyocytes showed that for the large majority (>75%) of CpGs analysed in this study, differential CpG methylation in the heart tissue was independent of cell type. Taken together, these studies define a minimum extent of genetic regulation of CpG methylation across these two rat strains in the rat heart.

## Materials and Methods

### Strains and tissue processing

Rats were housed in an air-conditioned animal facility and allowed free access to standard laboratory chow and water. All experiments were done in agreement with the Animal Protection Law of the Czech Republic and were approved by the Ethics Committee of the Institute of Physiology, Czech Academy of Sciences, Prague. We collected left ventricular tissue from unfasted males from six week old male BN-Lx/Cub (referred to in this study as BN), SHR/Olalpcv (referred to as SHR) (n = 4 per strain); and from (BNxSHR)F1 and (SHRxBN)F1 (n = 4 per cross); and from male rats from 29 BXH/HXB recombinant inbred strains (n = 2 per strain) [23] between 9:00am and 10:00am. Left ventricles were snap frozen in liquid nitrogen and stored at −80°C. The BN-Lx/Cub and SHR/Olalpcv lines have been inbred over more than 80 generations [17]. For tissue heterogeneity studies, cardiomyocytes and non-cardiomyocytes were isolated as previously described [37] from SHR/Ncrl and BN.NCrl rats (four biological replicates each) and studied alongside whole heart tissue from the same strains, as well as left ventricle tissue from SHR/Olalpcv and BN-Lx/Cub rats (three biological replicates each). No differences were found between the SHR/Olalpcv and BN-Lx/Cub and the respective NCrl strains which are also referred to as SHR or BN in the analysis of tissue heterogeneity.

### Whole genome bisulfite sequencing (WGBS)

Frozen tissues from BN rats, SHR rats and the reciprocal F1 animals were processed without pooling to generate methylation profiles on the HiSeq 2000 platform (Illumina) as described in [38].

### Analysis of global DNA methylation levels using Luminometric Methylation Assay (LUMA)

A luminometric-based assay for global DNA methylation was performed according to the protocol described in [39]. Briefly, 250 ng of genomic DNA extracted from liver (n = 3), kidney (n = 3) and left ventricle (n = 4) of BN and SHR animals was subjected to double-digestion with *EcoRI* and methylation-sensitive *HpaII* and methylation insensitive *MspI* restriction endonucleases and global methylation between strains quantified by pyrosequencing.

### RNA-seq library preparation and data collection

RNA was extracted from 25 mg of crushed left ventricular tissue from four BN and four SHR animals without pooling, using Trizol (Invitrogen) according to manufacturer's instructions. 4 µg of total RNA was used to generate RNA-seq libraries using TruSeq RNA kit (Illumina) according to manufacturer's instructions. Libraries were multiplexed in pairs and run on a single lane of the HiSeq 2000 platform (Illumina) to generate 100 bp paired-end reads.

### Amplification and analysis of CpG dinucleotide methylation using Sanger sequencing

500 ng of genomic DNA was bisulfite converted using the MethylCode Bisulfite Conversion Kit (Invitrogen) according to manufacturer's instructions. Diluted by 1∶8, bisulfite-converted DNA was amplified using PFU Turbo Cx polymerase with primers designed with the Meth primer program (http://www.urogene.org/methprimer/index1.html). PCR products were purified using the MultiScreen PCR_μ96_ plate (Millipore). Sanger-sequenced CpG dinucleotides were scored in the Sequencher 5.0 analysis software (Gene Codes Corporation). A secondary peak was called if it was ≥5% of the primary peak height. The peak height ratio was used to calculate percentage methylation at CpG sites. Primer sequences are available on request. To ensure that we mapped CpG dinucleotide methylation rather than methylation of CpG disrupting SNPs (e.g. CG>TG), we carried out genomic DNA sequencing in BN and SHR for all 14 regions containing the differentially methylated CpG dinucleotides and only mapped CpG dinucleotide methylation where the CpG dinucleotide was present in both strains.

### Amplification and analysis of CpG dinucleotide methylation using Fluidigm and Illumina sequencing

500 ng of genomic DNA was bisulfite converted using the MethylCode Bisulfite Conversion Kit (Invitrogen) according to manufacturer's instructions. 50 ng of bisulfite-converted DNA and target regions were PCR amplified utilising the Fluidigm 48.48 Access Array. Primers were designed with the Sequenom primer program (http://www.epidesigner.com) and FastStart High Fidelity PCR system (Roche) was used for amplification. PCR amplification was performed for 40 cycles with an annealing temperature of 57°C. Primer sequences are available on request. PCR products from each sample were indexed, pooled and purified using the Agencourt AMPure XP system (Beckman Coulter). Sequencing libraries were prepared from the pooled samples and sequenced on the Illumina MiSeq platform. Demultiplexed sequencing reads were mapped and methylation profiles generated as described in [38]. Differentially methylated CpG dinucleotides were analysed for the presence of a disrupting SNP or INDEL and only CpG dinucleotide methylation where the CpG dinucleotide was present in both strains was mapped.

### Rat genome reference sequence

The rat genome assembly RGSC3.4 (rn4) was used as the reference sequence. Reference sequence files in FASTA format were obtained from the UCSC genome browser website (ftp://hgdownload.cse.ucsc.edu/goldenPath/rn4/chromosomes).

### Genome annotation

Transcription start site, exon and intron annotations for the rat genome assembly RGSC3.4 were obtained from the Ensembl database (http://www.ensembl.org) version 62. CpG island coordinates (cpgIslandGgfAndyMasked) were obtained from the UCSC genome browser (http://genome.ucsc.edu).

### BN-SHR sequence variation

We used the BN-Lx/Cub and SHR/OlaIpcv genome sequence variation data previously reported [17], using the SNV and indel caller described in [17].

### WGBS read mapping and processing

WGBS read mapping and processing was carried out as described in detail in [38]. In brief, WGBS reads were aligned against the rat genome reference sequence with BWA [40] version 0.5.8a. Prior to alignment, reads were pre-processed as follows: i) the conversion state of read pairs was masked by converting all C base calls of read1 to Ts and all G base calls of read2 to As ii) the first read base was clipped to avoid false negative methylation calls from unmethylated cytosines introduced at the fragment 5′ end during the end-repair step of library preparation iii) reads were quality trimmed at the 3′ end with the -q option of BWA using a Q score cutoff of 20.

To map reads originating from the bisulfite converted forward strand of the genomic DNA, reads were aligned to an *in silico* bisulfite-converted version of the reference sequence with all Cs converted to Ts; to map reads originating from the bisulfite converted reverse strand reads were aligned to the G-to-A converted reference sequence. To reduce allele-specific mapping bias, reads generated in SHR samples were mapped to a reference sequence that was converted to the SHR allele at BN-SHR SNP positions prior to C-to-T/G-to-A conversion. Reads generated in F1 animals and thus of unknown haplotype at BN-SHR SNP positions were mapped against a reference sequence with all SNP positions masked by replacing them with N. Spiked-in unmethylated lambda phage control DNA was mapped to the lambda reference sequence (Genbank accession NC_001416.1) which was *in silico* bisulfite converted in the same way as the rat reference genome sequence.

After alignment, the 3′ ends of overlapping read pairs were clipped retaining the higher quality end in order to avoid duplicate methylation calls in the overlap region. Subsequently read mappings were filtered removing i) clonal reads ii) reads with a mapping quality <20 iii) read pairs mapping to both the *in silico* converted forward and the *in silico* converted reverse strand iv) reads with invalid mapping orientation.

### Depth of coverage analysis

Depth of coverage statistics for the filtered WGBS mappings were calculated with the DepthOfCoverage command of the Genome Analysis Toolkit [41] version 1.1.23.

### Methylation calling, differential methylation analysis and methylation profile filtering

Mapped, processed and filtered reads were piled up with the samtools [42] version 0.1.16 mpileup command and the number of cytosine and thymine base calls counted at each cytosine position. The relative methylation level at each cytosine position was calculated as the percentage of cytosine base calls of the total number of cytosine and thymine base calls. Methylation calls were corrected for incomplete bisulfite conversion by subtracting the average number of expected unconverted cytosines at the depth of coverage at the respective position given the bisulfite conversion rate for the respective sample prior to calculating relative methylation levels. For WGBS libraries generated from F1 samples the bisulfite conversion rate was calculated from the frequency of unconverted cytosines in the unmethylated lambda control spike-ins. For WGBS libraries generated from BN and SHR samples, the bisulfite conversion rate was estimated by regression analysis of non-CpG conversion rate and lambda conversion rate in the F1 samples. Bisulfite conversion rates for BN and SHR samples were then predicted from the non-CpG conversion rate in these samples. Bisulfite conversion rates were >97% for all samples.

Differential methylation at cytosines was tested for by Fisher's exact test on a 2×2 contingency table testing for independence of strain/cross/allele and the frequency of unconverted and converted cytosines across all replicates. P-values were adjusted for multiple testing using the false discovery rate (FDR) method by Benjamini and Hochberg [43].

Methylation profiles were filtered prior to multiple testing correction i) Retaining only cytosines with a minimum combined read coverage of 5× across a minimum of three replicates. ii) Removing CpN dinucleotides affected by BN-SHR sequence variation (SNP and indels) to exclude methylation differences resulting from the disruption or deletion of methylation sites.

### Hierarchical clustering and principal component analysis of CpG methylation profiles

Methylation profiles of CpG dinucleotides with at least 5× coverage in each of the replicates/phased data sets were clustered based on the pairwise euclidean distance between the vectors of methylation levels for each animal scaled down to 5× coverage. CpG dinucleotides affected by BN-SHR sequence variation (SNPs and indels) were removed from the data set prior to the analysis. Profiles were subsequently clustered using Ward's minimum variance method [44]. Distance calculations and clustering were carried out with the statistical software package R [45] using the dist and hclust functions, respectively. Principal component analysis was carried out with the pca function of the pcaMethods R package.

### Meth-QTL and physiological QTL mapping in the RI strains

From Sanger and Illumina sequencing of PCR products, we derived mean methylation values from the two biological replicates for each RI strain. We carried out genome-wide linkage analysis in the 29 BXH/HXB RI strains for all 212 CpG dinucleotides and the average methylation percentage of all CpG within each of the 40 PCR amplicons. Linkage analysis was carried out as previously described [14] using QTL Reaper (K.F. Manly; University of Tennessee Health Science Center, Memphis, Tennessee) except that a denser SNP map of 1384 markers was used [46]. To account for non-normally distributed data, the empirical significance of the meth-QTLs was assessed by 1 million permutations [47]. The same genome wide correction was applied for testing both *cis* and *trans* linkage. We defined regions as regulated in *cis* or in *trans* by defining *cis* when the peak of linkage marker lay within 5 Mbp of the genomic location of the amplicon, with other linkages being defined as *trans*. Candidate genes and Gene Ontologies for the *Odfp2* and *Asap2* meth-QTL interval were taken from Ensembl release 66.

### Principal component analysis of methylation measurements in cardiomyocytes and non-cardiomyocytes

Principal component analysis of methylation measurements in cardiomyocytes and non-cardiomyocyte cell populations isolated from BN and SHR cardiac tissue was carried out using the pca function of the pcaMethods R package with unit variance scaling.

### Quantitative trait methylation (QTM) analysis

Measurements of 241 phenotypic traits measured across the RI strain panel (Table S11) were provided by Michal Pravenec. Outliers were removed from the raw measurements within each strain using boxplot analysis, grubb test and Nalimov test. After outlier removal mean and standard error where calculated for each trait and strain. Association of average locus methylation in the RI strains and physiological phenotypes was then assessed by Pearson's correlation test. P-values were adjusted for multiple testing using the false discovery rate (FDR) method by Benjamini and Hochberg [43].

### RNA-seq read mapping and differential expression analysis

RNA-seq reads were aligned to the rat genome reference sequence assembly RGSC3.4 (rn4) with TopHat [40] version 1.3.0. To reduce allele-specific mapping bias, reads generated in SHR samples were mapped to a reference sequence that was converted to the SHR allele at BN-SHR SNP positions.

Exon expression was quantified by counting read-pairs mapped to exon locations annotated in Ensembl version 62 using the NxtGenUtils (http://code.google.com/p/nxtgen-utils) version 0.12 CountReads command. Differential expression analysis was carried out with DESeq [48] version 1.6.1 on the raw exon fragment counts. The analysis was run with the default parameters except for the estimateDispersions function which was run with the ‘pooled’ option set to true. An FDR-adjusted P-value<0.05 was chosen as the significance threshold for differential expression.

### Analysis of SNP allele frequencies at differentially methylated CpG dinucleotides

SNP allele bias for alleles at differentially methylated CpGs was examined by calculating the information content [49] for the observed SNP allele frequencies at the five base pairs up- and downstream of CpGs showing increased or decreased methylation in the SHR and BN strains. Calculations and visualisation were carried out with the Bioconductor seqLogo package implemented in R. The seqLogo code was modified to take into account nucleotide usage in the rat genome (29% A, 29% T, 21% C, 21% G). To compare nucleotide usage around hyper- and hypomethylated CpGs in rat and mouse nucleotide usage information was obtained as personal communication from the authors of [Xie et al., 2012]. Position frequency matrices derived from rat and mouse nucleotide usage data were compared with the TOMTOM tool of the MEME suite version 4.9 [25].

### Phasing of WGBS reads by parental genotype

WGBS read pairs generated in the reciprocal F1 crosses that mapped to BN-SHR SNP positions were phased by parental genotype by determining the SNP allele in the read sequences using the NxtGenUtils (http://code.google.com/p/nxtgen-utils) version 0.12 PhaseRead command. C/T SNP positions were not used for phasing because of the difficulty of distinguishing allelic variation from bisulfite conversion at these positions in WGBS reads. Read pairs with ambiguous allele patterns (equal frequency of BN and SHR SNP alleles) were discarded.

### Detection of allele and parent-of-origin specific methylation differences

Allele and parent-of-origin specific differences in methylation were detected by carrying out differential methylation analysis on the phased F1 WGBS read data as described above for the parental and unphased F1 data.

Allele-specific methylation differences were determined separately for maternally and paternally derived chromosomes, i.e. methylation on the (maternally-derived) BN allele in the (BNxSHR)F1 cross was compared to methylation on the (maternally derived) SHR allele in the (SHRxBN)F1 cross. Methylation on the (paternally-derived) BN allele in the (SHRxBN)F1 cross was compared to methylation on the (paternally-derived) SHR allele in the (BNxSHR)F1 cross. A CpG was reported as allele-specifically methylated if it showed a statistically significant difference (as above) in methylation in either or both the comparisons.

By analogy, parent-of-origin specific methylation differences were determined by comparing methylation on the maternal and paternal allele separately for the BN and SHR derived chromosomes. Only differentially methylated regions that show a consistent direction of methylation differences in all comparisons were reported.

### Clustering of differentially methylated cytosines into differentially methylated regions

Differentially methylated cytosines were clustered into differentially methylated regions by grouping differentially methylated cytosines not further away than 500 bp from each other irrespective of the direction of the methylation difference using BEDTools [50].

### Gene Ontology analysis

Genes whose gene body (exons and introns) or the region 5000 bp upstream or downstream overlapped with differentially methylated regions containing five or more differentially methylated CpGs showing the same direction of methylation differences were tested for enrichment of Gene Ontology terms. As background the set of all genes whose gene body +/−5000 bp overlapped with CpGs tested for differential methylation was used. The Gene Ontology analysis was carried out with DAVID 6.7 [51], [52] using default settings. Enriched Gene Ontology terms were subsequently clustered by similarity with REVIGO [53] setting the output option for ‘list size’ to ‘small’. Otherwise default settings were used.

## Supporting Information

Figure S1 Depth of coverage of quality-filtered whole genome bisulfite sequencing data generated in the parental strains. (A) Cumulative coverage across the genome. The plot shows the percentage of bases (ungapped sequence of the RGSC3.4 reference assembly) covered by at least *x* reads. (B) Cumulative coverage of cytosines in CpG context (CpGs on forward and reverse strand were counted separately) (C) Cumulative coverage of cytosines in CpG context with coverage in at least 3 replicates in both strains (CpGs on forward and reverse strand were counted separately). The dotted line marks the minimum coverage cut off for CpGs included in the differential methylation analysis.
(PDF)

Figure S2 Analysis of global DNA methylation levels by Luminometric Methylation Assay (LUMA). Genomic DNA extracted from liver, kidney and left ventricle (LV) of BN and SHR animals was subjected to double-digestion with *EcoRI* and methylation-sensitive *HpaII* and methylation insensitive *MspI* restriction endonucleases and global methylation quantified by pyrosequencing. Unmeth = unmethylated control DNA, meth = fully methylated control DNA, n = replicates. The error bars indicate the standard deviation.
(PDF)

Figure S3 Clusters of differentially methylated CpG dinucleotides. (A) Frequency plot showing the size distribution of clusters of differentially methylated CpG dinucleotides (black bars, left-hand y-axis) obtained when grouping adjacent differentially methylated CpG dinucleotides not further than 500 bp away from each other. Singletons (29,313 differentially methylated CpG dinucleotides) are excluded from the plot. The red line shows the cumulative frequency of differentially methylated CpG dinucleotides contained within the clusters (right hand y-axis). (B) Frequency plot showing the number of differentially methylated CpG dinucleotides contained in the clusters of differentially methylated CpGs.
(PDF)

Figure S4 Distribution of methylation levels at CpG cytosines analysed for differential methylation between BN and SHR strains. The x-axis shows the average percent methylation across replicates and the y-axis the number of CpG di-nucleotides.
(PDF)

Figure S5 Distribution of methylation differences across genomic regions. (A) Proportion of differentially methylated CpGs within different genomic regions compared to the rest of the genome for CpGs inside CpG islands (TSS = transcription start site as annotated in Ensembl version 62; TSS 500 = region 500 bp upstream and downstream of TSS; TSS 1000 = region 1000 bp upstream and downstream of TSS). (B) Proportion of differentially methylated CpGs within different genomic regions compared to the rest of the genome for CpGs outside CpG islands.
(PDF)

Figure S6 Scatter plots of methylation percentages obtained by Sanger sequencing or Fluidigm and whole genome bisulfite sequencing (WGBS) in the SHR and BN inbred strains. (A) Methylation data were obtained by Sanger sequencing (y-axis) and WGBS (x-axis) for 70 individual CpG dinucleotides in each of the Brown Norway (n = 4) and the spontaneously hypertensive rat (n = 4) strains. (B) Methylation data were obtained by Fluidigm (y-axis) and WGBS (x-axis) for 131 individual CpG dinucleotides in each of the Brown Norway (n = 4) and the spontaneously hypertensive rat (n = 4) strains. Pearson's correlation coefficent and associated p value are given.
(PDF)

Figure S7 LOD plots of 20 meth-QTLs. The average methylation percentage across amplicons *Akr1b10*, *Tbc1d30*, *Bl1_RAT* and *Rtdr1* did not show significant linkage. For *Tbc1d30* the individual CpG dinucleotides mapped in *cis*. LOD scores for linkage of average amplicon methylation levels are shown in red, LOD scores for linkage of individual CpG dinucleotides are shown in grey. The peak of linkage is indicated by a dashed, blue vertical line.
(PDF)

Figure S8 Methylation of individual CpG dinucleotides across the Epha2 amplicon measured by Sanger sequencing of PCR-amplified bisulfite-treated DNA. Seven CpG dinucleotides were assayed by Sanger sequencing with three (*) showing significant differential methylation (p<0.05) between the Brown Norway (BN) and the Spontaneously Hypertensive Rat (SHR). CpG dinucleotide 160,184,634 shows an opposing allelic effect as methylation is decreased in the BN strain whereas at CpG dinucleotides 160,184,557 and 160,184,560 methylation is increased in the BN strain compared to SHR.
(PDF)

Figure S9 Methylation differences between BN and SHR across different cell- and tissue-samples. Shown are average methylation levels across each amplicon in cardiomyocyte preparations (m), non-cardiomyoctye preparations (nm), whole heart samples (wh) and left ventricle samples (lv). Amplicon length and number of measured CpGs are given in brackets behind the amplicon name. The significance of a two-way ANOVA testing for independence of CpG methylation levels and the two factors strain and tissue are shown below the amplicon name.
(PDF)

Figure S10 Distance to the nearest SNP and extent of differential methylation. Histograms showing the distribution of methylation differences between BN and SHR for CpGs at different distances from the nearest SNP.
(PDF)

Figure S11 Distribution of methylation differences between the parental strains depending on the alleles of SNPs directly adjacent to the CpG dinucleotide. (A) Boxplot of the distribution of methylation differences for BN/SHR allele changes at the nucleotide directly upstream of the CpG dinucleotide that, based on the motif analysis, are predicted (i) to result in decreased methylation in SHR (red), (ii) to result in increased methylation in SHR (blue) or (iii) to have a neutral effect on methylation (green). The height of the box indicates the frequency of each allele change. The total observation frequency for each group of changes (increased, decreased, neutral) is shown by the bar plot on the right. (B) Boxplot of the distribution of methylation differences for BN/SHR allele changes at the nucleotide directly downstream of the CpG dinucleotide.
(PDF)

Figure S12 Depth of coverage of quality-filtered whole genome bisulfite sequencing data generated in the reciprocal F1 crosses. (A) Cumulative coverage across the genome. The plot shows the percentage of bases (ungapped sequence of the RGSC3.4 reference assembly) covered by at least *x* reads. (B) Cumulative coverage of cytosines in CpG context (CpGs on forward and reverse strand were counted separately) (C) Cumulative coverage of cytosines in CpG context with coverage in at least 3 replicates in both crosses (CpGs on forward and reverse strand were counted separately). The dotted line marks the minimum coverage cut-off for CpGs included in the differential methylation analysis.
(PDF)

Figure S13 Differential CpG methylation between the reciprocal F1 crosses and allele-specific CpG methylation. (A) Distribution of relative methylation levels at cytosines analysed for differential methylation between the reciprocal F1 crosses. (B) Distribution of relative methylation levels at cytosines analysed for allele-specific methylation in the F1 crosses. The histograms show the distribution of methylation levels measured for the BN and the SHR allele.
(PDF)

Table S1 Mapping and filtering statistics for whole genome bisulfite sequencing reads generated in BN and SHR rats.
(PDF)

Table S2 Gene Ontology analysis of genes associated with differentially methylated regions.
(PDF)

Table S3 CpG dinucleotides tested for linkage in recombinant inbred strains.
(PDF)

Table S4 *Trans* meth-QTL interval genes.
(PDF)

Table S5 Read mapping statistics for RNA-seq reads generated in BN and SHR rats.
(PDF)

Table S6 Summary of the results of a two-way ANOVA to test for independence of methylation levels and the two factors strain and cell/tissue type.
(PDF)

Table S7 Comparison of position frequency matrices of nucleotides associated with hyper- and hypomethylated CpGs in rat and mouse.
(PDF)

Table S8 Mapping and filtering of whole genome bisulfite sequencing reads generated in reciprocal F1 crosses.
(PDF)

Table S9 Summary of genomic regions showing parent-of-origin specific differences in methylation.
(PDF)

Table S10 Summary of validation of parent-of-origin specific differences in methylation by targeted bisulfite-seq.
(PDF)

Table S11 Phenotypes tested for correlation with methylation measurements across the RI strain panel.
(PDF)
