# Supplementary figures and images for: Genetic Analysis of the Cardiac Methylome at Single Nucleotide Resolution in a Model of Human Cardiovascular Disease
Source: PLoS Genet. 2014 Dec 4;10(12):e1004813. doi: 10.1371/journal.pgen.1004813 (PMC4256262; doi:10.1371/journal.pgen.1004813)

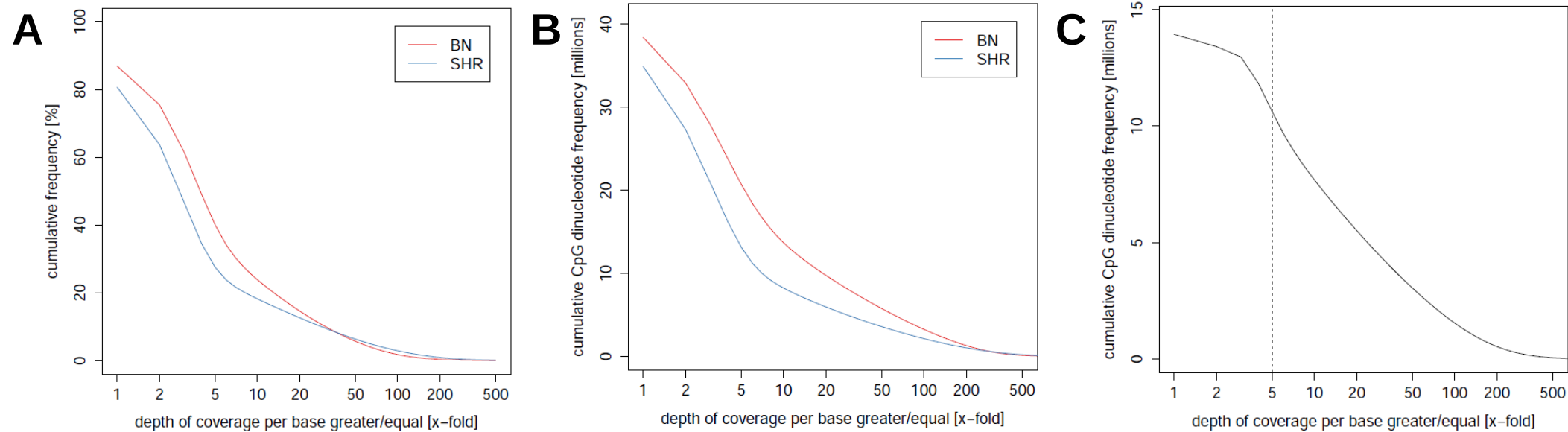

Figure S1

Supplement: Figure S1 — Depth of coverage of quality-filtered whole genome bisulfite sequencing data generated in the parental strains. (A) Cumulative coverage across the genome. The plot shows the percentage of bases (ungapped sequence of the RGSC3.4 reference assembly) covered by at least x reads. (B) Cumulative coverage of cytosines in CpG context (CpGs on forward and reverse strand were counted separately) (C) Cumulative coverage of cytosines in CpG context with coverage in at least 3 replicates in both strains (CpGs on forward and reverse strand were counted separately). The dotted line marks the minimum coverage cut off for CpGs included in the differential methylation analysis. (PDF) [file pgen.1004813.s001.pdf]

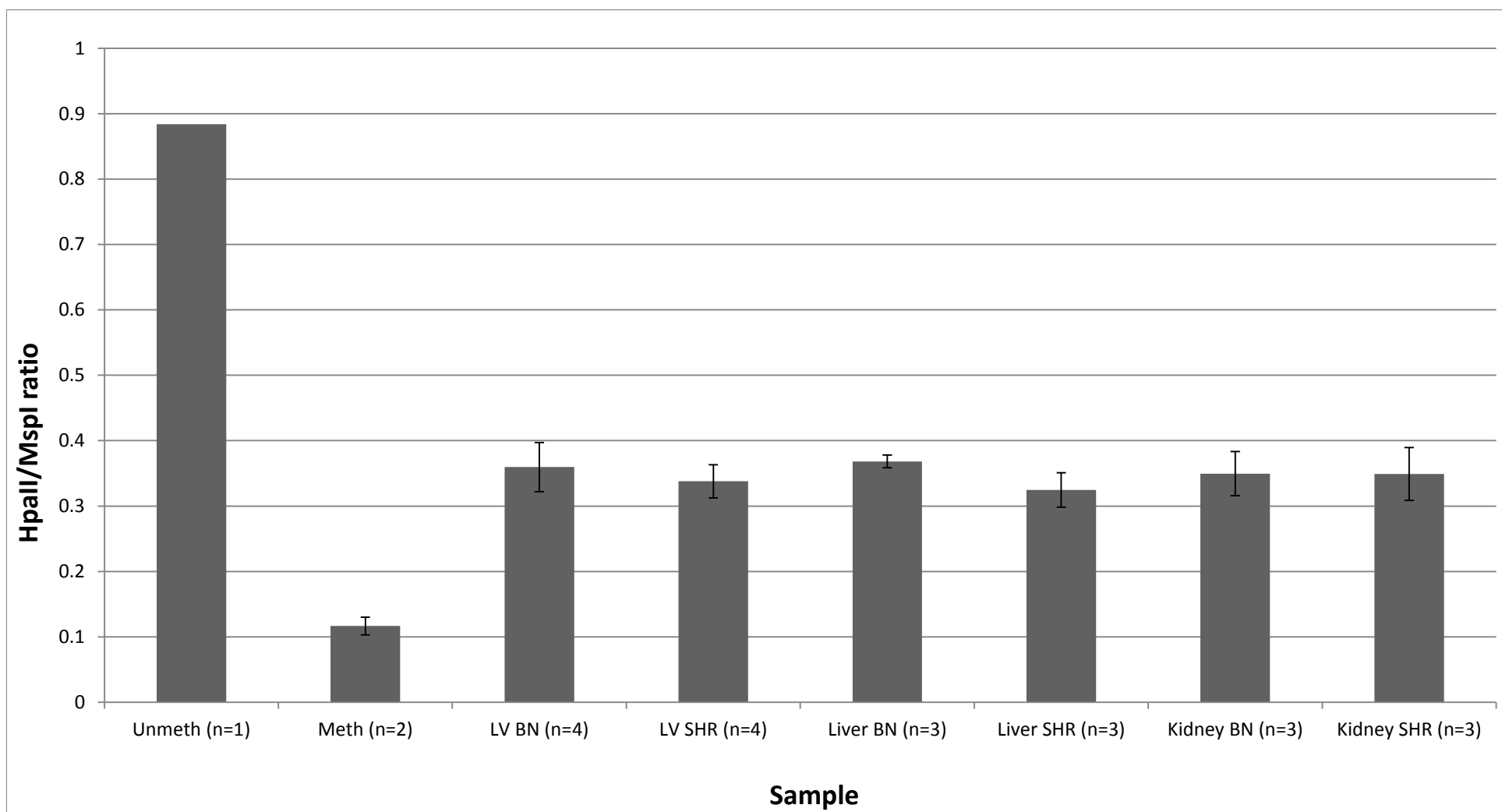

Figure S2

Supplement: Figure S2 — Analysis of global DNA methylation levels by Luminometric Methylation Assay (LUMA). Genomic DNA extracted from liver, kidney and left ventricle (LV) of BN and SHR animals was subjected to double-digestion with EcoRI and methylation-sensitive HpaII and methylation insensitive MspI restriction endonucleases and global methylation quantified by pyrosequencing. Unmeth = unmethylated control DNA, meth = fully methylated control DNA, n = replicates. The error bars indicate the standard deviation. (PDF) [file pgen.1004813.s002.pdf]

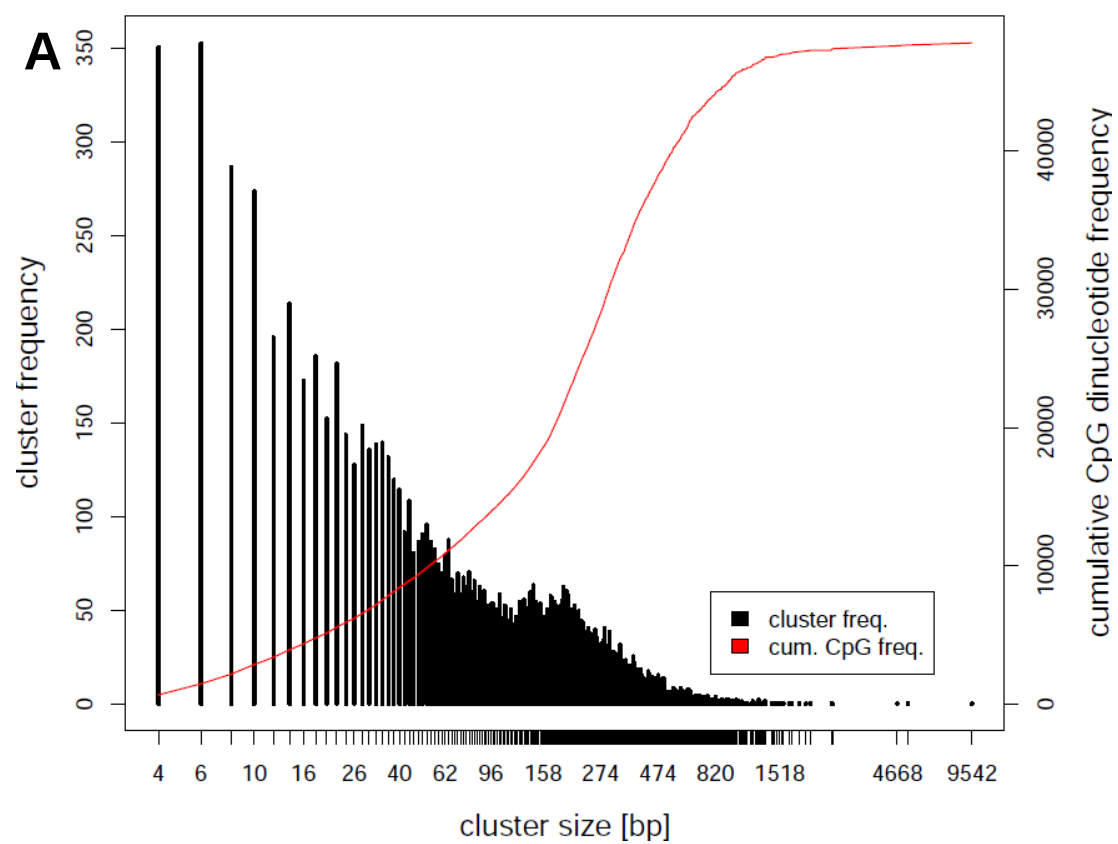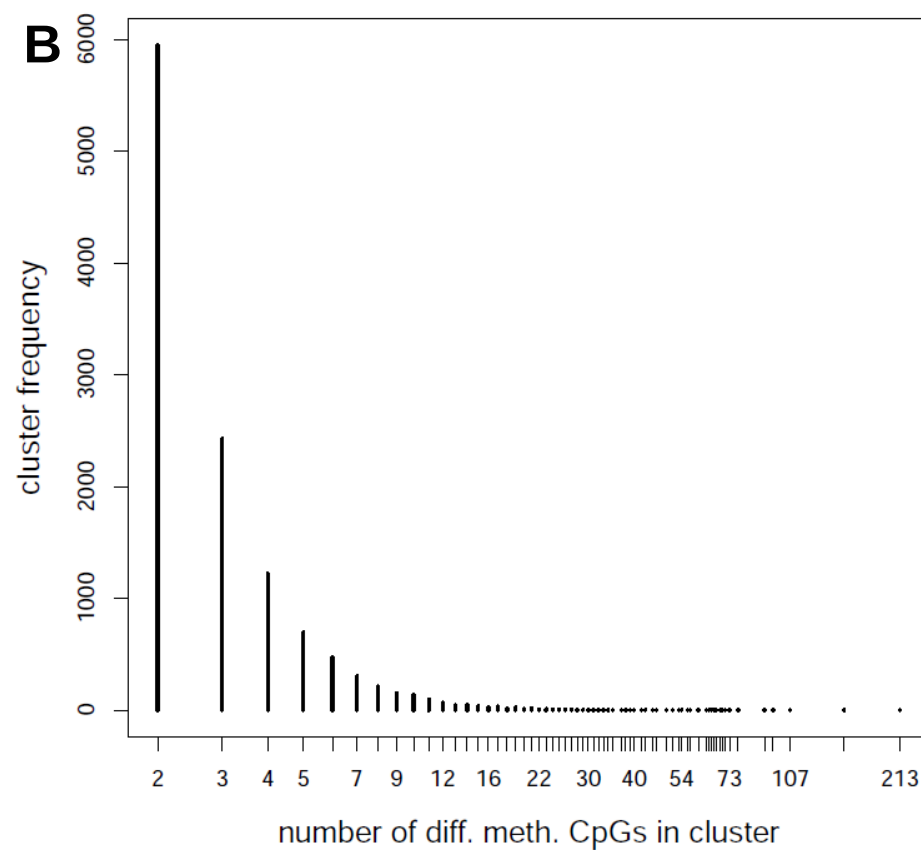

Figure S3

Supplement: Figure S3 — Clusters of differentially methylated CpG dinucleotides. (A) Frequency plot showing the size distribution of clusters of differentially methylated CpG dinucleotides (black bars, left-hand y-axis) obtained when grouping adjacent differentially methylated CpG dinucleotides not further than 500 bp away from each other. Singletons (29,313 differentially methylated CpG dinucleotides) are excluded from the plot. The red line shows the cumulative frequency of differentially methylated CpG dinucleotides contained within the clusters (right hand y-axis). (B) Frequency plot showing the number of differentially methylated CpG dinucleotides contained in the clusters of differentially methylated CpGs. (PDF) [file pgen.1004813.s003.pdf]

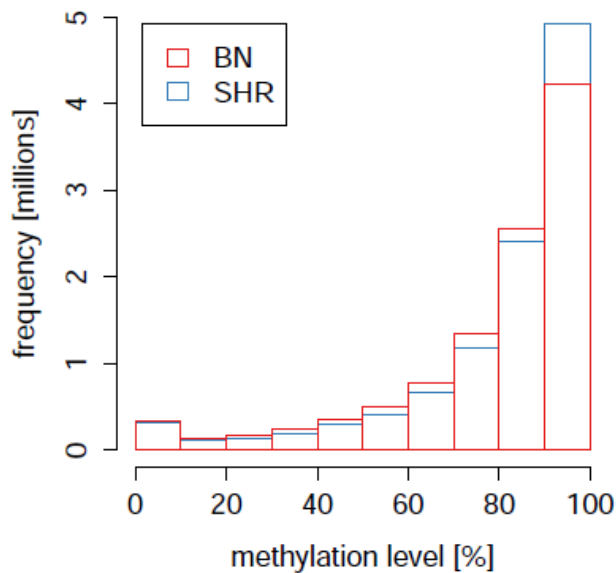

Figure S4

Supplement: Figure S4 — Distribution of methylation levels at CpG cytosines analysed for differential methylation between BN and SHR strains. The x-axis shows the average percent methylation across replicates and the y-axis the number of CpG di-nucleotides. (PDF) [file pgen.1004813.s004.pdf]

**A**

inside CGI

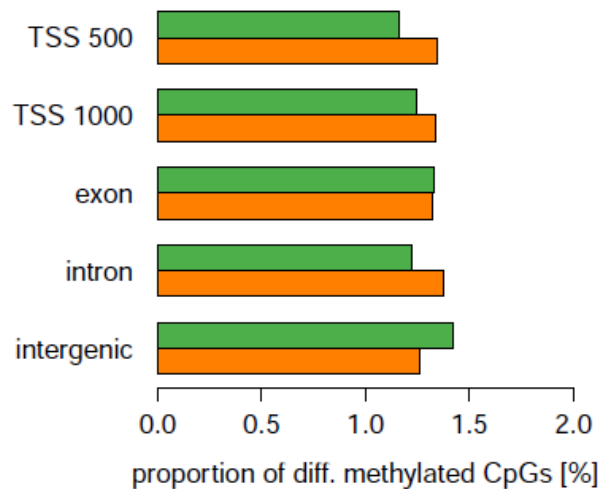**B**

outside CGI

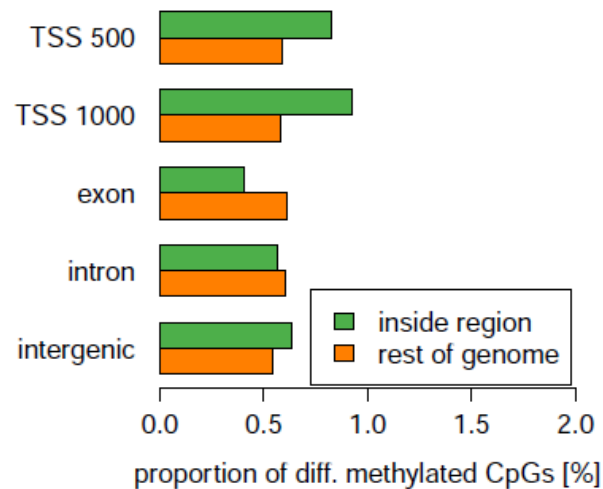

Figure S5

Supplement: Figure S5 — Distribution of methylation differences across genomic regions. (A) Proportion of differentially methylated CpGs within different genomic regions compared to the rest of the genome for CpGs inside CpG islands (TSS = transcription start site as annotated in Ensembl version 62; TSS 500 = region 500 bp upstream and downstream of TSS; TSS 1000 = region 1000 bp upstream and downstream of TSS). (B) Proportion of differentially methylated CpGs within different genomic regions compared to the rest of the genome for CpGs outside CpG islands. (PDF) [file pgen.1004813.s005.pdf]

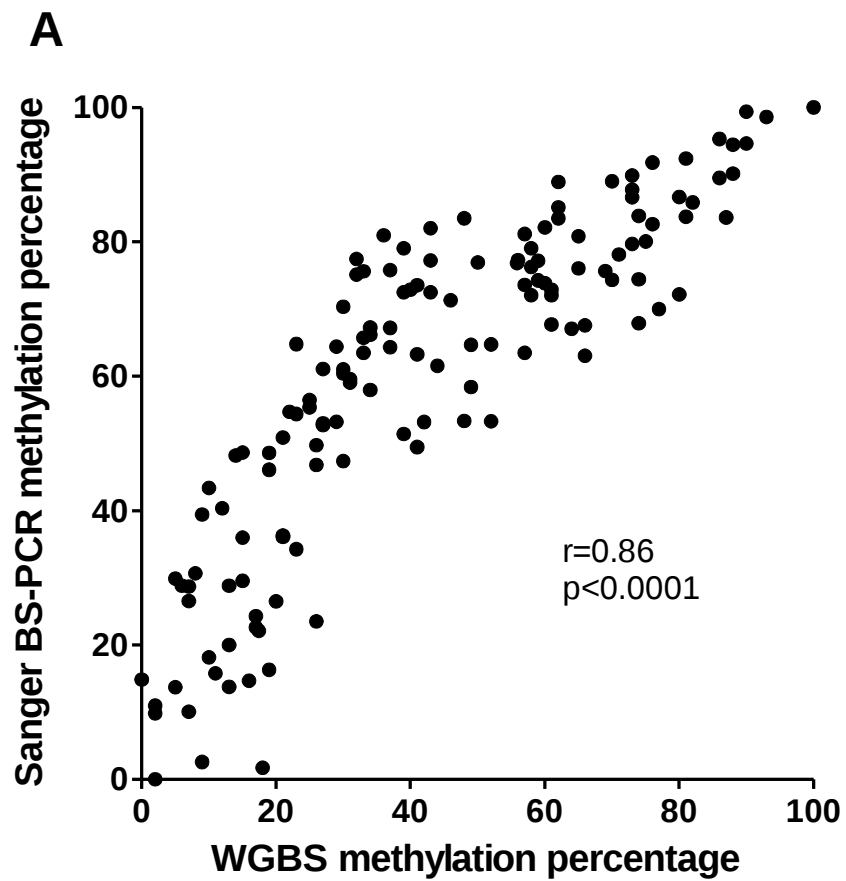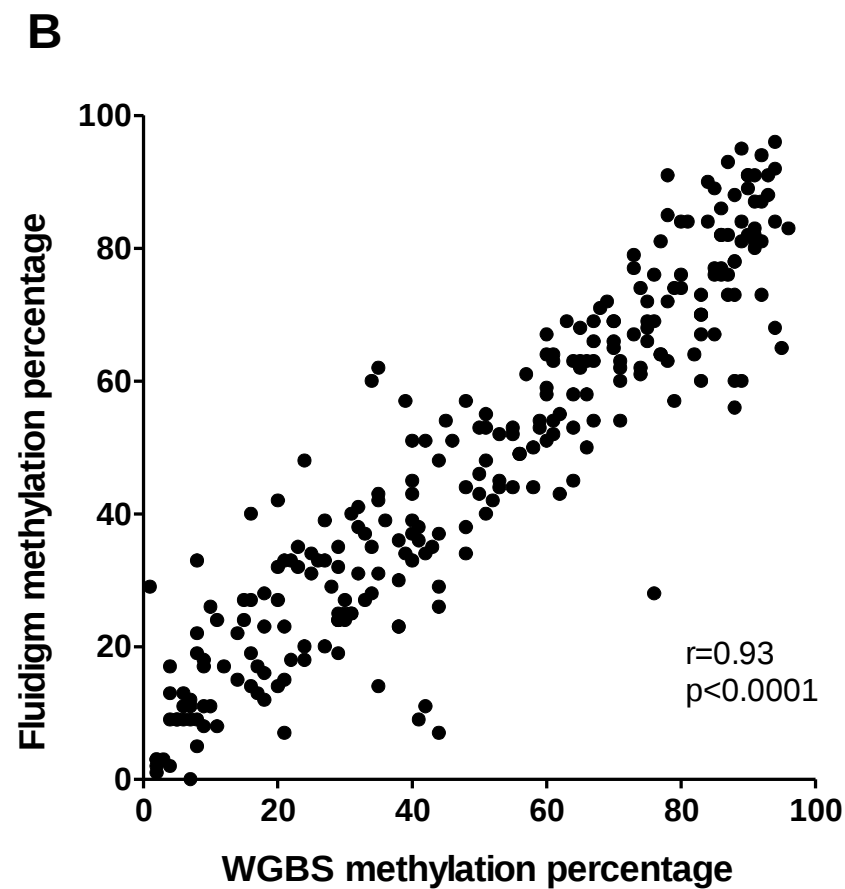

Figure S6

Supplement: Figure S6 — Scatter plots of methylation percentages obtained by Sanger sequencing or Fluidigm and whole genome bisulfite sequencing (WGBS) in the SHR and BN inbred strains. (A) Methylation data were obtained by Sanger sequencing (y-axis) and WGBS (x-axis) for 70 individual CpG dinucleotides in each of the Brown Norway (n = 4) and the spontaneously hypertensive rat (n = 4) strains. (B) Methylation data were obtained by Fluidigm (y-axis) and WGBS (x-axis) for 131 individual CpG dinucleotides in each of the Brown Norway (n = 4) and the spontaneously hypertensive rat (n = 4) strains. Pearson's correlation coefficent and associated p value are given. (PDF) [file pgen.1004813.s006.pdf]

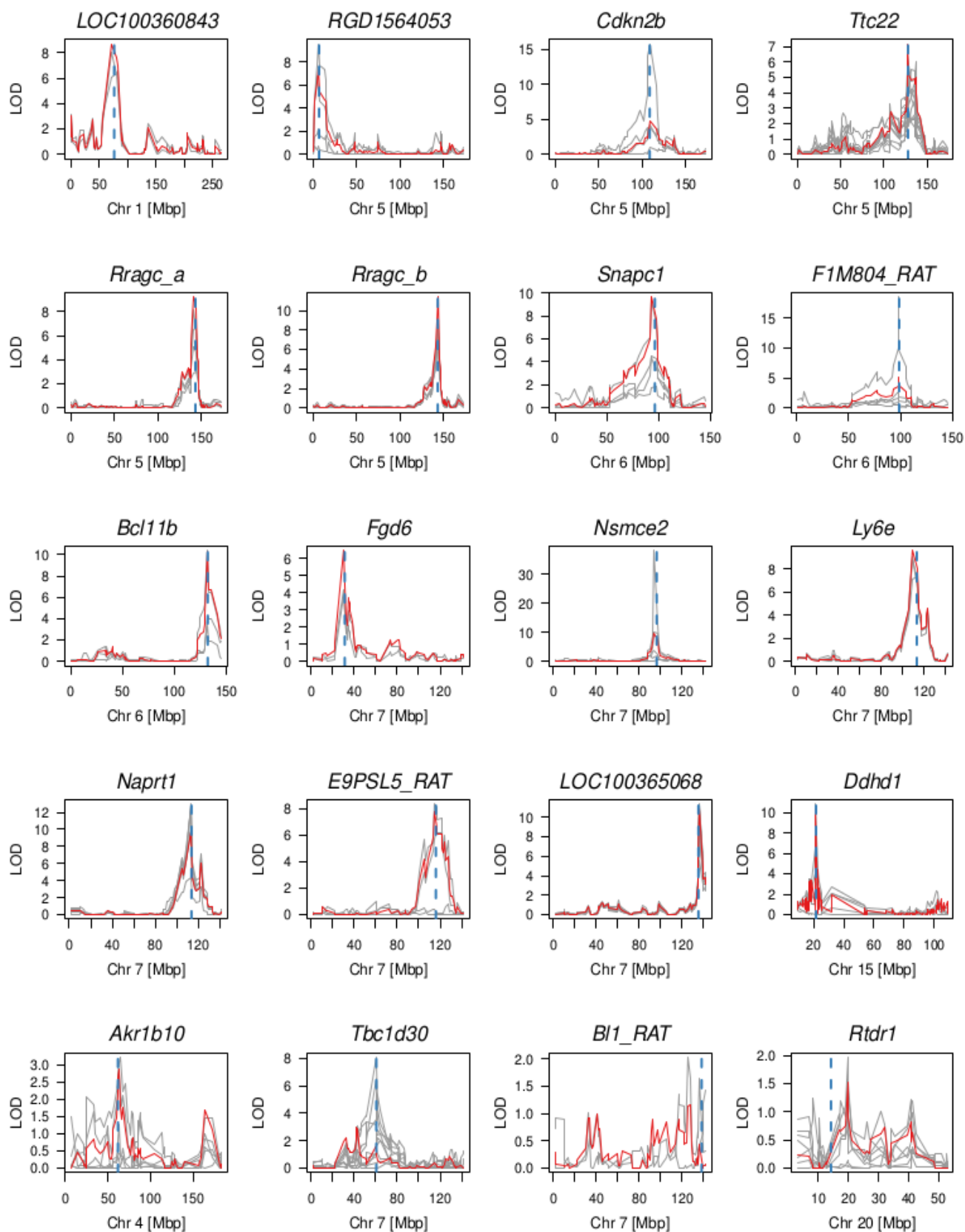

Figure S7

Supplement: Figure S7 — LOD plots of 20 meth-QTLs. The average methylation percentage across amplicons Akr1b10, Tbc1d30, Bl1_RAT and Rtdr1 did not show significant linkage. For Tbc1d30 the individual CpG dinucleotides mapped in cis. LOD scores for linkage of average amplicon methylation levels are shown in red, LOD scores for linkage of individual CpG dinucleotides are shown in grey. The peak of linkage is indicated by a dashed, blue vertical line. (PDF) [file pgen.1004813.s007.pdf]

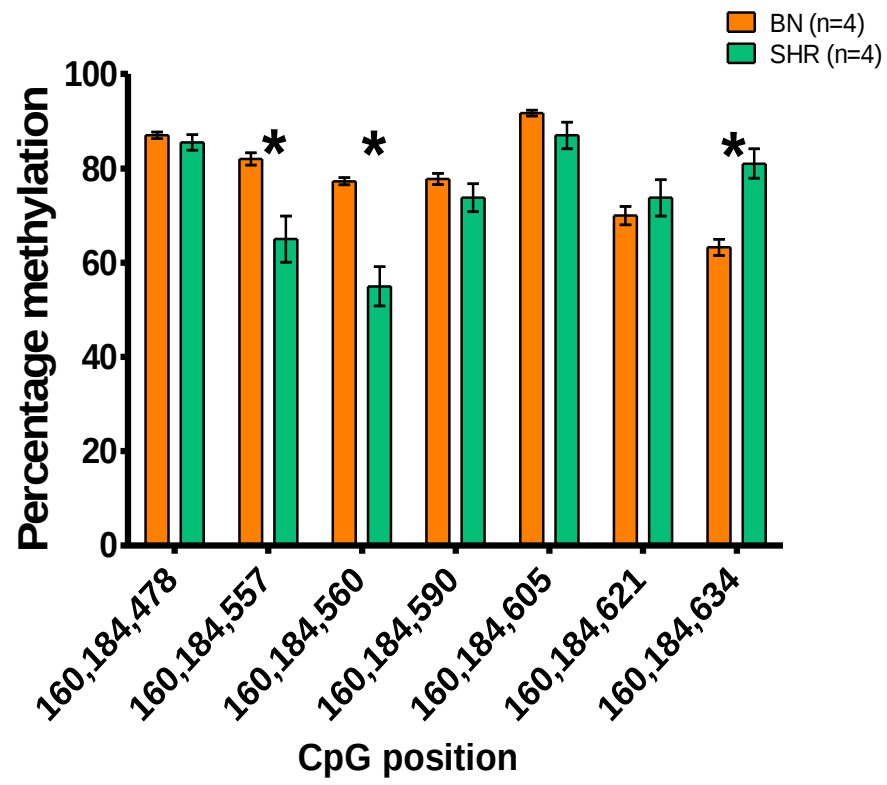

Figure S8

Supplement: Figure S8 — Methylation of individual CpG dinucleotides across the Epha2 amplicon measured by Sanger sequencing of PCR-amplified bisulfite-treated DNA. Seven CpG dinucleotides were assayed by Sanger sequencing with three (*) showing significant differential methylation (p<0.05) between the Brown Norway (BN) and the Spontaneously Hypertensive Rat (SHR). CpG dinucleotide 160,184,634 shows an opposing allelic effect as methylation is decreased in the BN strain whereas at CpG dinucleotides 160,184,557 and 160,184,560 methylation is increased in the BN strain compared to SHR. (PDF) [file pgen.1004813.s008.pdf]

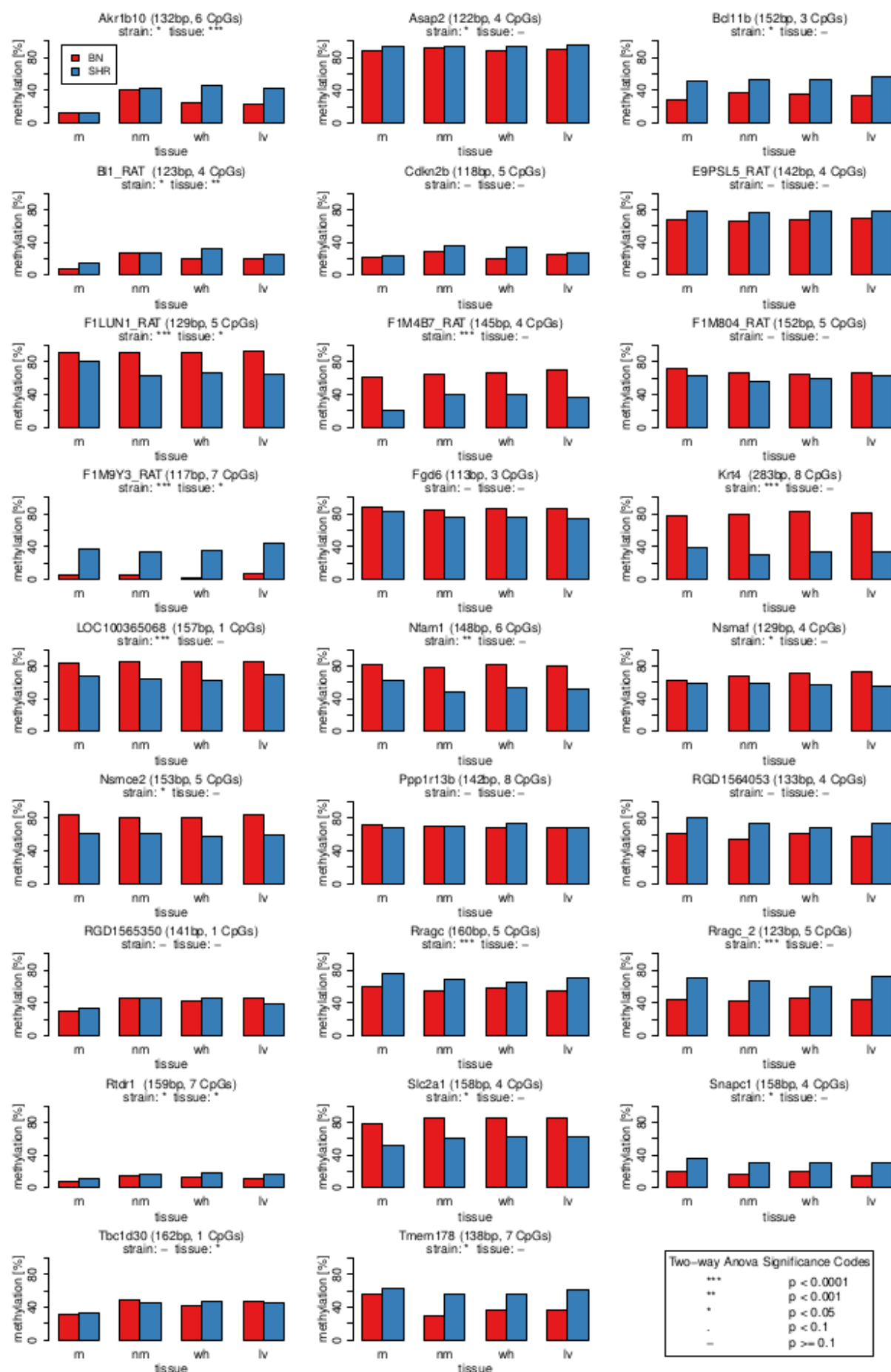

Figure S9

Supplement: Figure S9 — Methylation differences between BN and SHR across different cell- and tissue-samples. Shown are average methylation levels across each amplicon in cardiomyocyte preparations (m), non-cardiomyoctye preparations (nm), whole heart samples (wh) and left ventricle samples (lv). Amplicon length and number of measured CpGs are given in brackets behind the amplicon name. The significance of a two-way ANOVA testing for independence of CpG methylation levels and the two factors strain and tissue are shown below the amplicon name. (PDF) [file pgen.1004813.s009.pdf]

max. distance to  
nearest SNP  
[bp]

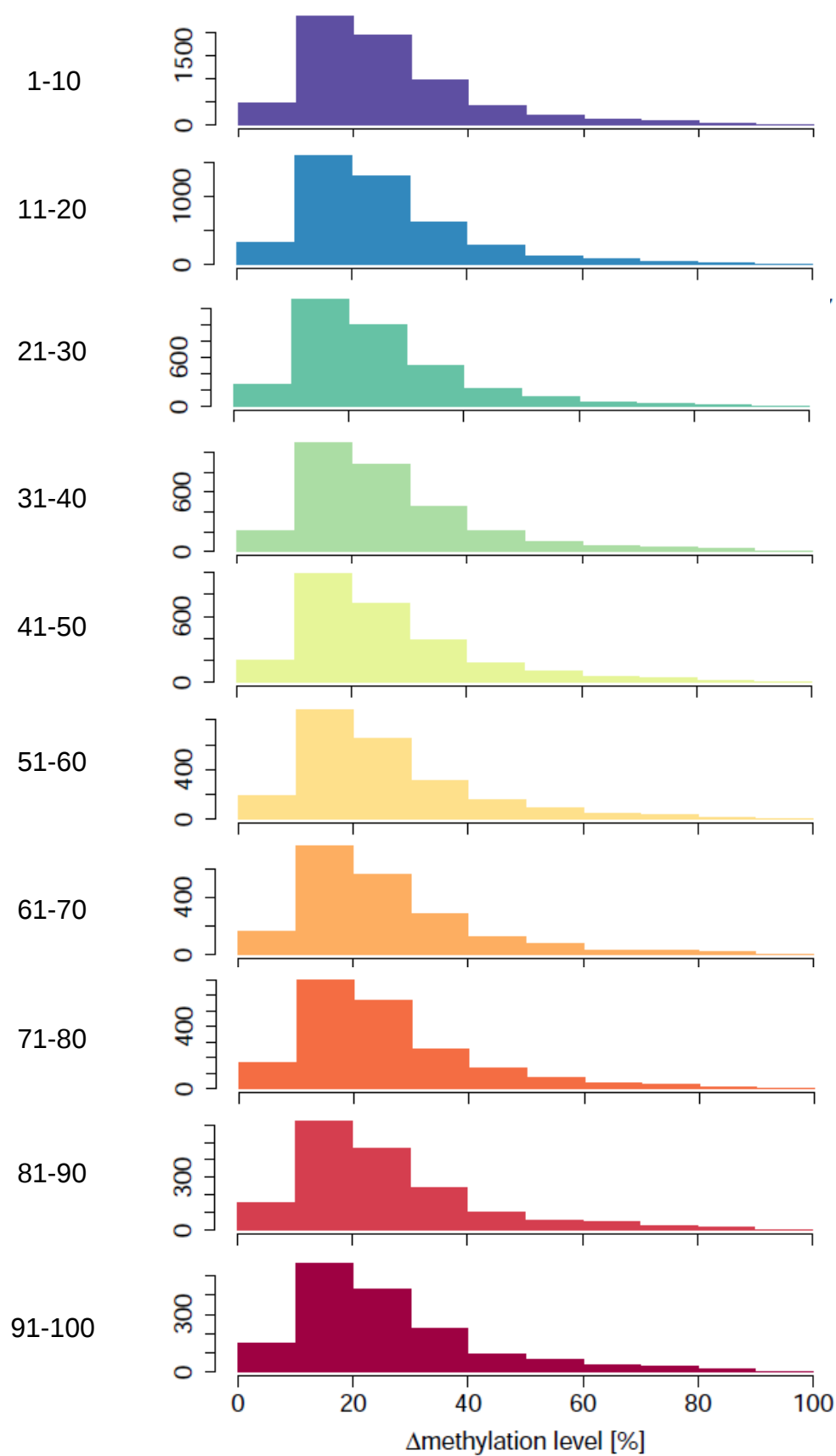

Figure S10

Supplement: Figure S10 — Distance to the nearest SNP and extent of differential methylation. Histograms showing the distribution of methylation differences between BN and SHR for CpGs at different distances from the nearest SNP. (PDF) [file pgen.1004813.s010.pdf]

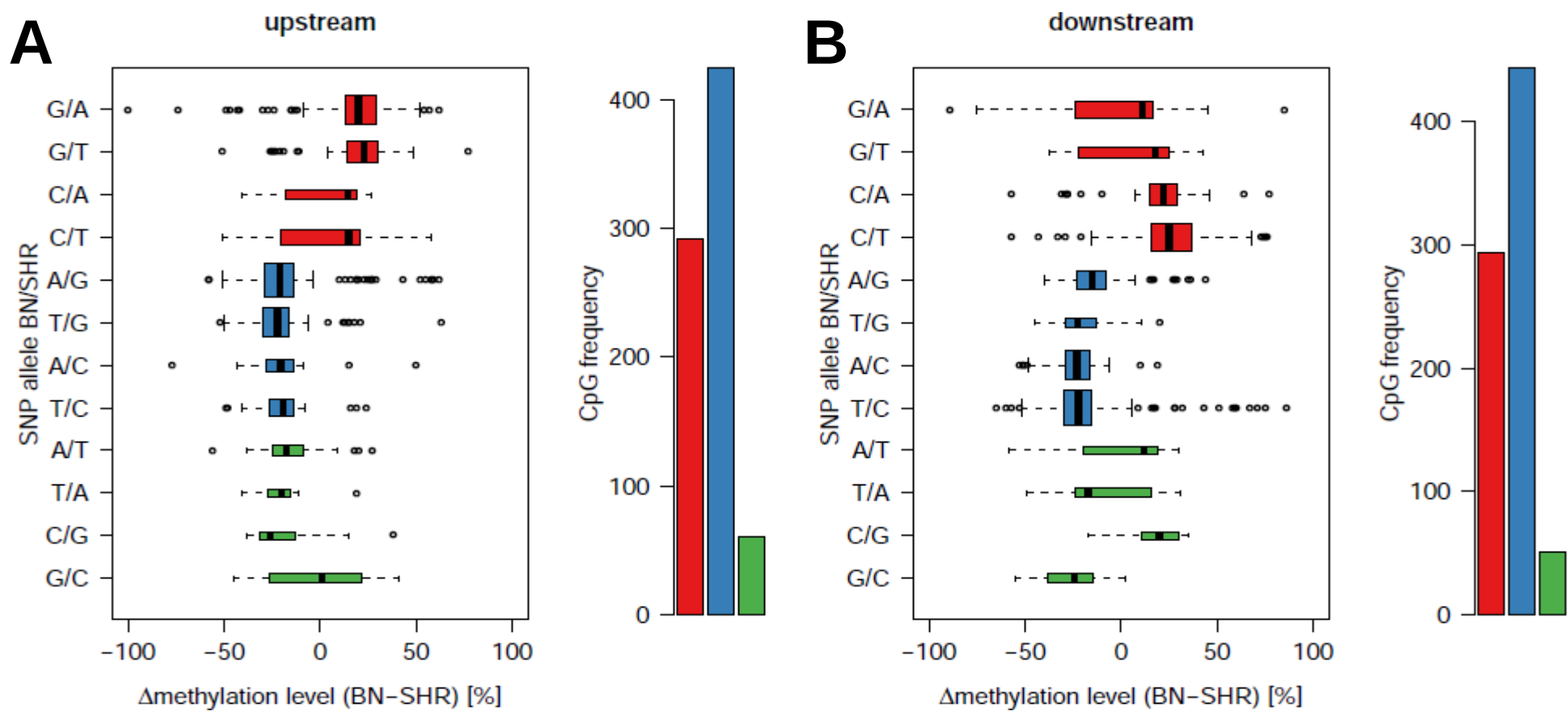

Figure S11

Supplement: Figure S11 — Distribution of methylation differences between the parental strains depending on the alleles of SNPs directly adjacent to the CpG dinucleotide. (A) Boxplot of the distribution of methylation differences for BN/SHR allele changes at the nucleotide directly upstream of the CpG dinucleotide that, based on the motif analysis, are predicted (i) to result in decreased methylation in SHR (red), (ii) to result in increased methylation in SHR (blue) or (iii) to have a neutral effect on methylation (green). The height of the box indicates the frequency of each allele change. The total observation frequency for each group of changes (increased, decreased, neutral) is shown by the bar plot on the right. (B) Boxplot of the distribution of methylation differences for BN/SHR allele changes at the nucleotide directly downstream of the CpG dinucleotide. (PDF) [file pgen.1004813.s011.pdf]

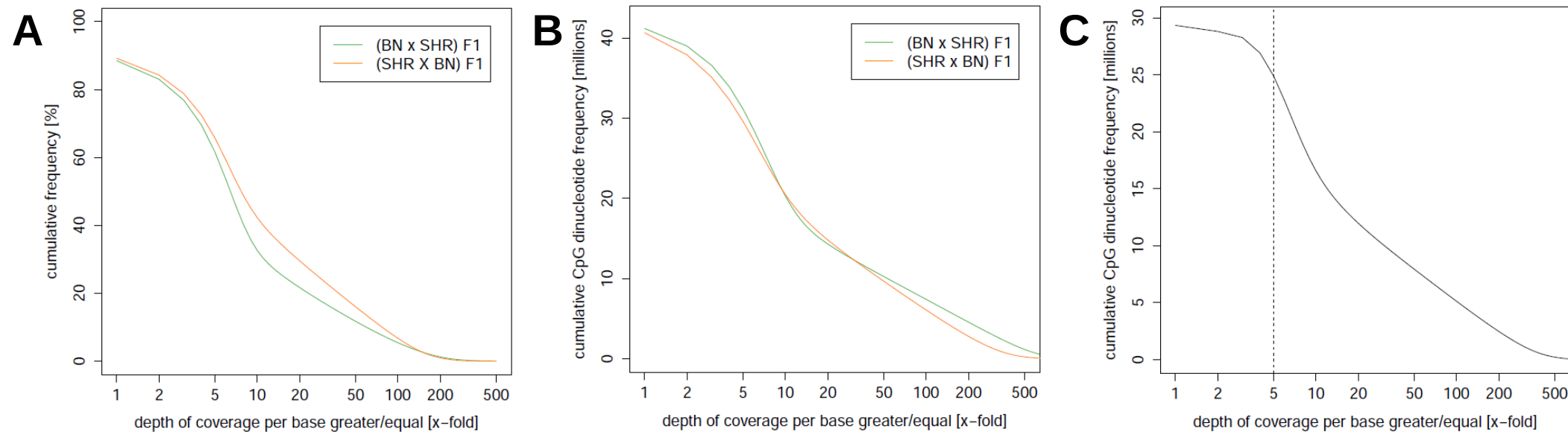

Figure S12

Supplement: Figure S12 — Depth of coverage of quality-filtered whole genome bisulfite sequencing data generated in the reciprocal F1 crosses. (A) Cumulative coverage across the genome. The plot shows the percentage of bases (ungapped sequence of the RGSC3.4 reference assembly) covered by at least x reads. (B) Cumulative coverage of cytosines in CpG context (CpGs on forward and reverse strand were counted separately) (C) Cumulative coverage of cytosines in CpG context with coverage in at least 3 replicates in both crosses (CpGs on forward and reverse strand were counted separately). The dotted line marks the minimum coverage cut-off for CpGs included in the differential methylation analysis. (PDF) [file pgen.1004813.s012.pdf]

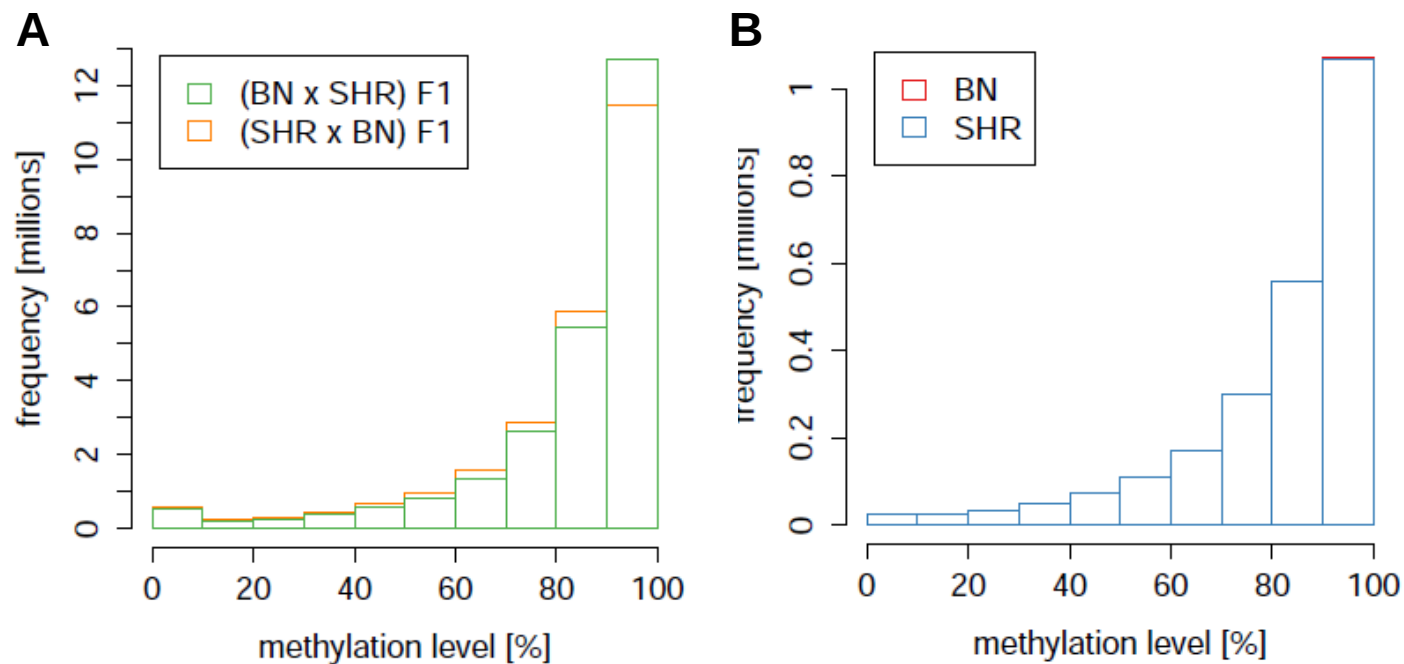

Figure S13

Supplement: Figure S13 — Differential CpG methylation between the reciprocal F1 crosses and allele-specific CpG methylation. (A) Distribution of relative methylation levels at cytosines analysed for differential methylation between the reciprocal F1 crosses. (B) Distribution of relative methylation levels at cytosines analysed for allele-specific methylation in the F1 crosses. The histograms show the distribution of methylation levels measured for the BN and the SHR allele. (PDF) [file pgen.1004813.s013.pdf]
