# Supplementary material for: Genetic Analysis of the Cardiac Methylome at Single Nucleotide Resolution in a Model of Human Cardiovascular Disease
Source: PLoS Genet. 2014 Dec 4;10(12):e1004813. doi: 10.1371/journal.pgen.1004813 (PMC4256262; doi:10.1371/journal.pgen.1004813)
Supplement: Table S1 — Mapping and filtering statistics for whole genome bisulfite sequencing reads generated in BN and SHR rats. (PDF) [file pgen.1004813.s014.pdf]

Table S1 Mapping and filtering statistics for whole genome bisulfite sequencing reads generated in BN and SHR rats

Sample	Total Reads	Mapped Reads		Mapped Bases	Depth of Coverage	Mapped Bases After Filtering	Depth of Coverage After Filtering
	[millions]	[millions]	[%]	[billions]	[x-fold]	[billions]	[x-fold]
BN1	258	230	89	17.1	6.7	11.3	4.4
BN2	413	361	87	27.4	12.6	18.5	7.2
BN3	454	404	89	31.9	10.7	21.6	8.4
BN4	370	340	92	32.3	12.4	22.7	8.8
**BN total**	**1,495**	**1,336**	**89**	**108.7**	**42.3**	**74.1**	**28.8**
SHR1	497	448	91	38.4	14.9	26.3	10.2
SHR2	452	398	89	30.2	11.7	20.8	8.1
SHR3	421	369	90	28.3	11.0	19.4	7.5
SHR4	391	356	91	30.7	11.9	21.2	8.2
**SHR total**	**1,762**	**1,571**	**89**	**127.6**	**49.6**	**87.7**	**34.1**
