# Supplementary material for: Genetic Analysis of the Cardiac Methylome at Single Nucleotide Resolution in a Model of Human Cardiovascular Disease
Source: PLoS Genet. 2014 Dec 4;10(12):e1004813. doi: 10.1371/journal.pgen.1004813 (PMC4256262; doi:10.1371/journal.pgen.1004813)
Supplement: Table S2 — Gene Ontology analysis of genes associated with differentially methylated regions. (PDF) [file pgen.1004813.s015.pdf]

Table S2 Gene Ontology analysis of genes associated with differentially methylated regions

Ontology	Term ID	Term	Genes with annotation	Genes annotated with term	Fold enrichment	P value	P value FDR adjusted
**cellular component**	**GO:0005886**	**plasma membrane**	**780**	**259**	**1.6**	**3.83E-17**	**1.21E-14**
cellular component	*GO:0044459*	*plasma membrane part*	**780**	**155**	**1.7**	**2.34E-11**	**3.70E-09**
**cellular component**	**GO:0005856**	**cytoskeleton**	**780**	**95**	**1.5**	**2.30E-05**	**1.81E-03**
**cellular component**	**GO:0005737**	**cytoplasm**	**780**	**459**	**1.1**	**2.74E-05**	**1.73E-03**
**cellular component**	**GO:0043005**	**neuron projection**	**780**	**55**	**1.8**	**1.62E-05**	**1.71E-03**
**biological process**	**GO:0030182**	**neuron differentiation**	**745**	**60**	**2.0**	**7.37E-07**	**2.92E-04**
biological process	GO:0048666	*neuron development*	**745**	**48**	**2.1**	**2.56E-06**	**5.79E-04**
biological process	*GO:0048513*	*organ development*	**745**	**168**	**1.5**	**4.19E-09**	**6.65E-06**
biological process	*GO:0000904*	*cell morphogenesis involved in differentiation*	**745**	**38**	**2.4**	**1.45E-06**	**4.60E-04**
biological process	*GO:0007399*	*nervous system development*	**745**	**100**	**1.5**	**1.70E-05**	**2.44E-03**
biological process	GO:0048812	*neuron projection morphogenesis*	**745**	**33**	**2.3**	**1.19E-05**	**1.89E-03**
biological process	GO:0022008	*neurogenesis*	**745**	**73**	**1.7**	**9.74E-06**	**1.71E-03**
biological process	*GO:0032989*	*cellular component morphogenesis*	**745**	**48**	**1.9**	**2.57E-05**	**3.39E-03**
biological process	*GO:0048468*	*cell development*	**745**	**76**	**1.7**	**4.38E-06**	**8.67E-04**
**biological process**	**GO:0010646**	**regulation of cell communication**	**745**	**103**	**1.6**	**5.42E-07**	**2.86E-04**
**biological process**	**GO:0009966**	**regulation of signal transduction**	**745**	**84**	**1.7**	**2.35E-06**	**6.20E-04**
**biological process**	**GO:0009888**	**tissue development**	**745**	**80**	**1.9**	**1.56E-08**	**1.24E-05**

Shown are terms statistically significantly enriched ($p < 0.05$) after fals discovery rate (FDR) adjustment. Terms were clustered with REVIGO (Supek et al, 2011). Cluster representatives are shown in bold, more specific cluster members are shown below in italics.
