# Supplementary material for: Genetic Analysis of the Cardiac Methylome at Single Nucleotide Resolution in a Model of Human Cardiovascular Disease
Source: PLoS Genet. 2014 Dec 4;10(12):e1004813. doi: 10.1371/journal.pgen.1004813 (PMC4256262; doi:10.1371/journal.pgen.1004813)
Supplement: Table S3 — CpG dinucleotides tested for linkage in recombinant inbred strains. (PDF) [file pgen.1004813.s016.pdf]

Table S3 CpG dinucleotides tested for linkage in recombinant inbred strains

Gene ID[a]	CpG chromosome, coordinate and strand	WGBS BN[b]	WGBS SHR[b]	WGBS difference[b]	BN (n=4)[c]	SHR (n=4)[c]	Difference[c]	Peak of linkage coordinate[d]	LOD score[e]	2-LOD interval	Linkage type	Sanger/ Illumina[f]
LOC100360843	chr1:76260384:+	26	69	-43	50	76	-26	chr1:80926785	6.34	chr1:55981210-89159471	*cis*	S
LOC100360843	chr1:76260390:+	21	65	-44	36	76	-40	chr1:71780124	8.06	chr1:55981210-89159471	*cis*	S
***LOC100360843* average**	**chr1:76260305-76260666:+**				**43**	**76**	**-33**	**chr1:71780124**	**8.70**	**chr1:55981210-89159471**	***cis***	S
Cast	chr2:1577794:-	nd[g]	nd	nd	100	1	99	chr2:8864577	9.62	chr2:0-13411541	*cis*	S
Cast	chr2:1577799:-	nd	nd	nd	93	1	93	chr2:8864577	10.30	chr2:0-13411541	*cis*	S
Cast	chr2:1577803:-	nd	nd	nd	95	1	94	chr2:8864577	11.05	chr2:0-13411541	*cis*	S
Cast	chr2:1577819:-	nd	nd	nd	88	1	87	chr2:8864577	12.91	chr2:0-13411541	*cis*	S
Cast	chr2:1577857:-	nd	nd	nd	97	2	95	chr2:8864577	12.29	chr2:0-13411541	*cis*	S
Cast	chr2:1577875:-	nd	nd	nd	96	7	89	chr2:8864577	12.18	chr2:0-13411541	*cis*	S
Cast	chr2:1577880:-	nd	nd	nd	99	4	95	chr2:8864577	12.41	chr2:0-13411541	*cis*	S
Cast	chr2:1577899:-	nd	nd	nd	100	2	98	chr2:8864577	12.51	chr2:0-13411541	*cis*	S
Cast	chr2:1577909:-	90	18	72	99	2	98	chr2:8864577	12.50	chr2:0-13411541	*cis*	S
Cast	chr2:1577916:-	nd	nd	nd	88	2	86	chr2:8864577	12.78	chr2:0-13411541	*cis*	S
Cast	chr2:1577947:-	100	16	84	100	15	85	chr2:8864577	12.20	chr2:0-13411541	*cis*	S
***Cast* average**	**chr2:1577677-1577984:-**				**96**	**3**	**93**	**chr2:8864577**	**12.57**	**chr2:0-13411541**	***cis***	S
Fam198b	chr2:171370578:+	61	49	12	72	58	14	chr2:166553647	8.81	chr2:164055359-172685103	*cis*	S
Fam198b	chr2:171370584:+	75	52	23	80	65	15	chr2:166553647	11.26	chr2:164055359-172685103	*cis*	S
Fam198b	chr2:171370615:+	76	57	19	92	74	18	chr2:166553647	12.22	chr2:164055359-172685103	*cis*	S
Fam198b	chr2:171370624:+	86	58	28	90	72	17	chr2:166553647	11.34	chr2:164055359-172685103	*cis*	S
***Fam198b* average**	**chr2:171370462-171370757:+**				**83**	**67**	**16**	**chr2:166553647**	**13.87**	**chr2:164055359-172685103**	***cis***	S
Odpf2	chr3:8876367:+	40	60	-20	73	82	-9	chr2:82938235	7.57	chr2:82818916-83039313	*trans*	S
Odpf2	chr3:8876373:+	29	50	-21	64	77	-13	chr2:82938235	5.79	chr2:82282002-83709691	*trans*	S
Odpf2	chr3:8876382:+	27	46	-19	61	71	-10	chr2:82938235	4.47	chr2:79300696-83709691	*trans*	S
Odpf2	chr3:8876397:+	37	62	-25	76	85	-9	chr2:82938235	5.05	chr2:79300696-83709691	*trans*	S
Odpf2	chr3:8876402:+	48	73	-25	84	90	-6	chr2:82938235	5.82	chr2:79300696-83709691	*trans*	S
Odpf2	chr3:8876433:+	41	62	-21	74	84	-10	chr2:82938235	3.59	chr2:75338743-83709691	*trans*	S
Odpf2	chr3:8876480:+	9	14	-5	39	48	-9	chr2:82282002	2.51	n/a[h]	no signif linkage[i]	S
Odpf2	chr3:8876484:+	10	25	-15	43	55	-12	chr2:82282002	2.27	n/a	no signif linkage	S
***Odfp2* average**	**chr3:8876123-8876785:+**				**72**	**81**	**-10**	**chr2:82938235**	**6.10**	**chr2:79300696-83039313**	***trans***	S
Akr1b10	chr4:61757643:+	8	8	8	22	33	-12	chr17:39969064	1.95	n/a	no signif linkage	I
Akr1b10	chr4:61757653:+	18	18	18	16	28	-12	chr17:28025480	2.23	n/a	no signif linkage	I
Akr1b10	chr4:61757680:+	6	29	-23	9	19	-10	chr15:54570481	2.23	n/a	no signif linkage	I
Akr1b10	chr4:61757702:+	4	4	4	9	17	-8	chr17:24218005	3.09	n/a	no signif linkage	I
Akr1b10	chr4:61757723:+	20	20	20	32	42	-11	chr4:64105027	3.23	n/a	no signif linkage	I
Akr1b10	chr4:61757729:+	16	16	16	27	40	-13	chr1:237021546	3.94	chr1:236899082-240673620	*trans*	I
***Akr1b10* average**	**chr4:61757621-61757753:+**				**19**	**30**	**-11**	**chr4:63305380**	**2.91**	**n/a**	***no signif linkage***	I
RGD1564053	chr5:6849865:+	21	39	-18	15	34	-19	chr5:4525855	6.83	chr5:1894554-7625963	*cis*	I
RGD1564053	chr5:6849899:+	66	89	-23	50	81	-31	chr5:6419261	9.57	chr5:1894554-13327089	*cis*	I
RGD1564053	chr5:6849920:+	73	83	-10	67	73	-6	chr5:6419261	2.16	n/a	no signif linkage	I
RGD1564053	chr5:6849925:+	96	92	4	83	81	2	chr14:36607884	2.06	n/a	no signif linkage	I
***RGD1564053* average**	**chr5:6849814-6849947:+**				**54**	**67**	**-13**	**chr5:6419261**	**7.35**	**chr5:1894554-13327089**	***cis***	I
Nsmaf	chr5:19887393:+	73	70	3	77	65	12	chr5:17516274	4.87	chr5:14415906-24215534	*cis*	I
Nsmaf	chr5:19887422:+	64	51	13	58	53	6	chr5:17516274	4.20	chr5:4525855-24215534	*cis*	I
Nsmaf	chr5:19887432:+	74	61	13	74	54	20	chr5:17516274	8.05	chr5:16184249-24215534	*cis*	I
Nsmaf	chr5:19887480:+	60	40	20	67	51	16	chr5:17516274	11.22	chr5:16184249-20153421	*cis*	I
***Nsmaf* average**	**chr5:19887357-19887486:+**				**69**	**56**	**13**	**chr5:17516274**	**11.67**	**chr5:16184249-20153421**	***cis***	I

RGD1565350	chr5:62118122:+	29	14	14	24	15	9	chr5:56293047	3.93	chr5:40389315-58060089	*cis*	I
RGD1565350	chr5:62118163:+	61	40	21	63	39	25	chr5:62039612	10.42	chr5:61230757-64094065	*cis*	I
RGD1565350	chr5:62118183:+	48	34	14	44	28	16	chr5:62039612	12.43	chr5:61230757-64094065	*cis*	I
RGD1565350	chr5:62118194:+	50	38	12	43	30	13	chr5:62039612	7.93	chr5:61230757-64094065	*cis*	I
***RGD1565350* average**	**chr5:62118077-62118218:+**				**44**	**28**	**16**	**chr5:62039612**	**12.22**	**chr5:61230757-64094065**	***cis***	I
Galnt12	chr5:63712047:+	42	71	-29	34	63	-30	chr5:62039612	7.53	chr5:58060089-64694890	*cis*	I
Galnt12	chr5:63712080:+	51	67	-16	40	66	-25	chr5:64094065	4.77	chr5:58060089-74190073	cis	I
Galnt12	chr5:63712088:+	58	68	-10	44	71	-27	chr5:62039612	7.62	chr5:58501829-64094065	*cis*	I
Galnt12	chr5:63712117:+	16	29	-13	14	24	-10	chr5:62039612	3.04	n/a	no signif linkage	I
Galnt12	chr5:63712134:+	34	67	-33	35	63	-28	chr5:62039612	9.54	chr5:58060089-64094065	*cis*	I
***Galnt12* average**	**chr5:63712033-63712186:+**				**34**	**57**	**-24**	**chr5:62039612**	**12.09**	**chr5:61230757-64094065**	***cis***	I
F1M4B7_RAT	chr5:87563006:+	59	33	26	54	27	27	chr5:83313363	13.28	chr5:83167847-88056807	*cis*	I
F1M4B7_RAT	chr5:87563013:+	53	30	23	45	24	21	chr5:83313363	12.80	chr5:83167847-88056807	*cis*	I
F1M4B7_RAT	chr5:87563081:+	86	53	33	82	44	38	chr5:83313363	18.66	chr5:83167847-88056807	*cis*	I
F1M4B7_RAT	chr5:87563083:+	76	43	33	69	35	34	chr5:83313363	14.20	chr5:83167847-88056807	*cis*	I
***F1M4B7_RAT* average**	**chr5:87563005-87563150:+**				**62**	**32**	**30**	**chr5:83313363**	**16.98**	**chr5:83167847-88056807**	***cis***	I
Cdkn2b	chr5:108937537:+	9	18	-9	17	23	-6	chr5:108704737	4.24	chr5:97254326-120148225	*cis*	I
Cdkn2b	chr5:108937541:+	7	16	-9	12	19	-6	chr5:108704737	3.89	chr5:103285990-120148225	*cis*	I
Cdkn2b	chr5:108937609:+	35	42	-7	42	51	-9	chr1:234601040	2.10	n/a	no signif linkage	I
Cdkn2b	chr5:108937640:+	15	41	-26	24	36	-12	chr5:108704737	5.89	chr5:106314352-119017376	*cis*	I
***Cdkn2b* average**	**chr5:108937522-108937640:+**				**24**	**32**	**-8**	**chr5:108704737**	**4.79**	**chr5:106314352-119017376**	***cis***	I
Ttc22	chr5:127700274:+	23	41	-18	34	49	-15	chr5:127023735	6.54	chr5:124763813-137473830	*cis*	S
Ttc22	chr5:127700277:+	15	30	-15	30	47	-18	chr5:127023735	6.24	chr5:126790264-130615503	*cis*	S
Ttc22	chr5:127700328:+	21	39	-18	36	51	-15	chr5:127023735	5.75	chr5:124763813-135115333	*cis*	S
Ttc22	chr5:127700333:+	19	49	-30	46	65	-19	chr5:127023735	4.05	124763813-141408867	*cis*	S
Ttc22	chr5:127700335:+	31	56	-25	59	77	-18	chr5:127023735	4.77	chr5:124763813-137473830	*cis*	S
Ttc22	chr5:127700343:+	30	70	-40	61	74	-13	chr5:130615503	5.51	chr5:124763813-136970505	*cis*	S
Ttc22	chr5:127700353:+	33	58	-25	63	76	-13	chr5:135115333	3.59	n/a	no signif linkage	S
Ttc22	chr5:127700355:+	31	60	-29	60	74	-14	chr5:127023735	5.81	chr5:124763813-136072542	*cis*	S
***Ttc22* average**	**chr5:127700207-127700483:+**				**47**	**63**	**-16**	**chr5:127023735**	**7.19**	**chr5:126790264-130615503**	***cis***	S
Slc2a1	chr5:139703426:+	88	91	-3	88	81	7	chr4:51563781	1.73	n/a	no signif linkage	I
Slc2a1	chr5:139703437:+	94	93	1	96	91	5	chr5:147970080	2.29	n/a	no signif linkage	I
Slc2a1	chr5:139703480:+	59	62	-3	53	43	10	chr4:51563781	1.88	n/a	no signif linkage	I
Slc2a1	chr5:139703549:+	87	7	80	76	0	76	chr5:137825190	31.98	chr5:137473830-141408867	*cis*	I
***Slc2a1* average**	**chr5:139703426-139703584:+**				**78**	**54**	**24**	**chr5:137825190**	**18.10**	**chr5:137473830-141408867**	***cis***	I
Rragc	chr5:143257694:+	48	55	-7	34	52	-18	chr5:143426944	4.30	chr5:137473830-145136680	*cis*	I
Rragc	chr5:143257762:+	48	67	-19	38	54	-16	chr5:143426944	6.60	chr5:137825190-145136680	*cis*	I
Rragc	chr5:143257789:+	56	83	-28	49	67	-18	chr5:141408867	8.18	chr5:137825190-145136680	*cis*	I
Rragc	chr5:143257837:+	51	69	-18	48	72	-24	chr5:141408867	5.11	chr5:137473830-145136680	*cis*	I
***Rragc* average**	**chr5:143257694-143257854:+**				**42**	**61**	**-19**	**chr5:141408867**	**9.20**	**chr5:137825190-145136680**	***cis***	I
Rragc	chr5:143573598:+	31	64	-33	25	63	-37	chr5:143426944	8.31	chr5:141408867-145136680	*cis*	I
Rragc	chr5:143573621:+	30	71	-41	27	60	-33	chr5:143426944	8.86	chr5:141408867-145136680	*cis*	I
Rragc	chr5:143573627:+	60	94	-33	51	84	-34	chr5:143426944	6.70	chr5:141408867-145136680	*cis*	I
Rragc	chr5:143573649:+	27	64	-37	20	53	-33	chr5:143426944	7.32	chr5:141408867-145136680	*cis*	I
Rragc	chr5:143573687:+	41	75	-33	38	72	-34	chr5:143426944	8.46	chr5:141408867-145136680	*cis*	I
***Rragc* average**	**chr5:143573578-143573701:+**				**32**	**66**	**-34**	**chr5:143426944**	**11.40**	**chr5:141408867-145136680**	***cis***	I
Epha2	chr5:160184557:+	43	23	20	82	65	17	chr5:159699655	5.96	chr5:158655817-163883415	*cis*	S
Epha2	chr5:160184560:+	43	22	21	77	55	23	chr5:159699655	9.85	chr5:159338084-163883415	*cis*	S
Epha2	chr5:160184634:+	41	57	-16	63	81	-18	chr5:159699655	9.39	chr5:159338084-163883415	*cis*	S
***Epha2* average**	**chr5:160184417-160184704:+**				**74**	**67**	**7**	**chr20:2931904**	**2.38**	**n/a**	***no signif linkage***	S

Tmem178	chr6:3892309:+	32	85	-53	31	67	-37	chr6:3837300	17.72	chr6:270828-5001565	*cis*	I
Tmem178	chr6:3892344:+	30	88	-59	25	60	-35	chr6:3837300	15.16	chr6:270828-5001565	*cis*	I
Tmem178	chr6:3892374:+	29	71	-42	24	54	-30	chr6:3837300	11.70	chr6:270828-5001565	*cis*	I
Tmem178	chr6:3892397:+	38	88	-49	23	56	-33	chr6:3837300	15.19	chr6:270828-5001565	*cis*	I
Tmem178	chr6:3892406:+	44	79	-36	26	57	-31	chr6:3837300	17.81	chr6:270828-5001565	*cis*	I
Tmem178	chr6:3892412:+	18	44	-26	12	29	-16	chr6:3837300	8.44	chr6:270828-5001565	*cis*	I
***Tmem178* average**	**chr6:3892290-3892428:+**				**24**	**54**	**-30**	**chr6:3837300**	**17.98**	**chr6:270828-5001565**	***cis***	I
Asap2	chr6:41745653:+	88	91	-3	78	83	-4	chr11:82046320	3.11	n/a	no signif linkage	I
Asap2	chr6:41745660:+	94	95	-1	68	65	3	chr6:115004085	3.10	n/a	no signif linkage	I
Asap2	chr6:41745686:+	67	90	-22	69	91	-22	chr6:40832900	6.32	chr6:38837057-44677919	*cis*	I
Asap2	chr6:41745719:+	85	90	-5	89	82	7	chr8:43888617	2.58	n/a	no signif linkage	I
***Asap2* average**	**chr6:41745647-41745769:+**				**76**	**80**	**-4**	**chr1:249360014**	**4.88**	**chr1:240992368-252871262**	***trans***	I
F1LUN1_RAT	chr6:59772079:+	73	50	23	79	53	26	chr6:56831273	12.94	chr6:53530806-65742462	*cis*	I
F1LUN1_RAT	chr6:59772113:+	92	65	27	94	68	27	chr6:56831273	14.72	chr6:53530806-65742462	*cis*	I
F1LUN1_RAT	chr6:59772118:+	89	61	28	95	64	31	chr6:56831273	12.49	chr6:53530806-65742462	*cis*	I
F1LUN1_RAT	chr6:59772129:+	87	63	24	93	69	24	chr6:56831273	11.81	chr6:53530806-65742462	*cis*	I
F1LUN1_RAT	chr6:59772153:+	90	80	9	91	74	16	chr6:56831273	6.80	chr6:53530806-69173770	*cis*	I
***F1LUN1_RAT* average**	**chr6:59772063-59772192:+**				**90**	**66**	**25**	**chr6:56831273**	**14.46**	**chr6:53530806-65742462**	***cis***	I
Map4k5	chr6:91976035:+	nd	nd	nd	35	63	-28	chr6:86088639	18.59	chr6:80603661-92298609	*cis*	S
Map4k5	chr6:91976050:+	nd	nd	nd	15	52	-37	chr6:86088639	22.47	chr6:80603661-92298609	*cis*	S
Map4k5	chr6:91976119:+	17	56	-38	22	77	-55	chr6:86088639	12.15	chr6:80603661-92298609	*cis*	S
***Map4k5* average**	**chr6:91975954-91976205:+**				**24**	**64**	**-40**	**chr6:86088639**	**23.28**	**chr6:80603661-92298609**	***cis***	S
Snapc1	chr6:96511559:+	10	21	-12	11	23	-12	chr6:93310927	4.54	chr6:86088639-104870403	*cis*	I
Snapc1	chr6:96511617:+	5	12	-7	9	17	-8	chr14:13776720	2.61	n/a	no signif linkage	I
Snapc1	chr6:96511637:+	7	29	-22	9	25	-16	chr6:93310927	4.45	chr6:80603661-104870403	*cis*	I
Snapc1	chr6:96511668:+	25	39	-14	31	57	-26	chr6:93310927	9.39	chr6:92298609-97966119	*cis*	I
***Snapc1* average**	**chr6:96511512-96511670:+**				**15**	**30**	**-16**	**chr6:93310927**	**9.64**	**chr6:92298609-98679608**	***cis***	I
F1M804_RAT	chr6:98659102:+	55	64	-9	44	45	-1	chr2:242410458	3.25	n/a	no signif linkage	I
F1M804_RAT	chr6:98659149:+	83	78	5	70	63	6	chr6:6778779	2.67	n/a	no signif linkage	I
F1M804_RAT	chr6:98659194:+	85	38	46	77	23	55	chr6:98679608	18.50	chr6:97966119-98698763	*cis*	I
F1M804_RAT	chr6:98659199:+	75	82	-7	68	64	4	chr3:103249862	2.83	n/a	no signif linkage	I
***F1M804_RAT* average**	**chr6:98659081-98659233:+**				**65**	**49**	**16**	**chr6:98679608**	**5.17**	**chr6:93310927-104870403**	***cis***	I
Bcl11b	chr6:132185613:+	50	74	-24	46	61	-14	chr6:131897632	4.23	chr6:129241985-144480901	*cis*	I
Bcl11b	chr6:132185695:+	20	52	-32	14	42	-28	chr6:131897632	10.39	chr6:129241985-132699487	*cis*	I
Bcl11b	chr6:132185717:+	29	56	-28	32	49	-17	chr6:131897632	3.31	n/a	no signif linkage	I
***Bcl11b* average**	**chr6:132185611-132185763:+**				**31**	**51**	**-20**	**chr6:131897632**	**10.36**	**chr6:129241985-132699487**	***cis***	I
Ppp1r13b	chr6:136693323:+	44	70	-26	37	69	-31	chr6:132699487	9.63	chr6:129241985-144480901	*cis*	I
Ppp1r13b	chr6:136693329:+	83	74	9	70	62	8	chr6:132699487	5.15	chr6:129241985-144480901	*cis*	I
Ppp1r13b	chr6:136693343:+	38	31	7	36	25	12	chr6:132699487	7.25	chr6:131897632-141539352	*cis*	I
Ppp1r13b	chr6:136693363:+	70	61	9	69	52	17	chr6:132699487	5.13	chr6:128853105-145193067	*cis*	I
Ppp1r13b	chr6:136693388:+	40	40	0	37	33	3	chr6:132699487	5.14	chr6:129241985-144480901	*cis*	I
Ppp1r13b	chr6:136693396:+	85	86	-1	76	76	0	chr19:2708374	2.30	n/a	no signif linkage	I
Ppp1r13b	chr6:136693419:+	87	91	-4	82	82	0	chr16:45349355	3.15	n/a	no signif linkage	I
Ppp1r13b	chr6:136693436:+	80	76	4	76	76	0	chr1:90431156	2.45	n/a	no signif linkage	I
***Ppp1r13b* average**	**chr6:136693300-136693442:+**				**60**	**59**	**1**	**chr6:132699487**	**3.34**	**n/a**	***no signif linkage***	I
Fgd6	chr7:31169450:+	91	84	7	91	84	7	chr7:30134941	3.89	n/a	no signif linkage	I
Fgd6	chr7:31169505:+	88	77	11	73	64	9	chr7:30134941	4.19	chr7:22107933-32221040	*cis*	I
Fgd6	chr7:31169556:+	70	48	21	66	57	10	chr7:30134941	5.40	chr7:22107933-32221040	*cis*	I
***Fgd6* average**	**chr7:31169443-31169556:+**				**77**	**68**	**9**	**chr7:30134941**	**6.46**	**chr7:22107933-32221040**	***cis***	I

Tbc1d30	chr7:60595192:+	31	27	4	40	33	8	chr7:60397471	5.50	chr7:60128525-72623825	*cis*	I
Tbc1d30	chr7:60595202:+	35	36	-1	43	39	5	chr2:153116666	3.01	n/a	no signif linkage	I
Tbc1d30	chr7:60595204:+	20	25	-5	27	34	-7	chr6:9974757	2.62	n/a	no signif linkage	I
Tbc1d30	chr7:60595224:+	24	26	-2	20	33	-13	chr2:149178695	2.06	n/a	no signif linkage	I
Tbc1d30	chr7:60595233:+	32	1	32	38	29	9	chr2:159881070	3.19	n/a	no signif linkage	I
Tbc1d30	chr7:60595253:+	40	35	5	43	62	-20	chr7:60128525	6.55	chr7:51368543-61371130	*cis*	I
Tbc1d30	chr7:60595274:+	40	34	6	45	60	-15	chr7:60128525	8.07	chr7:51368543-61371130	*cis*	I
Tbc1d30	chr7:60595314:+	32	24	8	41	48	-7	chr7:60128525	3.56	n/a	no signif linkage	I
Tbc1d30	chr7:60595335:+	14	10	4	22	26	-4	chr3:164374674	2.33	n/a	no signif linkage	I
Tbc1d30	chr7:60595351:+	4	6	-2	13	13	0	chr19:43368611	2.23	n/a	no signif linkage	I
***Tbc1d30* average**	**chr7:60595191-60595353:+**				**33**	**38**	**-4**	**chr7:41970847**	**2.95**	**n/a**	***no signif linkage***	I
Nsmce2	chr7:96296512:+	92	89	3	73	60	13	chr15:94925958	2.43	n/a	no signif linkage	I
Nsmce2	chr7:96296572:+	84	88	-4	90	78	13	chr7:94054636	3.58	n/a	no signif linkage	I
Nsmce2	chr7:96296599:+	58	35	23	50	31	19	chr7:94054636	10.32	chr7:92129202-98093741	*cis*	I
Nsmce2	chr7:96296618:+	94	78	16	92	91	1	chr20:34084707	2.29	n/a	no signif linkage	I
***Nsmce2* average**	**chr7:96296486-96296639:+**				**76**	**65**	**11**	**chr7:94054636**	**9.49**	**chr7:92129202-98093741**	***cis***	I
Ly6e	chr7:113120800:+	2	77	-75	11	70	-59	chr7:113865618	7.37	chr7:106541122-114437124	*cis*	S
Ly6e	chr7:113120876:+	19	44	-25	16	62	-45	chr7:108819248	8.08	chr7:106541122-113865618	*cis*	S
Ly6e	chr7:113120938:+	2	15	-13	0	49	-49	chr7:108819248	9.00	chr7:106541122-113865618	*cis*	S
***Ly6e* average**	**chr7: 113120765-113121068:+**				**9**	**60**	**-51**	**chr7:108819248**	**9.61**	**chr7:106541122-114437124**	***cis***	S
Naprt1	chr7:113868517:-	5	29	-24	14	53	-39	chr7:113865618	4.39	chr7:106541122-114437124	*cis*	S
Naprt1	chr7:113868460:-	2	42	-40	10	53	-43	chr7:113865618	12.68	chr7:108819248-114437124	*cis*	S
Naprt1	chr7:113868451:-	11	30	-19	16	70	-55	chr7:113865618	12.98	chr7:108819248-114437124	*cis*	S
Naprt1	chr7:113868449:-	7	23	-16	10	54	-44	chr7:113865618	12.55	chr7:108819248-114437124	*cis*	S
Naprt1	chr7:113868442:-	13	34	-21	14	67	-53	chr7:113865618	10.16	chr7:108819248-114437124	*cis*	S
Naprt1	chr7:113868436:-	15	32	-17	36	75	-39	chr7:113865618	9.77	chr7:108819248-114437124	*cis*	S
***Naprt1* avearage**	**chr7:113868322-113868588:-**				**17**	**62**	**-46**	**chr7:113865618**	**9.77**	**chr7:108819248-114437124**	***cis***	S
E9PSL5_RAT	chr7:116060485:+	28	53	-25	29	52	-24	chr7:114437124	8.35	chr7:113865618-122635137	*cis*	I
E9PSL5_RAT	chr7:116060555:+	66	78	-12	58	72	-14	chr7:117007096	6.44	chr7:108819248-125417420	*cis*	I
E9PSL5_RAT	chr7:116060590:+	86	91	-5	86	87	-1	chr5:141408867	2.50	n/a	no signif linkage	I
E9PSL5_RAT	chr7:116060597:+	79	78	1	74	85	-11	chr9:4289268	3.25	n/a	no signif linkage	I
***E9PSL5_RAT* average**	**chr7:116060462-116060604:+**				**62**	**74**	**-12**	**chr7:114437124**	**7.76**	**chr7:108819248-121883064**	***cis***	I
Nfam1	chr7:121018647:+	86	77	9	77	64	13	chr7:114437124	4.48	chr7:100067811-128152751	*cis*	I
Nfam1	chr7:121018653:+	87	83	4	73	60	14	chr7:117555752	2.60	n/a	no signif linkage	I
Nfam1	chr7:121018720:+	60	33	27	64	37	27	chr7:117555752	10.13	chr7:117007096-121842583	*cis*	I
Nfam1	chr7:121018752:+	75	46	29	66	51	15	chr7:117555752	7.10	chr7:117007096-121883510	*cis*	I
Nfam1	chr7:121018772:+	80	55	25	84	53	31	chr7:117555752	13.11	chr7:117007096-121842583	*cis*	I
***Nfam1* average**	**chr7:121018632-121018780:+**				**73**	**53**	**20**	**chr7:117555752**	**13.84**	**chr7:117007096-121711167**	***cis***	I
F1M9Y3_RAT	chr7:13508204:+	2	23	-21	2	32	-30	chr7:10417532	16.97	chr7:10072378-13886210	*cis*	I
F1M9Y3_RAT	chr7:13508226:+	2	15	-13	1	27	-26	chr7:10417532	11.99	chr7:10072378-13886210	*cis*	I
F1M9Y3_RAT	chr7:13508243:+	2	21	-19	3	33	-30	chr7:10417532	13.50	chr7:10072378-13886210	*cis*	I
F1M9Y3_RAT	chr7:13508247:+	3	27	-24	3	39	-37	chr7:10417532	16.33	chr7:10072378-13886210	*cis*	I
F1M9Y3_RAT	chr7:13508251:+	2	23	-21	3	35	-31	chr7:10417532	14.45	chr7:10072378-13886210	*cis*	I
F1M9Y3_RAT	chr7:13508299:+	4	29	-25	2	35	-34	chr7:10417532	14.94	chr7:10072378-13886210	*cis*	I
***F1M9Y3_RAT* average**	**chr7:13508194-13508311:+**				**2**	**33**	**-31**	**chr7:10417532**	**15.70**	**chr7:10072378-13886210**	***cis***	I
LOC100365068	chr7:135196747:+	81	57	24	84	61	24	chr7:135435079	8.35	chr7:134894183-139323661	*cis*	I
LOC100365068	chr7:135196761:+	91	60	31	80	58	23	chr7:135435079	11.33	chr7:134894183-139323661	*cis*	I
LOC100365068	chr7:135196785:+	93	65	28	88	62	26	chr7:135435079	10.83	chr7:134894183-139323661	*cis*	I
LOC100365068	chr7:135196787:+	92	65	27	87	63	24	chr7:135435079	9.76	chr7:134894183-139323661	*cis*	I

LOC100365068	chr7:135196791:+	89	62	27	84	55	29	chr7:135435079	10.02	chr7:134894183-139323661	*cis*	I
LOC100365068	chr7:135196795:+	90	66	25	89	63	27	chr7:135435079	11.37	chr7:134894183-139323661	*cis*	I
LOC100365068	chr7:135196802:+	86	60	26	82	59	22	chr7:135435079	10.80	chr7:134894183-139323661	*cis*	I
LOC100365068	chr7:135196840:+	71	44	28	62	48	14	chr7:135435079	5.63	chr7:134894183-139323661	*cis*	I
LOC100365068	chr7:135196887:+	75	45	30	69	54	15	chr7:135435079	8.48	chr7:134894183-139323661	*cis*	I
LOC100365068	chr7:135196895:+	77	51	26	81	55	25	chr7:135435079	9.42	chr7:134894183-139323661	*cis*	I
***LOC100365068* average**	**chr7:135196738-135196895:+**				**81**	**58**	**23**	**chr7:135435079**	**10.79**	**chr7:134894183-139323661**	***cis***	I
Bl1_RAT	chr7:138157743:+	35	76	-41	14	28	-15	chr4:115063428	3.64	n/a	no signif linkage	I
Bl1_RAT	chr7:138157753:+	22	22	22	18	33	-15	chr1:205671704	2.15	n/a	no signif linkage	I
Bl1_RAT	chr7:138157862:+	5	8	-3	9	19	-10	chr2:256707794	1.66	n/a	no signif linkage	I
Bl1_RAT	chr7:138157866:+	6	11	-5	11	24	-13	chr9:67866953	3.57	n/a	no signif linkage	I
***Bl1_RAT* average**	**chr7:138157743-138157866:+**				**13**	**26**	**-13**	**chr6:37984850**	**4.08**	**n/a**	***no signif linkage***	I
Krt4	chr7:140634138:-	86	36	50	95	81	14	chr7:141354287	11.03	chr7:135435079-143002779	*cis*	S
Krt4	chr7:140634155:-	88	33	55	94	76	19	chr7:141354287	11.58	chr7:139538952-141594453	*cis*	S
Krt4	chr7:140634165:-	90	39	51	95	79	16	chr7:141354287	13.65	chr7:139538952-141594453	*cis*	S
Krt4	chr7:140634192:-	82	27	55	86	53	33	chr7:141354287	13.33	chr7:139538952-143002779	*cis*	S
Krt4	chr7:140634198:-	73	21	52	87	51	36	chr7:141354287	14.16	chr7:139538952-141594453	*cis*	S
Krt4	chr7:140634213:-	73	30	43	88	60	27	chr7:141354287	13.16	chr7:139538952-143002779	*cis*	S
Krt4	chr7:140634215:-	70	34	36	89	66	23	chr7:141354287	14.32	chr7:139538952-141594453	*cis*	S
Krt4	chr7:140634224:-	74	25	49	84	56	27	chr7:141354287	12.02	chr7:139538952-143002779	*cis*	S
Krt4	chr7:140634256:-	81	27	54	84	53	31	chr7:141354287	16.44	chr7:139538952-141594453	*cis*	S
Krt4	chr7:140634295:-	93	81	12	99	92	6	chr7:141354287	7.12	chr7:134894183-143002779	*cis*	S
Krt4	chr7:140634316:-	88	59	29	90	77	13	chr7:141354287	13.50	chr7:139538952-141594453	*cis*	S
Krt4	chr7:140634324:-	87	61	26	84	73	11	chr7:141354287	11.97	chr7:139538952-143002779	*cis*	S
***Krt4* average**	**chr7:140634112-140634395:-**				**89**	**68**	**21**	**chr7:141354287**	**14.62**	**chr7:139538952-141594453**	***cis***	S
Ccdc96	chr14:79858821:-	66	7	59	68	27	41	chr14:78866840, chr14:80263250, chr14:83340812	12.12	chr14:77232950-84045063	*cis*	S
Ccdc96	chr14:79858801:-	73	7	66	80	29	51	chr14:78866840, chr14:80263250, chr14:83340812	14.47	chr14:77232950-84045063	*cis*	S
Ccdc96	chr14:79858798:-	71	8	63	78	31	47	chr14:78866840, chr14:80263250, chr14:83340812	13.33	chr14:77232950-84045063	*cis*	S
Ccdc96	chr14:79858790:-	76	6	70	83	29	54	chr14:78866840, chr14:80263250, chr14:83340812	14.59	chr14:77232950-84045063	*cis*	S
Ccdc96	chr14:79858766:-	65	5	60	81	30	51	chr14:78866840, chr14:80263250, chr14:83340812	14.49	chr14:77232950-84045063	*cis*	S
***Ccdc96* average**	**chr14:79858556-79858886:-**				**78**	**29**	**49**	**chr14:78866840, chr14:80263250, chr14:83340812**	**14.21**	**chr14:77232950-84045063**	***cis***	S
Ddhd1	chr15:21569719:-	58	80	-22	79	87	-8	chr15:17009597	3.08	n/a	no signif linkage	S
Ddhd1	chr15:21569766:-	26	59	-33	47	74	-28	chr15:21493194	10.84	chr15:21046469-22822009	*cis*	S
Ddhd1	chr15:21569816:-	48	66	-18	53	63	-10	chr15:21493194	3.79	n/a	no signif linkage	S
Ddhd1	chr15:21569826:-	52	74	-22	53	68	-15	chr15:21493194	6.14	chr15:21046469-22822009	*cis*	S
Ddhd1	chr15:21569898:-	61	74	-13	68	74	-7	chr7:117555752	1.82	n/a	no signif linkage	S
***Ddhd1* average**	**chr15:21569685-21570002:-**				**50**	**71**	**-21**	**chr15:21493194**	**10.37**	**chr15:21046469-22822009**	***cis***	S
Cbln1	chr19:20944468:+	13	57	-44	20	63	-43	chr19:21144074	8.74	chr19:15280687-29813557	*cis*	S
Cbln1	chr19:20944480:+	17	33	-16	23	66	-43	chr19:16528440, chr19:21481304	9.93	chr19:15280687-26511523	*cis*	S

Cbln1	chr19:20944486:+	13	80	-67	29	72	-43	chr19:21144074	8.52	chr19:15280687-29813557	*cis*	S
Cbln1	chr19:20944494:+	26	37	-11	24	67	-44	chr19:21144074	10.29	chr19:15280687-26511523	*cis*	S
Cbln1	chr19:20944514:+	10	37	-27	18	64	-46	chr19:21144074	10.94	chr19:15280687-26511523	*cis*	S
Cbln1	chr19:20944536:+	17	64	-47	24	67	-43	chr19:21144074	10.22	chr19:15280687-26511523	*cis*	S
Cbln1	chr19:20944576:+	9	20	-11	3	27	-24	chr19:21144074	16.22	chr19:15280687-26511523	*cis*	S
Cbln1	chr19:20944624:+	0	34	-34	15	58	-43	chr19:21144074	11.15	chr19:15280687-26511523	*cis*	S
***Cbln1* average**	**chr19:20944355-20944663:+**				**19**	**61**	**-41**	**chr19:21144074**	**11.50**	**chr19:15280687-26511523**	***cis***	S
Grm4	chr20:5655482:-	62	39	23	89	73	16	chr20:2931904	14.20	chr20:0-6999342	*cis*	S
Grm4	chr20:5655411:-	43	19	24	73	49	24	chr20:2931904	7.37	chr20:0-6999342	*cis*	S
Grm4	chr20:5655375:-	32	12	20	77	40	37	chr20:2931904	12.75	chr20:0-6999342	*cis*	S
***Grm4* average**	**chr20:5655243-5655528:-**				**80**	**54**	**26**	**chr20:2931904**	**13.57**	**chr20:0-6999342**	***cis***	S
Rtdr1	chr20:14187239:+	9	9	9	11	18	-8	chr5:126790264	2.38	n/a	no signif linkage	I
Rtdr1	chr20:14187266:+	8	44	-36	5	7	-2	chr17:70462220	2.09	n/a	no signif linkage	I
Rtdr1	chr20:14187282:+	8	41	-33	9	9	0	chr6:52768463	2.58	n/a	no signif linkage	I
Rtdr1	chr20:14187284:+	9	42	-33	8	11	-3	chr9:53822255	2.34	n/a	no signif linkage	I
Rtdr1	chr20:14187310:+	11	21	-10	8	7	1	chr6:52768463	2.93	n/a	no signif linkage	I
Rtdr1	chr20:14187345:+	7	17	-10	11	13	-2	chr19:47704959	1.90	n/a	no signif linkage	I
Rtdr1	chr20:14187371:+	17	24	-7	17	18	-2	chr9:10303064	3.04	n/a	no signif linkage	I
***Rtdr1* average**	**chr20:14187231-14187390:+**				**10**	**12**	**-2**	**chr6:50926188**	**1.87**	n/a	**no signif linkage**	I

a) Name of the gene closest to the measured CpGs,
b) WGBS = whole genome bisulfite sequencing, methylation percentage in BN and SHR strains, or difference between the strains (BN-SHR),
c) BN, SHR and difference indicate percentages and difference in BN and SHR methylation (BN-SHR) determined by Illumina or Sanger sequencing,
d) multiple peak of linkage coordinates are listed when they all have the same LOD score,
e) LOD score indicates the LOD score at the peak of linkage, for individual CpG dinucleotides and for average CpG methylation across the entire amplicon,
f) linkage data was generated by conventional PCR and Sanger sequencing (S) or by amplification using the Fluidigm platform followed by Illumina sequencing (I),
g) nd = no data in the WGBS data set of 10,614,445 sampled CpG dinucleotides,
h) n/a = not applicable,
i) no significant linkage, defined as P>0.05 after a minimum of 1000 permutations of the linkage data
Bold type indicates the average methylation data for each amplicon
