# Supplementary material for: Genetic Analysis of the Cardiac Methylome at Single Nucleotide Resolution in a Model of Human Cardiovascular Disease
Source: PLoS Genet. 2014 Dec 4;10(12):e1004813. doi: 10.1371/journal.pgen.1004813 (PMC4256262; doi:10.1371/journal.pgen.1004813)
Supplement: Table S4 — Trans meth-QTL interval genes. (PDF) [file pgen.1004813.s017.pdf]

Table S4 *Trans* meth-QTL interval genes

Odfp2

Gene type	Number in 2-LOD interval	Number detected in RNA-seq data set	Number that are differentially expressed	Number containing non-synonymous variants or SNPs[a]
microRNAs	0	n/a[b]	n/a	n/a
miscellanous RNAs	2	No reads obtained	n/a	1
protein coding genes	7	7	3	4
pseudogenes	1	1	0	0
rRNAs	0	n/a	n/a	n/a
snoRNAs	2	No reads obtained	n/a	0
snRNAs	1	No reads obtained	n/a	1
**Total**	**13**	**8**	**3**	**6**

Asap2

Gene type	Number in 2-LOD interval	Number detected in RNA-seq data set	Number that are differentially expressed	Number containing non-synonymous variants or SNPs[a]
microRNAs	4	2	1	2
miscellanous RNAs	2	No reads obtained	n/a	2
protein coding genes	157	131	64	44
pseudogenes	2	1	0	0
rRNAs	4	2	0	0
snoRNAs	4	2	0	1
snRNAs	10	1	0	0
**Total**	**183**	**139**	**65**	**49**

a) Non-synonymous variants in protein coding genes, (SNPs) Single nucleotide polymorphisms in non-protein coding genes.

b) (n/a) not available
