# Supplementary material for: Genetic Analysis of the Cardiac Methylome at Single Nucleotide Resolution in a Model of Human Cardiovascular Disease
Source: PLoS Genet. 2014 Dec 4;10(12):e1004813. doi: 10.1371/journal.pgen.1004813 (PMC4256262; doi:10.1371/journal.pgen.1004813)
Supplement: Table S5 — Read mapping statistics for RNA-seq reads generated in BN and SHR rats. (PDF) [file pgen.1004813.s018.pdf]

Table S5 Read mapping statistics for RNA-seq reads generated in BN and SHR rats

Sample	Total Reads	Mapped Reads	[%]	Detected Exons	[%]	Detected Genes	[%]
BN5	87,000,686	56,508,044	65	170,778	71	18,698	63
BN6	85,210,074	54,082,224	63	169,036	70	18,425	62
BN7	73,689,476	45,607,749	62	173,223	72	18,900	64
BN8	62,604,648	39,425,162	63	167,558	69	18,155	62
**BN total**	**308,504,884**	**195,623,179**	**63**	**186,194**	**77**	**20,425**	**69**
SHR5	74,538,102	46,332,951	62	170,851	71	18,390	62
SHR6	85,211,922	53,195,586	62	172,716	71	18,586	63
SHR7	65,158,566	40,842,670	63	168,057	70	18,101	61
SHR8	80,868,854	51,062,549	63	172,903	72	18,599	63
**SHR total**	**305,777,444**	**191,433,756**	**63**	**186,133**	**77**	**20,281**	**69**

Summary of the number of reads obtained for each sample and the percentage of reads mapped to the genome sequence (RGSC3.4 reference assembly) and the number and percentage of exons/genes (Ensembl 62) detected by mapped reads.
