# Supplementary material for: Genetic Analysis of the Cardiac Methylome at Single Nucleotide Resolution in a Model of Human Cardiovascular Disease
Source: PLoS Genet. 2014 Dec 4;10(12):e1004813. doi: 10.1371/journal.pgen.1004813 (PMC4256262; doi:10.1371/journal.pgen.1004813)
Supplement: Table S6 — Summary of the results of a two-way ANOVA to test for independence of methylation levels and the two factors strain and cell/tissue type. (PDF) [file pgen.1004813.s019.pdf]

Table S6 Summary of the results of a two-way ANOVA to test for independence of methylation levels and the two factors strain and cell/tissue type

Amplicon Name	Amplicon Coordinates	CpGs Measured	P(strain) [a]		P(tissue) [a]	
Akr1b10	chr4:61757621-61757753:+	6	4.35E-03	*	6.78E-06	***
Asap2	chr6:41745647-41745769:+	4	5.14E-02	*	9.51E-01	-
Bcl11b	chr6:132185611-132185763:+	3	4.38E-03	*	9.12E-01	-
Bl1_RAT	chr7:138157743-138157866:+	4	2.64E-02	*	8.06E-04	**
Cdkn2b	chr5:108937522-108937640:+	5	2.13E-01	-	5.97E-01	-
E9PSL5_RAT	chr7:116060462-116060604:+	4	2.36E-01	-	9.94E-01	-
F1LUN1_RAT	chr6:59772063-59772192:+	5	1.02E-09	***	9.47E-02	*
F1M4B7_RAT	chr5:87563005-87563150:+	4	7.05E-07	***	2.24E-01	-
F1M804_RAT	chr6:98659081-98659233:+	5	3.26E-01	-	9.08E-01	-
F1M9Y3_RAT	chr7:13508194-13508311:+	7	6.17E-29	***	1.80E-03	*
Fgd6	chr7:31169443-31169556:+	3	1.56E-01	-	9.05E-01	-
Krt4	chr7:140634112-140634395:+	8	2.17E-36	***	2.67E-01	-
LOC100365068	chr7:135196738-135196895:+	10	7.49E-18	***	3.73E-01	-
Nfam1	chr7:121018632-121018780:+	6	1.60E-04	**	7.77E-01	-
Nsmaf	chr5:19887357-19887486:+	4	5.51E-03	*	8.92E-01	-
Nsmce2	chr7:96296486-96296639:+	5	3.64E-02	*	9.98E-01	-
Ppp1r13b	chr6:136693300-136693442:+	8	1.00E+00	-	9.85E-01	-
RGD1564053	chr5:6849814-6849947:+	4	1.20E-01	-	9.40E-01	-
RGD1565350	chr5:62118077-62118218:+	10	9.39E-01	-	1.81E-01	-
Rragc	chr5:143257694-143257854:+	5	7.76E-05	***	3.98E-01	-
Rragc_2	chr5:143573578-143573701:+	5	9.40E-06	***	7.85E-01	-
Rtdr1	chr20:14187231-14187390:+	7	2.16E-02	*	3.13E-02	*
Slc2a1	chr5:139703426-139703584:+	4	4.59E-02	*	9.44E-01	-
Snapc1	chr6:96511512-96511670:+	4	1.39E-02	*	9.07E-01	-
Tbc1d30	chr7:60595191-60595353:+	10	8.89E-01	-	7.31E-03	*
Tmem178	chr6:3892290-3892428:+	7	1.49E-03	*	2.12E-01	-

a) Significance codes: '***' P< 0.0001, '**' P < 0.001, '*' P < 0.05, '.' P < 0.1, '-' P >= 0.1.
