# Supplementary material for: Genetic Analysis of the Cardiac Methylome at Single Nucleotide Resolution in a Model of Human Cardiovascular Disease
Source: PLoS Genet. 2014 Dec 4;10(12):e1004813. doi: 10.1371/journal.pgen.1004813 (PMC4256262; doi:10.1371/journal.pgen.1004813)
Supplement: Table S7 — Comparison of position frequency matrices of nucleotides associated with hyper- and hypomethylated CpGs in rat and mouse. (PDF) [file pgen.1004813.s020.pdf]

Table S7 Comparison of position frequency matrices of nucleotides associated with hyper- and hypomethylated CpGs in rat and mouse

Query Matrix	Target Matrix	Query Consensus	Target Consensus	P-value
Rat Hypomethylated	Mouse Hypometylated	ATACACGTGTAT	AAACACGTGTAT	2.76E-10
Rat Hypermethylated	Mouse Hypermethylated	GCGTGCGCACGC	GCGTGCGCACGC	1.06E-10
Rat Hypomethylated	Mouse Hypermethylated	ATACACGTGTAT	GCGTGCGCACGC	7.65E-01
Rat Hypermethylated	Mouse Hypometylated	GCGTGCGCACGC	AAACACGTGTAT	9.48E-01
