# Supplementary material for: Genetic Analysis of the Cardiac Methylome at Single Nucleotide Resolution in a Model of Human Cardiovascular Disease
Source: PLoS Genet. 2014 Dec 4;10(12):e1004813. doi: 10.1371/journal.pgen.1004813 (PMC4256262; doi:10.1371/journal.pgen.1004813)
Supplement: Table S8 — Mapping and filtering of whole genome bisulfite sequencing reads generated in reciprocal F1 crosses. (PDF) [file pgen.1004813.s021.pdf]

Table S8 Mapping and filtering of whole genome bisulfite sequencing reads generated in reciprocal F1 crosses

Sample	Total Reads	Mapped Reads		Mapped Bases	Depth of Coverage	Mapped Bases After Filtering	Depth of Coverage After Filtering
	[millions]	[millions]	[%]	[billions]	[x-fold]	[billions]	[x-fold]
BNxSHR1	750	695	93	63.2	24.6	43.5	16.9
BNxSHR2	726	672	93	62.4	24.3	43.1	16.8
BNxSHR3	662	616	93	57.4	22.3	39.2	15.2
BNxSHR4	733	680	93	63.0	24.5	42.5	16.5
**BNxSHR total**	**2,871**	**2,662**	**93**	**245.9**	**95.6**	**168.2**	**65.4**
SHRxBN1	916	843	92	76.6	29.8	50.5	19.6
SHRxBN2	641	587	92	54.6	21.2	36.3	14.1
SHRxBN3	902	819	91	72.3	28.1	47.8	18.6
SHRxBN4	1,077	976	91	87.6	34.1	58.0	22.6
**SHRxBN total**	**3,536**	**3,225**	**91**	**291.1**	**113.2**	**192.6**	**74.9**

Summary of the number of reads obtained for each sample, the proportion of reads mapped to the bisulfite-converted genome and the depth of coverage of the genome before and after quality filtering of the data set. The genome is defined as the mappable (ungapped) sequence of the RGSC3.4 reference assembly (total length=2,571,531,505bp).
