# Supplementary material for: Genetic Analysis of the Cardiac Methylome at Single Nucleotide Resolution in a Model of Human Cardiovascular Disease
Source: PLoS Genet. 2014 Dec 4;10(12):e1004813. doi: 10.1371/journal.pgen.1004813 (PMC4256262; doi:10.1371/journal.pgen.1004813)
Supplement: Table S9 — Summary of genomic regions showing parent-of-origin specific differences in methylation. (PDF) [file pgen.1004813.s022.pdf]

Table S9 Summary of genomic regions showing parent-of-origin specific differences in methylation[a]

#	Chr	Start [bp]	End [bp]	Region Size [bp][b]	CGI[c]	TSS[d]	Exonic	Intronic	Intergenic[e]	Gene ID	Gene Name	CpGs In Region	CpGs Analysed				CpGs Significantly Differentially Methylated				Mean Methylation Difference Across Region [%]				Max. Mean Methylation Difference [%]	Allele Showing Increased Methylation[f]			
													BN Chromosomes		SHR Chromosomes		BN Chromosomes		SHR Chromosomes		BN Chromosomes		SHR Chromosomes			BN Chromosomes		SHR Chromosomes	
													Forward Strand	Reverse Strand	Forward Strand	Reverse Strand	Forward Strand	Reverse Strand	Forward Strand	Reverse Strand	Forward Strand	Reverse Strand	Forward Strand	Reverse Strand		Forward Strand	Reverse Strand	Forward Strand	Reverse Strand
1	1	7,882,907	7,883,212	306	+	+		+		ENSRNOG00000025587	*Plagl1*	30	0	0	28	0	0	0	28	0	-	-	98	-	98	-	-	M	-
2	3	165,214,162	165,214,636	475	+	+	+			ENSRNOG00000025889	*GNAS3_RAT*	34	27	27	0	0	27	16	0	0	97	97	-	-	97	P	P	-	-
3	1	202,827,298	202,827,575	278	+				+	-	-	16	10	10	0	13	5	10	0	13	94	68	-	51	94	P	P	-	P
4	14	92,910,245	92,910,575	331	+	+		+		ENSRNOG00000004290	*Grb10*	39	15	15	38	38	14	5	33	38	84	83	89	93	93	M	M	M	M
5	7	110,540,679	110,542,188	1,510	+			+		ENSRNOG00000027569	*Trappc9*	74	2	2	42	35	2	0	38	0	92	-	89	6	92	M	-	M	M
6	1	202,828,927	202,829,773	847	+				+	-	-	34	24	24	13	0	22	0	11	0	88	91	79	-	91	P	P	P	-
7	7	131,720,145	131,720,145	1					+	-	-	1	0	0	0	1	0	0	0	1	-	-	-	86	86	-	-	-	P
8	7	110,542,698	110,542,705	8	+			+		ENSRNOG00000027569	*Trappc9*	2	0	0	2	0	0	0	2	0	-	-	86	-	86	-	-	M	-
9	1	202,828,176	202,828,269	94	+				+	-	-	7	7	7	4	0	7	0	4	0	85	-	81	-	85	P	-	P	-
10	6	134,118,057	134,118,457	401	+			+		NR_027652	*Meg3*	15	15	15	6	0	15	0	6	0	80	-	67	-	80	P	-	P	-
11	9	61,924,058	61,924,152	95					+	-	-	5	5	5	4	0	5	0	4	0	76	-	76	-	76	P	-	P	-
12	9	61,942,510	61,943,368	859	+				+	-	-	17	4	6	0	3	2	6	0	3	64	37	-	73	73	P	P	-	P
13	8	94,777,428	94,777,580	153					+	-	-	7	7	7	2	0	6	0	0	0	72	-	40	-	72	P	-	P	-
14	2	144,587,430	144,587,430	1					+	-	-	1	1	1	1	0	1	0	0	0	70	-	12	-	70	P	-	P	-
15	14	103,657,709	103,657,709	1	+	+		+		ENSRNOG00000009281	*Commd1*	1	0	0	1	0	0	0	1	0	-	-	69	-	69	-	-	M	-
16	3	160,066,888	160,067,025	138					+	-	-	11	11	11	11	7	10	0	11	0	35	52	35	69	69	M	M	M	M
17	8	94,766,769	94,766,769	1					+	-	-	1	0	1	0	1	0	1	0	1	-	68	-	45	68	-	P	-	P
18	8	109,556,004	109,556,004	1					+	-	-	1	1	1	0	0	1	0	0	0	68	-	-	-	68	M	-	-	-
19	9	61,921,110	61,921,492	383					+	-	-	8	8	8	3	0	7	0	3	0	65	-	67	-	67	P	-	P	-
20	3	89,680,758	89,680,758	1					+	-	-	1	0	0	1	0	0	0	1	0	-	-	66	-	66	-	-	M	-
21	8	94,782,149	94,782,430	282					+	-	-	8	0	8	0	5	0	7	0	5	-	39	-	66	66	-	P	-	P
22	9	61,925,095	61,925,565	471					+	-	-	9	9	9	8	0	4	0	7	0	49	-	65	-	65	P	-	P	-
23	20	4,748,306	4,748,306	1					+	-	-	1	1	1	0	0	1	0	0	0	64	-	-	-	64	P	-	-	-
25	3	153,811,885	153,812,253	369	+	+	+	+		ENSRNOG00000007044	*L3mbtl*	15	6	6	15	9	6	3	9	0	46	64	44	32	64	M	M	M	M
27	1	202,825,223	202,825,540	318	+	+				ENSRNOG00000020367	*Mir675*	12	0	12	0	10	0	12	0	9	-	61	-	55	61	-	P	-	P
28	9	61,944,112	61,944,691	580					+	-	-	15	13	13	7	0	12	0	7	0	44	-	59	-	59	P	-	P	-
29	13	68,353,604	68,353,604	1				+		ENSRNOG00000002680	*Lamc1*	1	0	1	0	0	0	1	0	0	-	58	-	-	58	-	M	-	-
31	17	21,987,909	21,987,909	1	+				+	-	-	1	0	0	0	1	0	0	0	1	-	25	-	57	57	-	P	-	P
33	8	94,767,563	94,767,570	8					+	-	-	3	0	0	0	3	0	0	0	3	-	-	-	57	57	-	-	-	M
35	3	127,615,693	127,615,693	1					+	-	-	1	1	1	1	0	1	0	0	0	55	-	29	-	55	P	-	P	-
41	6	134,055,793	134,055,804	12					+	-	-	2	2	2	2	2	2	0	0	0	52	-	33	13	52	P	-	P	P
42	19	39,281,737	39,281,739	3	+				+	-	-	2	2	2	2	2	0	2	0	0	33	52	6	26	52	M	M	M	M
43	6	134,043,873	134,044,038	166	+				+	-	-	8	8	8	8	0	3	0	0	0	51	-	25	-	51	P	-	P	-
44	1	23,480,272	23,480,364	93	+				+	-	-	12	10	10	9	9	3	0	0	0	50	-	26	9	50	M	-	M	M
45	1	202,918,734	202,919,023	290	+				+	-	-	4	4	4	4	0	4	0	3	0	50	-	39	-	50	P	-	P	-
46	9	61,920,167	61,920,305	139					+	-	-	3	3	3	3	0	0	0	2	0	21	-	49	-	49	P	-	P	-
47	1	84,804,885	84,804,885	1			+			ENSRNOG00000042096	*Zfp30*	1	0	0	0	1	0	0	0	1	-	-	-	49	49	-	-	-	M
48	20	4,901,463	4,901,647	185					+	-	-	8	5	5	8	0	4	0	0	0	49	-	13	-	49	M	-	M	-
55	3	5,591,183	5,591,183	1				+		ENSRNOG00000032782	*Abo*	1	0	1	0	0	0	1	0	0	-	45	-	-	45	-	M	-	-
56	14	92,700,847	92,700,847	1				+		ENSRNOG00000004327	*Ddc*	1	1	1	1	0	1	0	0	0	45	-	17	-	45	M	-	M	-
60	14	93,055,005	93,055,005	1					+	-	-	1	1	1	1	0	0	0	1	0	43	-	44	-	44	M	-	M	-
61	4	65,562,379	65,562,395	17	+			+		ENSRNOG00000013378	*Svopl*	2	0	0	0	2	0	0	0	2	-	31	-	44	44	-	M	-	M
63	1	112,729,274	112,729,274	1					+	-	-	1	0	0	0	1	0	0	0	1	-	6	-	43	43	-	P	-	P
64	8	46,339,335	46,339,335	1					+	-	-	1	1	1	1	0	1	0	0	0	43	-	5	-	43	M	-	M	-
65	5	151,870,240	151,870,409	170	+		+	+		ENSRNOG00000008600	*Sytl1*	8	0	8	0	8	0	7	0	1	-	43	-	20	43	-	M	-	M
66	17	60,593,264	60,593,264	1					+	-	-	1	1	1	1	1	1	0	0	0	42	11	0	9	42	M	M	M	M
73	10	60,886,318	60,886,320	3				+		ENSRNOG00000029537	*Or1e1*	2	2	2	2	0	0	0	2	0	21	-	39	-	39	M	-	M	-
74	14	9,489,287	9,489,287	1	+				+	-	-	1	1	1	1	0	1	0	0	0	39	-	4	-	39	P	-	P	-
77	2	28,379,619	28,379,619	1					+	-	-	1	1	1	1	0	0	0	1	0	6	-	39	-	39	M	-	M	-
78	4	62,776,202	62,776,202	1					+	-	-	1	0	0	1	1	0	0	0	1	-	-	11	39	39	-	-	P	P
79	14	18,639,418	18,639,756	339			+	+		ENSRNOG00000021347	*Mthfd2l*	20	8	19	8	19	0	12	0	10	8	27	38	21	38	M	M	M	M
81	14	92,850,225	92,850,398	174			+	+		ENSRNOG00000004290	*Grb10*	10	10	10	10	0	9	0	7	0	38	-	36	-	38	P	-	P	-
84	19	12,443,227	12,443,227	1				+		ENSRNOG00000013742	*Large*	1	1	1	1	0	1	0	0	0	36	-	13	-	36	P	-	P	-
85	14	18,997,415	18,997,782	368	+			+		ENSRNOG00000042849	*Afm*	18	0	0	0	17	0	0	0	14	-	1	-	36	36	-	M	-	M
86	17	1,854,518	1,854,518	1					+	-	-	1	1	1	1	0	1	0	0	0	36	-	4	-	36	M	-	M	-
87	1	113,872,519	113,872,519	1					+	-	-	1	0	1	0	1	0	1	0	0	-	36	-	2	36	-	P	-	P
88	15	70,910,698	70,910,698	1					+	-	-	1	1	1	1	0	0	0	1	0	4	-	36	-	36	M	-	M	-
89	17	83,654,895	83,654,895	1					+	-	-	1	1	1	1	0	1	0	0	0	36	-	5	-	36	P	-	P	-
90	5	3,645,851	3,646,187	337	+				+	-	-	26	18	18	10	0	13	0	0	0	36	-	11	-	36	M	-	M	-
92	15	14,865,123	14,865,123	1	+			+		ENSRNOG00000009419	*Ptprg*	1	0	1	0	0	0	1	0	0	-	36	-	-	36	-	M	-	-
95	9	100,077,728	100,077,728	1					+	-	-	1	0	0	0	1	0	0	0	1	-	4	-	35	35	-	P	-	P
97	4	57,446,139	57,446,390	252					+	-	-	7	5	5	7	0	4	0	5	0	35	-	33	-	35	M	-	M	-
98	6	72,502,013	72,502,013	1					+	-	-	1	0	0	1	0	0	0	1	0	-	-	35	-	35	-	-	P	-
100	5	34,425,353	34,425,353	1				+		ENSRNOG00000024903	*Slc7a13*	1	0	1	0	1	0	1	0	0	-	34	-	14	34	-	P	-	P
102	6	18,047,863	18,047,863	1					+	-	-	1	1	1	1	0	1	0	0	0	34	-	18	-	34	M	-	M	-
103	1	113,973,893	113,973,893	1					+	-	-	1	0	0	0	1	0	0	0	1	-	26	-	34	34	-	P	-	P
104	1	80,412,253	80,412,253	1				+		ENSRNOG00000020381	*Pou2f2*	1	1	1	1	0	0	0	1	0	5	-	34	-	34	M	-	M	-
105	5	159,370,289	159,370,289	1					+	-	-	1	1	1	1	1	0	0	1	0	3	13	34	26	34	P	P	P	P
107	1	112,808,987	112,808,987	1		+				ENSRNOG00000043789	-	1	0	1	0	1	0	1	0	0	-	33	-	12	33	-	P	-	P
108	1	112,011,525	112,011,525	1					+	-	-	1	1	1	1	0	1	0	1	0	33	-	28	-	33	P	-	P	-
109	1	113,165,855	113,165,855	1					+	-	-	1	0	1	0	0	0	1	0	0	-	33	-	-	33	-	P	-	-
111	1	202,823,211	202,823,716	506		+				ENSRNOG00000020367	*Mir675*	11	8	8	8	0	4	0	4	0	27	-	33	-	33	P	-	P	-
113	19	43,658,111	43,658,111	1					+	-	-	1	1	1	1	0	1	0	0	0	33	-	8	-	33	P	-	P	-
114	14	93,053,137	93,053,137	1					+	-	-	1	1	1	1	0	0	0	1	0	25	-	33	-	33	M	-	M	-
115	5	89,259,455	89,259,455	1			+			ENSRNOG00000038621	*F1LVD6_RAT*	1	1	1	1	0	1	0	0	0	33	-	1	-	33	M	-	M	-
117	17	3,146,355	3,146,355	1					+	-	-	1	0	0	0	1	0	0	0	1	-	2	-	33	33	-	P	-	P
118	1	203,470,854	203,470,854	1				+		ENSRNOG00000020532	*Kcnq1*	1	1	1	1	0	1	0	0	0	33	-	15	-	33	M	-	M	-
119	3	115,321,521	115,321,521	1					+	-	-	1	0	0	0	1	0	0	0	1	-	13	-	33	33	-	P	-	P
120	1	248,550,642	248,550,642	1				+		ENSRNOG00000015220	*Crtac1*	1	1	1	0	1	0	1	0	0	9	33	-	6	33	M	M	-	M
124	1	46,587,282	46,587,520	239	+			+		ENSRNOG00000011310	*Pde10a*	8	8	8	8	1	2	1	0	0	29	32	12	30	32	M	M	M	M
127	1	113,495,921	113,495,921	1					+	-	-	1	1	1	1	0	1	0	0	0	24	-	31	-	31	P	-	P	-
136	7	12,666,822	12,666,822	1	+	+	+			ENSRNOG00000028146	*Olr1088*	1	1	1	1	1	0	0	1	0	15	-	30	4	30	M	-	M	M
139	1	111,629,546	111,629,546	1					+	-	-	1	1	1	1	0	0	0	1	0	9	-	30	-	30	P	-	P	-
144	3	107,815,513	107,815,513	1				+		ENSRNOG00000011649	*Epb42*	1	1	1	1	0	1	0	0	0	29	-	0	-	29	M	-	M	-
146	8	60,078,991	60,078,991	1				+		ENSRNOG00000016905	*Hmg20a*	1	1	1	1	0	0	0	1	0	3	-	29	-	29	P	-	P	-
147	5	141,638,078	141,638,285	208			+	+		ENSRNOG00000011502	*Col9a2*	9	9	9	9	0	4	0	3	0	29	18	25	-	29	M	M	M	-
151	1	116,709,605	116,709,605	1					+	-	-	1	0	1	0	1	0	1	0	0	-	29	-	20	29	-	P	-	P
152	8	104,337,045	104,337,045	1					+	-	-	1	0	1	0	0	0	1	0	0	-	28	-	-	28	-	M	-	-
156	20	44,874,459	44,874,465	7				+		ENSRNOG00000024221	*LOC499465*	3	3	3	3	0	3	0	0	0	28	-	15	-	28	M	-	M	-
157	1	251,621,669	251,621,669	1				+		ENSRNOG00000019807	*Sufu*	1	1	1	1	0	1	0	0	0	28	-	2	-	28	P	-	P	-
159	14	100,796,947	100,796,947	1	+			+		ENSRNOG00000004888	*Spred2*	1	1	1	1	1	0	0	1	0	16	8	28	1	28	M	M	M	M
160	4	57,453,921	57,453,921	1				+		ENSRNOG00000028092	*Cpa2*	1	1	1	1	0	0	0	1	0	11	-	28	-	28	M	-	M	-
161	6	93,898,598	93,898,598	1					+	-	-	1	1	1	1	1	0	1	0	0	28	25	18	3	28	P	P	P	P
162	1	47,230,506	47,230,506	1				+		ENSRNOG00000013190	*Rnaset2*	1	1	1	1	0	1	0	0	0	27	-	4	-	27	M	-	M	-

163	3	5,586,014	5,586,014	1				+		ENSRNOG00000032782	*Abo*	1	1	1	1	0	1	0	0	0	27	-	17	-	27	P	-	P	-
164	14	39,082,298	39,082,298	1				+		ENSRNOG00000002336	*Gabra4*	1	1	1	1	0	1	0	0	0	27	-	14	-	27	M	-	M	-
165	12	29,410,499	29,410,540	42	+				+	-	-	6	0	6	0	6	0	2	0	1	-	27	-	14	27	-	M	-	M
168	14	92,823,927	92,823,927	1				+		ENSRNOG00000004290	*Grb10*	1	1	1	1	0	0	0	1	0	15	-	27	-	27	P	-	P	-
169	1	112,570,666	112,570,943	278					+	-	-	3	0	3	0	3	0	3	0	1	-	26	-	24	26	-	P	-	P
170	13	25,998,049	25,998,242	194					+	-	-	9	0	9	0	9	0	4	0	1	-	26	-	14	26	-	P	-	P
172	17	39,979,276	39,979,276	1	+	+	+			ENSRNOG00000021741	*Hus1b*	1	1	1	1	0	0	0	1	0	9	-	26	-	26	M	-	M	-
173	10	44,709,509	44,709,509	1					+	-	-	1	0	1	0	0	0	1	0	0	-	26	-	-	26	-	P	-	-
174	13	78,759,650	78,759,650	1				+		ENSRNOG00000003698	*RGD1564745*	1	1	1	1	0	1	0	0	0	26	-	4	-	26	M	-	M	-
175	17	16,208,109	16,208,109	1	+				+	-	-	1	1	1	1	0	1	0	0	0	26	-	13	-	26	M	-	M	-
180	6	133,866,474	133,866,484	11					+	-	-	2	2	2	2	0	2	0	1	0	25	-	24	-	25	P	-	P	-
181	18	238,645	238,645	1	+				+	-	-	1	1	1	1	1	0	0	1	0	14	13	25	13	25	M	M	M	M
182	14	93,007,178	93,007,414	237					+	-	-	5	5	5	5	0	0	0	3	0	17	-	25	-	25	M	-	M	-
184	10	7,231,817	7,231,823	7			+			ENSRNOG00000002768	*Tmem114*	4	4	4	4	4	3	0	0	0	24	8	13	8	24	M	M	M	M
185	7	135,196,610	135,196,841	232					+	-	-	10	10	10	10	0	0	0	6	0	2	-	24	-	24	M	-	M	-
186	14	92,972,502	92,972,502	1					+	-	-	1	0	0	0	1	0	0	0	1	-	-	-	24	24	-	-	-	P
188	7	135,154,595	135,154,595	1					+	-	-	1	1	1	1	0	0	0	1	0	1	24	23	-	24	M	M	M	-
189	6	132,639,276	132,639,276	1				+		ENSRNOG00000029136	*Ccdc85c*	1	1	1	1	0	1	0	0	0	24	-	8	-	24	M	-	M	-
190	7	7,112,683	7,112,683	1					+	-	-	1	0	1	0	1	0	1	0	0	-	24	-	9	24	-	P	-	P
197	5	74,597,830	74,597,830	1			+			ENSRNOG00000026670	*Sec24c*	1	1	1	1	0	1	0	0	0	22	-	2	-	22	M	-	M	-
198	12	8,285,124	8,285,138	15					+	-	-	5	4	4	5	0	2	0	0	0	22	-	21	-	22	M	-	M	-
199	9	62,416,803	62,416,803	1				+		ENSRNOG00000012891	*Mdh1b*	1	0	1	0	1	0	1	0	0	-	22	-	5	22	-	P	-	P
202	1	113,144,110	113,144,110	1					+	-	-	1	1	1	1	0	0	0	1	0	8	-	21	-	21	P	-	P	-
203	13	65,931,612	65,931,612	1					+	-	-	1	0	1	0	1	0	1	0	0	-	21	-	4	21	-	P	-	P
204	10	57,639,965	57,639,965	1					+	-	-	1	1	1	1	0	1	0	0	0	21	-	5	-	21	M	-	M	-
205	1	203,797,947	203,797,947	1					+	-	-	1	1	1	1	0	1	0	0	0	21	-	18	-	21	M	-	M	-
207	1	113,665,634	113,665,634	1					+	-	-	1	1	1	1	0	1	0	0	0	20	-	6	-	20	P	-	P	-
211	8	42,848,209	42,848,209	1					+	-	-	1	1	1	1	0	1	0	0	0	20	-	2	-	20	M	-	M	-
212	5	146,988,842	146,988,842	1					+	-	-	1	0	0	0	1	0	0	0	1	-	16	-	20	20	-	P	-	P
214	1	116,594,477	116,594,816	340	+	+	+			ENSRNOG00000010146	*Ndn*	27	0	0	16	26	0	0	3	12	-	-	20	13	20	-	-	M	M
215	6	134,115,202	134,115,202	1					+	-	-	1	1	1	1	1	1	0	0	0	20	-	9	1	20	P	-	P	P
217	1	132,989,415	132,989,415	1					+	-	-	1	0	0	0	1	0	0	0	1	-	-	-	19	19	-	-	-	P
218	1	19,370,477	19,370,477	1					+	-	-	1	0	1	0	0	0	1	0	0	-	19	-	-	19	-	M	-	-
219	1	87,152,956	87,153,284	329	+		+			ENSRNOG00000022919	*Chst8*	25	25	25	25	25	5	0	7	0	18	13	19	14	19	M	M	M	M
220	1	203,156,415	203,156,415	1					+	-	-	1	0	0	0	1	0	0	0	1	-	4	-	18	18	-	M	-	M
222	15	11,700,289	11,700,289	1					+	-	-	1	1	1	1	0	1	0	0	0	18	-	2	-	18	P	-	P	-
223	8	112,645,731	112,645,731	1	+			+		ENSRNOG00000015835	*Cacna2d2*	1	1	1	1	0	0	0	1	0	18	-	18	-	18	P	-	P	-
227	16	18,572,189	18,572,257	69	+				+	-	-	5	5	5	5	0	0	0	2	0	9	-	18	-	18	M	-	M	-
230	12	36,587,453	36,587,453	1					+	-	-	1	0	0	0	1	0	0	0	1	-	-	-	17	17	-	-	-	P
231	3	4,877,929	4,877,929	1					+	-	-	1	0	1	0	1	0	1	0	0	-	17	-	9	17	-	P	-	P
234	19	39,232,330	39,232,330	1					+	-	-	1	1	1	1	0	0	0	1	0	8	-	17	-	17	M	-	M	-
235	4	151,730,550	151,730,550	1					+	-	-	1	0	1	0	1	0	1	0	0	-	16	-	8	16	-	P	-	P
236	10	81,289,852	81,289,852	1					+	-	-	1	1	1	1	0	1	0	0	0	16	-	5	-	16	P	-	P	-
238	17	11,881,412	11,881,412	1					+	-	-	1	1	1	1	0	1	0	0	0	16	-	1	-	16	M	-	M	-
243	1	116,635,990	116,635,990	1					+	-	-	1	0	1	0	0	0	1	0	0	-	15	-	-	15	-	P	-	-
245	1	5,220,021	5,220,021	1					+	-	-	1	1	1	0	0	1	0	0	0	15	-	-	-	15	M	-	-	-
246	20	9,067,790	9,067,790	1				+		ENSRNOG00000000542	*Dnah8*	1	1	1	1	1	0	0	0	1	0	-	0	15	15	-	-	-	P
249	17	15,667,896	15,667,896	1			+			ENSRNOG00000026235	*Hk3*	1	1	1	1	0	0	0	1	0	4	-	14	-	14	P	-	P	-
255	2	242,620,332	242,620,332	1					+	-	-	1	0	1	0	1	0	1	0	0	-	11	-	0	11	-	P	-	-
259	13	69,122,084	69,122,084	1	+	+	+			ENSRNOG00000031569	*Zfp648*	1	0	1	0	0	0	1	0	0	-	7	-	-	7	-	P	-	-
260	7	118,951,027	118,951,044	18	+	+				ENSRNOG00000018330	*Fam83f*	3	3	3	0	0	2	0	0	0	5	1	-	-	5	P	P	-	-

For each region the table provides details on the location and size, whether it overlaps with a CpG island (CGI), with the transcription start site (TSS = 2kb window centred at the transcription start site), exons, introns or is intergenic (1 = overlap; 0 = no overlap). If the region overlaps with a TSS, intron or exon the gene ID and gene name of the respective gene is given. All TSS, intron and exon coordinates are based on Ensembl (version 62) annotations. Parent-of-origin specific methylation differences were determined on BN derived chromosomes and on SHR derived chromosomes separately. Increased methylation of the maternal allele is denoted as M, increased methylation of the paternal allele is denoted as P.

a) Statistically significant differences after multiple testing correction (FDR < 5%).

b) Regions are as defined in the Methods.

c) CGI = CpG island as defined in the UCSC genome browser (cpgIslandGgfAndyMasked).

d) TSS = 2kb window centred at the transcription start site as defined in the Ensembl genome browser version 62.

e) Intergenic = the region does not overlap with a TSS, exon or intron.

f) Allele showing increased methylation: M = maternally derived allele; P = paternally derived allele.
