# Supplementary material for: Genetic Analysis of the Cardiac Methylome at Single Nucleotide Resolution in a Model of Human Cardiovascular Disease
Source: PLoS Genet. 2014 Dec 4;10(12):e1004813. doi: 10.1371/journal.pgen.1004813 (PMC4256262; doi:10.1371/journal.pgen.1004813)
Supplement: Table S10 — Summary of validation of parent-of-origin specific differences in methylation by targeted bisulfite-seq. (PDF) [file pgen.1004813.s023.pdf]

Table S10 Summary of validation of parent-of-origin specific differences in methylation by targeted bisulfite-seq

					BN								SHR							
Region ID[a]	Locus	Amplicon Coordinates	CpG Position	Strand	Maternal Coverage [x-fold]	Maternal Methylation Level [%]	Paternal Coverage [x-fold]	paternal Methylation Level [%]	Delta Methylation Level (Maternal-Paternal) [%]	P_{fisher} Adjusted[b]	sig.[c]	Allele Showing Increased Methylation[d]	Maternal Coverage [x-fold]	Maternal Methylation Level [%]	Paternal Coverage [x-fold]	Paternal Methylation Level [%]	Delta Methylation Level (Maternal-Paternal) [%]	P_{fisher} Adjusted[b]	sig.[c]	Allele Showing Increased Methylation[d]
44	F1LUE4_RAT-Sgk1	chr1:23,480,246-23,480,522	23,480,271	+	1035	80	1033	43	37	3.00E-068	*	M	1037	80	1037	73	7	3.73E-004	*	M
44	F1LUE4_RAT-Sgk1	chr1:23,480,246-23,480,522	23,480,286	+	1039	58	1036	16	42	1.77E-091	*	M	1036	52	1039	65	-12	1.09E-008	*	P
44	F1LUE4_RAT-Sgk1	chr1:23,480,246-23,480,522	23,480,303	+	1039	79	1034	49	30	2.03E-045	*	M	1036	81	1038	77	4	3.24E-002	*	M
44	F1LUE4_RAT-Sgk1	chr1:23,480,246-23,480,522	23,480,308	+	1039	75	1036	62	13	9.81E-011	*	M	1036	91	1037	72	19	8.33E-030	*	M
44	F1LUE4_RAT-Sgk1	chr1:23,480,246-23,480,522	23,480,318	+	1036	75	1036	46	29	6.01E-042	*	M	1035	77	1040	59	18	1.23E-018	*	M
44	F1LUE4_RAT-Sgk1	chr1:23,480,246-23,480,522	23,480,342	+	1038	81	1031	60	21	6.49E-026	*	M	1037	92	1039	75	17	3.58E-026	*	M
44	F1LUE4_RAT-Sgk1	chr1:23,480,246-23,480,522	23,480,347	+	1034	91	1033	55	36	5.19E-081	*	M	1032	95	1033	84	11	3.41E-017	*	M
44	F1LUE4_RAT-Sgk1	chr1:23,480,246-23,480,522	23,480,349	+	1032	88	1033	57	31	2.24E-058	*	M	1032	96	1031	81	15	1.73E-027	*	M
44	F1LUE4_RAT-Sgk1	chr1:23,480,246-23,480,522	23,480,352	+	1032	93	1030	68	25	1.11E-049	*	M	1033	88	1032	84	4	1.23E-002	*	M
44	F1LUE4_RAT-Sgk1	chr1:23,480,246-23,480,522	23,480,359	+	984	92	988	70	22	7.79E-036	*	M	95	86	82	79	7	2.45E-001		M
44	F1LUE4_RAT-Sgk1	chr1:23,480,246-23,480,522	23,480,361	+	986	90	988	62	28	4.22E-049	*	M	97	76	82	79	-3	7.31E-001		P
44	F1LUE4_RAT-Sgk1	chr1:23,480,246-23,480,522	23,480,363	+	1032	87	1026	60	27	4.16E-044	*	M	1030	93	1031	82	10	3.03E-012	*	M
44	F1LUE4_RAT-Sgk2	chr1:23,480,246-23,480,522	23,480,365	+	1035	89	1024	67	21	4.97E-032	*	M	1033	97	1036	89	8	2.69E-013	*	M
44	F1LUE4_RAT-Sgk3	chr1:23,480,246-23,480,522	23,480,369	+	1036	81	1034	53	28	1.12E-042	*	M	1039	72	1040	73	-1	5.68E-001		P
44	F1LUE4_RAT-Sgk4	chr1:23,480,246-23,480,522	23,480,438	+	654	52	656	18	34	2.82E-038	*	M	412	66	501	52	13	8.11E-005	*	M
44	F1LUE4_RAT-Sgk5	chr1:23,480,246-23,480,522	23,480,444	+	654	63	662	27	36	8.81E-041	*	M	412	70	501	58	12	2.73E-004	*	M
44	F1LUE4_RAT-Sgk6	chr1:23,480,246-23,480,522	23,480,473	+	655	76	662	51	25	7.94E-021	*	M	411	85	499	74	11	3.44E-005	*	M
44	F1LUE4_RAT-Sgk7	chr1:23,480,246-23,480,522	23,480,494	+	633	80	641	60	20	5.93E-015	*	M	383	93	468	78	16	4.78E-011	*	M
214		chr1:116,594,592-116,594,886	116,594,623	+	1063	47	1061	2	45	6.01E-151	*	M	1053	9	1055	1	8	1.77E-017	*	M
214		chr1:116,594,592-116,594,886	116,594,623	-	1064	48	1055	1	47	1.90E-163	*	M	1065	34	1053	0	33	1.38E-111	*	M
214		chr1:116,594,592-116,594,886	116,594,632	+	1064	28	1060	1	28	1.36E-088	*	M	1054	5	1053	0	5	4.50E-014	*	M
214		chr1:116,594,592-116,594,886	116,594,632	-	1065	39	1059	5	34	9.10E-088	*	M	1066	33	1057	1	33	1.23E-107	*	M
214		chr1:116,594,592-116,594,886	116,594,637	+	1066	9	1059	1	8	3.46E-020	*	M	1054	16	1055	4	12	2.94E-020	*	M
214		chr1:116,594,592-116,594,886	116,594,637	-	1068	19	1061	1	18	1.52E-052	*	M	1065	31	1058	0	31	6.94E-105	*	M
214		chr1:116,594,592-116,594,886	116,594,664	-	1063	13	1060	1	12	3.87E-032	*	M	1067	27	1057	0	27	8.31E-089	*	M
214		chr1:116,594,592-116,594,886	116,594,674	+	1064	37	1062	1	37	1.03E-127	*	M	1054	25	1056	1	24	8.65E-070	*	M
214		chr1:116,594,592-116,594,886	116,594,674	-	1063	23	1062	1	22	1.51E-067	*	M	1064	16	1056	1	15	1.19E-042	*	M
214		chr1:116,594,592-116,594,886	116,594,696	-	725	10	587	3	6	8.56E-006	*	M	610	2	553	4	-2	1.00E-001		P
214		chr1:116,594,592-116,594,886	116,594,723	+	1066	29	1062	3	27	1.58E-070	*	M	1055	22	1057	2	20	3.61E-055	*	M
214		chr1:116,594,592-116,594,886	116,594,723	-	736	30	586	1	29	3.55E-054	*	M	610	13	553	1	12	1.90E-015	*	M
214		chr1:116,594,592-116,594,886	116,594,744	+	997	11	995	5	7	6.33E-008	*	M	971	31	996	1	30	8.38E-088	*	M
214		chr1:116,594,592-116,594,886	116,594,744	-	993	14	919	1	13	1.44E-031	*	M	969	14	967	2	12	9.64E-024	*	M
214		chr1:116,594,592-116,594,886	116,594,755	+	1017	37	1022	3	35	5.25E-096	*	M	1014	47	1020	3	44	5.55E-135	*	M
214		chr1:116,594,592-116,594,886	116,594,755	-	999	49	923	3	46	4.27E-132	*	M	972	34	973	8	26	2.74E-046	*	M
214		chr1:116,594,592-116,594,886	116,594,774	+	1061	16	1059	3	13	3.67E-025	*	M	1059	21	1060	1	19	9.72E-055	*	M
214		chr1:116,594,592-116,594,886	116,594,774	-	1000	18	921	3	15	1.13E-029	*	M	975	26	970	2	24	6.45E-061	*	M
214		chr1:116,594,592-116,594,886	116,594,786	+	1065	32	1062	4	28	1.50E-070	*	M	1061	31	1060	3	28	1.76E-069	*	M
214		chr1:116,594,592-116,594,886	116,594,786	-	1003	31	923	4	26	2.16E-056	*	M	974	35	970	2	33	8.62E-089	*	M
214		chr1:116,594,592-116,594,886	116,594,788	+	1064	30	1063	3	26	8.88E-068	*	M	1060	29	1059	2	27	3.20E-074	*	M
214		chr1:116,594,592-116,594,886	116,594,788	-	1003	36	923	2	34	7.97E-089	*	M	975	15	973	3	12	6.24E-021	*	M
214		chr1:116,594,592-116,594,886	116,594,806	+	1066	17	1063	3	14	3.41E-027	*	M	1061	34	1059	2	32	1.09E-095	*	M
214		chr1:116,594,592-116,594,886	116,594,806	-	1003	34	922	2	32	1.23E-082	*	M	973	18	973	4	14	1.63E-025	*	M
214		chr1:116,594,592-116,594,886	116,594,810	+	1059	22	1063	1	21	1.29E-063	*	M	1061	7	1059	2	5	1.32E-008	*	M
214		chr1:116,594,592-116,594,886	116,594,810	-	1001	12	921	3	9	7.01E-015	*	M	972	18	972	2	16	4.92E-037	*	M
214		chr1:116,594,592-116,594,886	116,594,815	+	1065	34	1063	2	32	2.62E-099	*	M	1062	22	1059	3	19	1.11E-042	*	M
214		chr1:116,594,592-116,594,886	116,594,815	-	985	26	905	2	23	1.18E-053	*	M	956	10	954	6	5	1.64E-004	*	M
214		chr1:116,594,592-116,594,886	116,594,845	+	1066	8	1063	1	7	2.82E-015	*	M	1061	10	1060	1	9	2.80E-022	*	M
214		chr1:116,594,592-116,594,886	116,594,862	+	1062	4	1054	2	2	7.80E-004	*	M	1059	8	1057	0	8	3.01E-021	*	M
111		chr1:202,823,579-202,823,854	202,823,599	+	1065	74	1069	92	-19	1.06E-030	*	M	1064	58	1067	82	-25	3.61E-036	*	P
111		chr1:202,823,579-202,823,854	202,823,614	+	1067	79	1074	97	-18	6.27E-043	*	M	1068	76	1069	94	-18	2.33E-031	*	P
111		chr1:202,823,579-202,823,854	202,823,630	+	1065	12	1076	89	-77	5.01E-030	*	M	1071	25	1069	85	-60	2.85E-182	*	P
111		chr1:202,823,579-202,823,854	202,823,635	+	1065	11	1076	79	-67	7.51E-234	*	M	1071	30	1070	85	-55	3.07E-153	*	P
111		chr1:202,823,579-202,823,854	202,823,697	+	405	75	361	89	-14	5.41E-007	*	M	317	82	388	96	-14	7.10E-010	*	P
111		chr1:202,823,579-202,823,854	202,823,715	+	998	66	991	97	-31	1.91E-077	*	M	983	52	976	63	-11	7.59E-007	*	P
97		chr4:57,445,980-57,446,560	57,446,138	+	335	75	544	12	63	5.54E-082	*	M	381	74	406	16	58	1.04E-063	*	M
97		chr4:57,445,980-57,446,560	57,446,147	+	335	77	538	18	59	2.19E-068	*	M	381	65	405	25	40	2.67E-030	*	M
97		chr4:57,445,980-57,446,560	57,446,230	+	910	59	812	4	55	3.26E-150	*	M	594	44	741	4	39	1.40E-070	*	M
97		chr4:57,445,980-57,446,560	57,446,235	+	909	63	814	11	52	1.97E-117	*	M	596	57	742	13	44	4.96E-066	*	M
97		chr4:57,445,980-57,446,560	57,446,241	+	909	28	811	1	27	3.36E-064	*	M	594	31	739	8	23	2.04E-027	*	M
97		chr4:57,445,980-57,446,560	57,446,376	+	495	99	755	92	7	1.32E-008	*	M	736	91	678	94	-3	2.05E-002	*	P
97		chr4:57,445,980-57,446,560	57,446,376	-	1042	99	1025	93	5	8.70E-010	*	M	1043	94	1042	97	-3	4.93E-004	*	P
97		chr4:57,445,980-57,446,560	57,446,389	+	496	86	756	70	16	6.78E-011	*	M	737	92	678	73	19	4.29E-021	*	M
97		chr4:57,445,980-57,446,560	57,446,389	-	1045	97	1034	68	28	1.77E-072	*	M	1043	97	1043	74	23	1.04E-056	*	M

97	chr4:57,445,980-57,446,560	57,446,414	+	582	93	852	99	-6	5.81E-009	*	M	794	100	723	93	7	5.32E-014	*	M
97	chr4:57,445,980-57,446,560	57,446,414	-	574	100	583	100	0	1.00E+000		M	641	100	589	95	5	5.22E-009	*	M
97	chr4:57,445,980-57,446,560	57,446,417	+	633	88	880	84	4	2.61E-002	*	M	829	93	748	93	0	7.65E-001		M
97	chr4:57,445,980-57,446,560	57,446,417	-	597	91	607	93	-1	3.97E-001		M	666	96	604	79	16	2.66E-020	*	M
97	chr4:57,445,980-57,446,560	57,446,508	+	994	94	990	89	5	2.57E-004	*	M	1002	95	1003	87	8	3.02E-009	*	M
97	chr4:57,445,980-57,446,560	57,446,508	-	975	99	968	96	4	9.72E-009	*	M	989	96	983	70	26	2.66E-058	*	M
97	chr4:57,445,980-57,446,560	57,446,523	+	987	88	991	93	-4	1.31E-003	*	M	1007	90	1011	88	2	2.07E-001		M
97	chr4:57,445,980-57,446,560	57,446,523	-	974	99	964	95	5	4.38E-010	*	M	986	100	983	81	19	9.92E-060	*	M
147	chr5:141,638,136-141,638,442	141,638,171	+	1044	78	1049	53	25	4.96E-033	*	M	1046	78	1056	71	8	7.00E-005	*	M
147	chr5:141,638,136-141,638,442	141,638,199	+	1043	90	1052	44	45	2.54E-114	*	M	1047	85	1055	48	38	1.72E-076	*	M
147	chr5:141,638,136-141,638,442	141,638,215	+	1044	68	1050	24	45	9.60E-096	*	M	1049	55	1055	56	-1	7.32E-001		P
147	chr5:141,638,136-141,638,442	141,638,252	+	1108	78	1117	44	34	1.19E-061	*	M	1115	61	1112	31	30	3.41E-045	*	M
147	chr5:141,638,136-141,638,442	141,638,271	+	903	81	906	47	33	6.75E-050	*	M	872	85	957	37	48	1.41E-102	*	M
147	chr5:141,638,136-141,638,442	141,638,284	+	957	78	933	62	17	1.36E-015	*	M	924	80	975	62	18	3.45E-017	*	M
147	chr5:141,638,136-141,638,442	141,638,300	+	1007	89	1007	87	3	7.49E-002		M	1009	93	1007	76	17	2.75E-027	*	M
147	chr5:141,638,136-141,638,442	141,638,363	+	1042	71	1046	50	21	7.04E-023	*	M	1040	72	1037	69	3	2.22E-001		M
147	chr5:141,638,136-141,638,442	141,638,386	+	1079	55	1081	49	6	3.57E-003	*	M	1069	59	1072	46	14	2.50E-010	*	M
79	chr14:18,639,302-18,639,840	18,639,378	+	1084	29	1089	4	26	1.49E-064	*	M	1067	18	1092	24	-6	1.12E-003	*	P
79	chr14:18,639,302-18,639,840	18,639,378	-	1047	46	1050	2	43	4.91E-139	*	M	1044	36	1050	8	28	2.26E-058	*	M
79	chr14:18,639,302-18,639,840	18,639,417	+	1094	59	1101	28	31	9.12E-048	*	M	1076	57	1099	38	19	3.60E-018	*	M
79	chr14:18,639,302-18,639,840	18,639,417	-	1046	54	1049	11	43	1.50E-103	*	M	1045	60	1049	47	13	1.91E-009	*	M
79	chr14:18,639,302-18,639,840	18,639,419	+	1097	37	1101	23	14	1.57E-012	*	M	1076	35	1097	33	2	2.88E-001		M
79	chr14:18,639,302-18,639,840	18,639,419	-	1047	54	1050	15	39	6.71E-081	*	M	1046	26	1050	46	-20	7.36E-022	*	P
79	chr14:18,639,302-18,639,840	18,639,467	+	988	49	981	40	9	6.92E-005	*	M	917	65	917	30	35	1.63E-051	*	M
79	chr14:18,639,302-18,639,840	18,639,467	-	1003	66	1001	6	60	2.64E-192	*	M	1004	48	1011	26	21	3.99E-023	*	M
79	chr14:18,639,302-18,639,840	18,639,471	+	991	56	980	41	16	4.26E-012	*	M	918	71	919	53	19	1.55E-016	*	M
79	chr14:18,639,302-18,639,840	18,639,471	-	570	90	601	20	70	5.71E-140	*	M	516	77	709	54	23	1.58E-016	*	M
79	chr14:18,639,302-18,639,840	18,639,483	+	1009	57	1004	57	0	9.00E-001		M	931	80	936	55	24	2.22E-029	*	M
79	chr14:18,639,302-18,639,840	18,639,483	-	641	94	643	50	44	3.21E-074	*	M	583	96	801	58	39	4.65E-068	*	M
79	chr14:18,639,302-18,639,840	18,639,510	+	1036	71	1046	45	26	3.97E-033	*	M	1031	82	1025	53	30	4.29E-047	*	M
79	chr14:18,639,302-18,639,840	18,639,510	-	646	93	645	32	61	8.34E-126	*	M	587	72	807	35	37	1.78E-042	*	M
79	chr14:18,639,302-18,639,840	18,639,525	+	1040	65	1046	47	18	8.28E-017	*	M	1034	82	1028	60	23	2.43E-030	*	M
79	chr14:18,639,302-18,639,840	18,639,525	-	645	91	647	21	70	3.49E-155	*	M	586	66	806	37	29	2.63E-027	*	M
79	chr14:18,639,302-18,639,840	18,639,565	+	1036	59	1046	21	38	5.91E-072	*	M	1030	58	1027	39	19	4.59E-018	*	M
79	chr14:18,639,302-18,639,840	18,639,565	-	647	64	639	4	60	7.44E-128	*	M	586	85	807	38	48	2.20E-075	*	M
79	chr14:18,639,302-18,639,840	18,639,644	+	964	76	939	42	34	6.54E-053	*	M	903	78	875	59	19	7.38E-018	*	M
79	chr14:18,639,302-18,639,840	18,639,644	-	1070	66	1070	29	38	1.21E-068	*	M	1063	81	1064	46	35	2.22E-064	*	M
79	chr14:18,639,302-18,639,840	18,639,652	+	965	68	940	43	25	5.05E-029	*	M	903	77	874	61	16	9.50E-014	*	M
79	chr14:18,639,302-18,639,840	18,639,652	-	1020	71	1031	39	32	4.58E-047	*	M	982	81	998	55	26	1.28E-036	*	M
79	chr14:18,639,302-18,639,840	18,639,674	+	966	62	941	30	32	7.17E-045	*	M	904	70	876	28	42	4.45E-070	*	M
79	chr14:18,639,302-18,639,840	18,639,674	-	1029	51	1036	17	34	2.43E-062	*	M	1007	70	1008	29	41	7.66E-077	*	M
79	chr14:18,639,302-18,639,840	18,639,679	+	982	80	952	50	30	1.19E-044	*	M	914	89	880	76	13	2.83E-012	*	M
79	chr14:18,639,302-18,639,840	18,639,679	-	1033	73	1037	54	19	1.12E-019	*	M	1009	86	1009	62	24	7.69E-034	*	M
79	chr14:18,639,302-18,639,840	18,639,683	+	1002	83	969	44	39	1.02E-075	*	M	925	75	891	73	2	5.02E-001		M
79	chr14:18,639,302-18,639,840	18,639,683	-	1033	66	1035	34	32	1.11E-047	*	M	1008	82	1009	50	32	1.63E-051	*	M
79	chr14:18,639,302-18,639,840	18,639,696	+	1001	76	886	46	31	2.94E-043	*	M	930	82	835	63	19	2.66E-019	*	M
79	chr14:18,639,302-18,639,840	18,639,696	-	1033	76	1035	36	40	9.11E-076	*	M	1010	82	1007	43	39	3.37E-075	*	M
79	chr14:18,639,302-18,639,840	18,639,713	+	1022	86	895	49	37	8.78E-071	*	M	943	82	844	71	11	4.30E-008	*	M
79	chr14:18,639,302-18,639,840	18,639,713	-	1037	74	1037	47	27	7.64E-036	*	M	1014	82	1012	52	31	3.58E-049	*	M
79	chr14:18,639,302-18,639,840	18,639,717	+	1032	79	1011	45	34	5.26E-057	*	M	1000	89	995	52	37	9.61E-077	*	M
79	chr14:18,639,302-18,639,840	18,639,717	-	1028	71	1037	34	37	6.20E-064	*	M	1002	79	1001	52	28	4.40E-039	*	M
79	chr14:18,639,302-18,639,840	18,639,728	+	1051	74	1024	38	36	2.34E-061	*	M	1012	75	1005	33	42	4.09E-083	*	M
79	chr14:18,639,302-18,639,840	18,639,728	-	988	61	983	25	36	2.16E-058	*	M	959	74	962	44	31	1.13E-004	*	M
79	chr14:18,639,302-18,639,840	18,639,739	+	1056	71	1026	33	37	2.22E-066	*	M	1017	77	1008	53	24	3.48E-002	*	M
79	chr14:18,639,302-18,639,840	18,639,739	-	990	69	983	31	38	1.06E-063	*	M	961	76	962	43	33	1.03E-050	*	M
79	chr14:18,639,302-18,639,840	18,639,755	+	1051	62	1022	30	32	6.79E-050	*	M	1014	66	1010	22	44	1.02E-009	*	M
79	chr14:18,639,302-18,639,840	18,639,755	-	992	45	983	21	24	5.05E-029	*	M	967	58	962	19	38	8.45E-068	*	M
79	chr14:18,639,302-18,639,840	18,639,805	+	1009	83	985	57	26	3.58E-038	*	M	1001	91	952	72	19	1.63E-002	*	M
79	chr14:18,639,302-18,639,840	18,639,805	-	379	83	401	46	37	1.70E-027	*	M	348	85	346	53	33	6.12E-021	*	M

Parent-of-origin specific methylation differences were determined on BN-derived chromosomes and on SHR-derived chromosomes separately. Increased methylation of the maternal allele is denoted as M, increased methylation of the paternal allele is denoted as P.

a) Region IDs refer to the ID in the #-column of Table S7.

b) P values adjusted for multiple testing using the FDR method by Benjamini and Hochberg (Benjamini and Hochberg 1995).

c) Methylation differences statistically significant after multiple-testing correction (FDR < 5%) are marked with '*'.

d) Allele showing increased methylation: M = maternally derived allele; P = paternally derived allele.
