# Supplementary material for: Genetic Analysis of the Cardiac Methylome at Single Nucleotide Resolution in a Model of Human Cardiovascular Disease
Source: PLoS Genet. 2014 Dec 4;10(12):e1004813. doi: 10.1371/journal.pgen.1004813 (PMC4256262; doi:10.1371/journal.pgen.1004813)
Supplement: Table S11 — Phenotypes tested for correlation with methylation measurements across the RI strain panel. (PDF) [file pgen.1004813.s024.pdf]

Table S11 Phenotypes testes for correlation with methylation measurements across the RI strain panel

#	Phenotype Name
1	ADP_induced_platelet_aggregation
2	adrenal_Chga_chromogranin_A_fragments_ml
3	adrenal_Chgb_chromogranin_B_fragments_ml
4	adrenal_corticosterone_IU_mg_protein
5	adrenal_dopamine_beta_hydroxylase_pmolhmg_protein
6	adrenal_dopamine_ng_mg_protein
7	Adrenal_Epinephrine
8	adrenal_epinephrine_ng_mg_protein
9	adrenaline_stimulated_lipolysis_glycerol_levels_mmol_g_tissue_2h
10	adrenaline_stimulated_lipolysis_NEFA_levels_mmol_g_tissue_2h
11	adrenal_norepinephrine_ng_mg_protein
12	Adrenal_Norpinephrine
13	adrenal_PNMT_phenyleta_nolamine_mythyl_transferase_pmolhmg_protein
14	adrenal_Scg2_secretogranin_II_fragments_ml
15	area_under_the_curve_during_intraperitoneal_glucose_tolerance_test_in_male_rats_fed_a_diet_with_60per_fructose_from_8_to_10_weeks
16	area_under_the_curve_during_oral_glucose_tolerance_test_in_male_rats_fed_a_diet_with_60per_fructose_from_8_to_10_weeks_for_blood_glucose_level_mmolL2h
17	AUC_during_OGTT_for_insulin
18	AUC_during_OGTT_glycemia
19	basal_glucose_uptake_corrected_to_cell
20	basal_glycogenesis_in_diaphragm_nmol_glucose_g_2h
21	basal_lipogenesis_in_epididymal_fat_nmol_glucose_g2h
22	basal_lipogenesis_in_epididymal_fat_nmol_glucose_mg_protein2h
23	basal_lipolysis_glycerol_levels_mmol_g_tissue_2h
24	basal_lipolysis_NEFA_levels_mmol_g_tissue_2h
25	basal_uptake_of_glucose_by_isolated_adipocytes_corrected_to_cell_volume
26	blood_glucose_mmol_L_0_in_during_OGTT
27	blood_glucose_mmol_L_120_min_during_OGTT
28	blood_glucose_mmol_L_30_min_during_OGTT
29	blood_glucose_mmol_L_60_min_during_OGTT
30	body_weight_of_mothers_g
31	body_weights_g_males_10_wk_old
32	CgA
33	CgB
34	cholesterol_content_in_liver
35	Compensatory_renal_growth_in_males_8_weeks_versus_5_weeks_scaled_to_body_weight
36	Conditioned_taste_aversion
37	Conditioned_taste_aversion_arcsin_transform
38	Corticosterone
39	DBH
40	Decapitated
41	delta_captopril_effects_mm_Hg
42	delta_L_NAME_effects_mm_Hg
43	delta_pentolinium_effects_mm_Hg
44	diastolic_blood_pressure_determined_by_direct_puncture_of_the_carotida_artery_under_light_ether_anaesthesia_in_12_week_old_males_fed_a_normal_lab_chow_at_generations_F16_F17
45	diastolic_blood_pressure_determined_by_direct_puncture_of_the_carotida_artery_under_light_ether_anaesthesia_in_12_week_old_males_fed_a_normal_lab_chow_at_generations_F16_F18
46	diastolic_blood_pressure_determined_by_direct_puncture_of_the_carotida_artery_under_light_ether_anaesthesia_in_12_week_old_males_fed_a_normal_lab_chow_at_generations_F16_F19
47	diastolic_blood_pressure_determined_by_direct_puncture_of_the_carotida_artery_under_light_ether_anaesthesia_in_12_week_old_males_fed_a_normal_lab_chow_at_generations_F16_F20
48	diastolic_blood_pressure_determined_by_direct_puncture_of_the_carotida_artery_under_light_ether_anaesthesia_in_12_week_old_males_fed_a_normal_lab_chow_at_generations_F16_F21
49	diastolic_blood_pressure_determined_by_direct_puncture_of_the_carotida_artery_under_light_ether_anaesthesia_in_12_week_old_males_fed_a_normal_lab_chow_at_generations_F16_F22
50	diastolic_blood_pressure_determined_by_direct_puncture_of_the_carotida_artery_under_light_ether_anaesthesia_in_12_week_old_males_fed_a_normal_lab_chow_at_generations_F16_F23
51	difference_between_ins_0_vol_and_ins_50_vol
52	difference_between_maximal_and_basal_glucose_uptake_corrected_to_cell
53	dry_weight_of_copper_concentration_in_livers
54	Epididymus_weight
55	Epididymus_weight_corrected_to_body_weight
56	epididymus_weight_g_10_wks_old_rats
57	epididymus_weight_g_10_wks_old_rats_corrected_to_100g_body_weight
58	fat_cell_volume
59	fetal_weights_g_in_left_horn_of_the_uterus
60	fetal_weights_g_in_right_horn_of_the_uterus
61	glucose_concentrations_10_weeks_old_fed_a_diet_with_60per_fructose_from_8_weeks_to_10_weeks_male
62	glucose_concentrations_8_weeks_old_fed_a_normal_lab_chow_male
63	glucose_concentrations_before_intraperitoneal_glucose_tolerance_test_in_male_rats_10_weeks_old_fed_a_diet_with_60per_fructose
64	glucose_concentrations_during_intraperitoneal_glucose_tolerance-test_120_min_after_glucose_load_in_male_rats_10_weeks_old_fed_a_diet_with_60per_fructose
65	glucose_concentrations_during_intraperitoneal_glucose_tolerance_test_30_min_after_glucose_load_in_male_rats_10_weeks_old_fed_a_diet_with_60per_fructose
66	glucose_concentrations_during_intraperitoneal_glucose_tolerance_test_60_min_after_glucose_load_in_male_rats_10_weeks_old_fed_a_diet_with_60per_fructose
67	hematocrit_at_the_age_of_30_days_in_males_fed_a_standard_chow
68	hematocrit_at_the_age_of_90_days_in_males_fed_a_standard_chow
69	insulin_concentrations_10_weeks_old_fed_a_diet_with_60per_fructose_from_8_weeks_to_10_weeks_15_days_male
70	insulin_concentrations_8_weeks_old_fed_a_normal_lab_chow_0_days_male
71	insulin_glucose_ratio_10_weeks_old_fed_a_diet_with_60per_fructose_from_8_weeks_to_10_weeks_male
72	insulin_glucose_ratio_8_weeks_old_fed_a_normal_lab_chow_male
73	Insulin_nmoll
74	insulin_stimulated_glycogenesis_in_diaphragm_nmol_glucose_g_2h
75	insulin_stimulated_lipogenesis_in_epididymal_fat_nmol_glucose_g2h
76	insulin_stimulated_lipogenesis_in_epididymal_fat_nmol_glucose_mg_protein2h
77	insulin_stimulated_uptake_of_glucose_by_isolated_adipocytes_corrected_to_cell_volume
78	Interleukin6_IL12
79	Interleukin6_IL13
80	Interleukin6_IL6
81	Interleukin6_IL8
82	Interleukin6_IL9
83	isoproterenol_induced_lipolysis_of_isolated_adipocytes_corrected_to_cell
84	isoproterenol_induced_lipolysis_of_isolated_adipocytes_corrected_to_cell_volume
85	isoproterenol_induced_lipolysis_of_isolated_adipocytes_corrected_to_volume_multiplied
86	isoproterenol_induced_lipolysis_of_isolated_adipocytes_uncorrected
87	kidney_cortex_catalase_M_H2O2_min_mg_protein
88	kidney_cortex_conjugated_dienes_nM_mg_protein
89	kidney_cortex_glutathione_M_mg_protein
90	kidney_cortex_glutathione_reductase_M_NADPH_min_mg_protein_
91	kidney_cortex_GSH_Px_seleno_dependent_superoxide_peroxidase_M_GSH_min_mg_protein
92	kidney_cortex_SOD_superoxide_dismutase_U_mg_protein_10_wks_old_males
93	kidney_cortex_TBARS_nM_mg_protein
94	Kidney_weight
95	Kidney_weight_corrected_to_body_weight
96	Leptin
97	Lipogenesis_in_epididymal_fat_0imp_g
98	Lipogenesis_in_epididymal_fat_0imp_mgprot
99	Lipogenesis_in_epididymal_fat_0nmol_glg2h
100	Lipogenesis_in_epididymal_fat_0nmol_glmgp2h
101	Lipogenesis_in_epididymal_fat_250imp_g
102	Lipogenesis_in_epididymal_fat_250imp_mgprot
103	Lipogenesis_in_epididymal_fat_250_nmol_glg2h
104	Lipogenesis_in_epididymal_fat_250nmol_glmgp_2h
105	Lipolysis_Glycerol_0_level
106	Lipolysis_Glycerol_250_level
107	Lipolysis_NEFA_0_level

108 liver_conjugated_dienes_nM_mg_protein
109 liver_copper_concentration_after_rats_were_fed_a_diet_with_high_fat_high_cholesterol_diet_for_4_weeks_whole_liver_pool
110 liver_glutathione_M_mg_protein
111 liver_glutathione_reductase_M_NADPH_min_mg_protein
112 liver_GSH_Px_seleno_dependent_superoxide_peroxidase_M_GSH_min_mg_protein
113 liver_SOD_superoxide_dismutase_U_mg_protein_10_wks_old_males
114 liver_TBARS_nM_mg_protein
115 liver_triglycerides_mmol_g
116 Liver_weight
117 Liver_weight_corrected_to_body_weight
118 liver_weight_g_10_wks_old_rats
119 liver_weight_g_10_wks_old_rats_corrected_to_100g_body_weight
120 liver_weight_in_males_fed_a_high_fat_high_cholesterol_diet_from_the_age_of_7_weeks_till_the_age_of_11_weeks
121 liver_weights_in_males_fed_a_high_fat_high_cholesterol_diet_from_the_age_of_7_weeks_till_the_age_of_11_weeks
122 log_maximal_basal_glucose_uptake
123 log_of_insulin_glucose_ratio_day_15_2_weeks_of_fructose_feeding
124 MAP_mm_Hg_direct_measurement_in_2007
125 MAP_mm_Hg_direct_measurement_in_2008
126 MAP_mm_Hg_direct_measurement_in_2009
127 MAP_mm_Hg_direct_measurement_in_2010
128 maximal_basal_glucose_uptake
129 maximal_glucose_uptake_corrected_to_cell
130 mean_arterial_pressure_determined_by_direct_puncture_of_the_carotid_artery_under_light_ether_anaesthesia_in_12_week_old_males_fed_a_normal_lab_chow_at_generations_F16_F17
131 mean_arterial_pressure_determined_by_direct_puncture_of_the_carotid_artery_under_light_ether_anaesthesia_in_12_week_old_males_fed_a_normal_lab_chow_at_generations_F16_F18
132 Monocyte_chemotactic_protein_1
133 Monocyte_chemotactic_protein_2
134 Monocyte_chemotactic_protein_3
135 Monocyte_chemotactic_protein_4
136 Monocyte_chemotactic_protein_5
137 Monocyte_chemotactic_protein_6
138 Monocyte_chemotactic_protein_7
139 Muscle_Glycogen_0imp_g
140 Muscle_Glycogen_0_nmolgl_g_2h
141 Muscle_Glycogen_250imp_g
142 Muscle_Glycogen_250nmolgl_g_2h
143 muscle_triglycerides_mmol_g
144 number_of_fetuses_in_left_horn_of_the_uterus
145 number_of_fetuses_in_right_horn_of_the_uterus
146 number_of_pups_in_first_litter
147 OGTT_of_0_mmoll_glycemia
148 OGTT_of_0_nmoll_insulin
149 OGTT_of_120_mmoll_glycemia
150 OGTT_of_30_mmoll_glycemia
151 OGTT_of_30_nmoll_insulin
152 OGTT_of_60_mmoll_glycemia
153 OGTT_of_60_nmoll_insulin
154 placental_weights_g_in_left_horn_of_the_uterus
155 placental_weights_g_in_right_horn_of_the_uterus
156 plasma_ALP_UL
157 plasma_K_mmolL
158 plasma_Mg_mmolL
159 plasma_P_mmolL
160 plasma_urea_mmolL_10_wks_old_males
161 PNMT
162 Protein_percentage_in_epididymal_fat
163 protein_percentage_in_epididymal_fat_1
164 protein_percentage_in_epididymal_fat_2
165 pulse_pressure_determined_by_direct_puncture_of_the_carotid_artery_under_light_ethe_anaesthesia_in_12_week_old_males_fed_a_normal_lab_chow_at_generations_F16_F17
166 relative_kidney_weight_in_5_week_old_males
167 relative_kidney_weight_in_8_week_old_males
168 relative_left_ventricle_weight_determined_in_12_week_old_males_fed_a_normal_lab_chow_at_generations_F16_F19
169 relative_weight_of_the_fat_pad
170 residual_MAP_mm_Hg
171 serum_ACTH_levels_after_20_min_of_immobilization_stress_in_10_weeks_old_males
172 Serum_CgA
173 Serum_CgB
174 serum_Chga_chromogranine_A_fragments_ml
175 serum_Chgb_chromogranine_B_fragments_ml
176 Serum_chloride
177 serum_cholesterol_concentrations_10_weeks_old_fed_a_diet_with_60per_fructose_from_8_weeks_to_10_weeks_male
178 serum_cholesterol_concentrations_in_male_rats_8_weeks_old_fed_a_normal_lab_chow
179 serum_cholesterol_concentrations_in_male_rats_9_
180 serum_corticosteron_levels_after_120_min_of_immobilization_stress_in_10_weeks_old_males
181 serum_corticosteron_levels_after_20_min_of_immobilization_stress_in_10_weeks_old_males
182 serum_corticosteron_levels_after_40_min_of_immobilization_stress_in_10_weeks_old_males
183 serum_corticosteron_levels_after_80_min_of_immobilization_stress_in_10_weeks_old_males
184 serum_C_reactive_protein_mg_ml
185 serum_cysteine_mmolL
186 serum_cystglycine_mmolL
187 Serum_dopamine
188 serum_dopamine_ngml
189 Serum_Epinephrine
190 serum_epinephrine_ngml
191 serum_glutathione_mmolL
192 serum_HDL2_cholesterol_concentrations_in_11_week_old_males_fed_a_high_fat_high_cholesterol_diet_for_4_weeks
193 serum_HDL2_cholesterol_concentrations_in_7_week_old_males_fed_a_standard_chow
194 serum_HDL2_phospholipid_concentrations_in_11_week_old_males_fed_a_high_fat_high_cholesterol_diet_for_4_weeks
195 serum_HDL2_phospholipid_concentrations_in_7_week_old_males_fed_a_standard_chow
196 serum_HDL3_cholesterol_concentrations_in_7_week_old_males_fed_a_standard_chow
197 serum_HDL3_phospholipid_concentrations_in_7_week_old_males_fed_a_standard_chow
198 serum_HDL_cholesterol_concentrations_in_male_rats_10_weeks_old_fed_a_diet_with_60per_fructose_from_8_weeks_to_10_weeks
199 serum_homocysteine_mmolL
200 serum_IDL_cholesterol_concentrations_in_11_week_old_males_fed_a_high_fat_high_cholesterol_diet_for_4_weeks
201 serum_IDL_phospholipid_concentrations_in_11_week_old_males_fed_a_high_fat_high_cholesterol_diet_for_4_weeks
202 serum_IDL_phospholipid_concentrations_in_7_week_old_males_fed_a_standard_chow
203 serum_insulin_nmol_L_0_min_during_OGTT
204 serum_insulin_nmol_L_120_min_during_OGTT
205 serum_insulin_nmol_L_60_min_during_OGTT
206 serum_insulin_nmol_L_nonfasted_10_wks
207 serum_LDL_cholesterol_concentrations_in_11_week_old_males_fed_a_high_fat_high_cholesterol_diet_for_4_weeks
208 serum_LDL_cholesterol_concentrations_in_7_week_old_males_fed_a_standard_chow
209 serum_LDL_phospholipid_concentrations_in_11_week_old_males_fed_a_high_fat_high_cholesterol_diet_for_4_weeks
210 serum_LDL_phospholipid_concentrations_in_7_week_old_males_fed_a_standard_chow
211 serum_nitric_oxide_in_male_rats_fed_a_standard_chow
212 Serum_Norepinephrine
213 serum_norepinephrine_ngml
214 Serum_osmolarity
215 serum_Scg2_secretogranine_II_fragments_ml
216 Serum_SgII

217	Serum_sodium
218	serum_total_cholesterol_concentration_in_7_week_old_males_fed_a_standard_chow
219	serum_total_cholesterol_concentrations_in_11_week_old_males_fed_a_high_fat_high_cholesterol_diet_for_4_weeks
220	serum_total_phospholipid_concentrations_in_11_week_old_males_fed_a_high_fat_high_cholesterol_diet_for_4_weeks
221	serum_total_phospholipid_concentrations_in_7_week_old_males_fed_a_standard_chow
222	serum_triglyceride_concentrations_in_male_rats_8_weeks_old_fed_a_normal_lab_chow
223	serum_triglyceride_concentrations_in_male_rats_9_weeks_old_fed_a_diet_with_60per_fructose_from_8_weeks_to_9_weeks
224	serum_VLDL_cholesterol_concentrations_in_11_week_old_males_fed_a_high_fat_high_cholesterol_diet_for_4_weeks
225	serum_VLDL_cholesterol_concentrations_in_7_week_old_males_fed_a_standard_chow
226	serum_VLDL_phospholipid_concentrations_in_11_week_old_males_fed_a_high_fat_high_cholesterol_diet_for_4_weeks
227	serum_VLD_phospholipid_concentrations_in_7_week_old_males_fed_a_standard_chow
228	SgII
229	systolic_blood_pressure_determined_by_direct_puncture_of_the_carotid_artery_under_light_ether_anaesthesia_in_12_week_old_males_fed_a_normal_lab_chow_at_generations_F16_F17
230	systolic_blood_pressure_determined_by_direct_puncture_of_the_carotid_artery_under_light_ether_anaesthesia_in_12_week_old_males_fed_a_normal_lab_chow_at_generations_F16_F18
231	total_plasma_protein_gL
232	Triglyceride_Liver
233	Triglyceride_Muscle
234	Triglycerides_mmoll_decapitated
235	urine_adrenaline_concentration
236	urine_Ca_mmolL
237	urine_Cl_mmolL
238	urine_creatinine_mmolL
239	urine_dopamine_concentration
240	urine_K_mmolL
241	urine_Na_mmolL
242	urine_noradrenaline_concentration
243	urine_P_mmolL
244	urine_protein_g_L
245	urine_urea_mmolL
246	weeks_old_fed_a_diet_with_60per_fructose_from_8_weeks_to_9_weeks
247	Weight_Measure_during_Oral_Glucose_Tolerance_Test
248	weight_of_rats_males_12_weeks_old
249	wet_weight_of_copper_concentration_in_livers
